# Premature terminator analysis sheds light on a hidden world of bacterial transcriptional attenuation

**DOI:** 10.1186/gb-2010-11-9-r97

**Published:** 2010-09-29

**Authors:** Magali Naville, Daniel Gautheret

**Affiliations:** 1 Université Paris-Sud, CNRS, UMR8621, Institut de Génétique et Microbiologie, Bâtiment 400, F-91405 Orsay Cedex, France

## Abstract

**Background:**

Bacterial transcription attenuation occurs through a variety of *cis*-regulatory elements that control gene expression in response to a wide range of signals. The signal-sensing structures in attenuators are so diverse and rapidly evolving that only a small fraction have been properly annotated and characterized to date. Here we apply a broad-spectrum detection tool in order to achieve a more complete view of the transcriptional attenuation complement of key bacterial species.

**Results:**

Our protocol seeks gene families with an unusual frequency of 5' terminators found across multiple species. Many of the detected attenuators are part of annotated elements, such as riboswitches or T-boxes, which often operate through transcriptional attenuation. However, a significant fraction of candidates were not previously characterized in spite of their unmistakable footprint. We further characterized some of these new elements using sequence and secondary structure analysis. We also present elements that may control the expression of several non-homologous genes, suggesting co-transcription and response to common signals. An important class of such elements, which we called mobile attenuators, is provided by 3' terminators of insertion sequences or prophages that may be exapted as 5' regulators when inserted directly upstream of a cellular gene.

**Conclusions:**

We show here that attenuators involve a complex landscape of signal-detection structures spanning the entire bacterial domain. We discuss possible scenarios through which these diverse 5' regulatory structures may arise or evolve.

## Background

Transcription of protein-coding genes does not always lead to the production of full length mRNAs. In both eukaryotes and bacteria, transcriptome analysis is revealing high levels of short transcripts that result from either unsuccessful initiation events or premature termination [1-4]. In eukaryotes, the functions of such events remain unelucidated, except for a few cases [5], and abortive transcription is still largely considered as transcriptional 'noise'. In Bacteria however, a form of abortive transcription known as transcription attenuation has emerged as an important regulatory strategy. The basic principle of transcriptional attenuation is the folding of the RNA transcript into either of two alternative structures, one of them corresponding to a Rho-independent terminator. The expression/repression decision occurs through a sensing system located between the promoter and the first start codon of the operon, and depends on interactions modulated by a variety of signals. The type of signal detected is commonly used to classify attenuators into major families: riboswitches bind small metabolites [6-8], T-boxes bind tRNAs [9,10], and other types of 5' leaders respond to protein factors [11-13] or temperature [14-16]. The triggering signals, by reflecting the global physiological state of the cell, enable a continuous monitoring of operon expression requirements.

A number of computational strategies have been proposed for attenuator prediction. The most general approaches consist of the identification of mutually exclusive RNA secondary structures [17,18], with the limitation that they miss non-hairpin anti-terminators such as riboswitches whose anti-terminator corresponds to a much larger secondary structure. Other and more specific studies have been devoted to the screening of particular classes of attenuators, such as riboswitches [19-21], T-boxes [9] or leader peptide systems [22]. These screening strategies use either covariance models [19,20] or descriptors combining sequence and structure motifs [23], which are designed to detect conspicuous, class-specific signatures. Signatures can be, for instance, an RNA fold, a conserved sequence, or the presence of a short ORF [22]. Such screens based on sequence or structure recognition identify highly conserved members of a family and, eventually, closely related variants. For instance, five distinct riboswitch families have been described that all sense S-adenosyl methionine (SAM I [24-26], SAM II [27], SAM III [28], SAM IV [29] and SAM V [30]).

In bacterial species, up to 10% of operons may be regulated by transcription attenuation [17]. In agreement with this assessment, we showed in a previous study [31] that a mean of 1.6% of all bacterial genes could be subject to attenuation, with a maximum of 2.6% in Firmicutes. However, knowledge on transcriptional attenuation is unevenly distributed: almost none of the predicted attenuators in phyla such as Chlamydiae or Acidobacteria have associated functions, whereas 16% are already annotated in Firmicutes. As previous attenuator screens were mostly based on similarity searches, known families often present a marked homogeneity and lack many evolutionarily isolated instances. Moreover, they exclude entire classes of elements that are either too short or too variable to produce signatures strong enough for a similarity search.

To fully explore the variety of attenuation systems, we need strategies that do not rely initially on sequence or structure homology. We developed a protocol that first screens all potential Rho-independent terminators in the 5' region of genes in multiple bacterial genomes and, in a second stage, extracts significant elements using two types of procedures: a synteny-based procedure that seeks gene families with unusually frequent 5' terminators; and a non-syntenic procedure that seeks sequences conserved among multiple putative terminators. Synteny analysis alone was able to pick up every class of known attenuation system, which are generally the most widespread, while allowing the prediction of numerous new instances. A major benefit of this strategy lies in the evolutionary insight it provides on attenuator families, as we illustrate below for five families of particular interest found throughout the 302 species surveyed. We further characterized new attenuators, in particular in *Escherichia coli *and *Bacillus subtilis*, using sequence-based analysis. Our results demonstrate that attenuator characterization can be largely improved even in widely analyzed model species.

## Results and discussion

### 5' terminators are less stable than 3' terminators and unevenly distributed among species

We combined two methods for Rho-independent terminator prediction using either position weight matrices [32] or descriptors [33] to detect potential terminators in the 5' and 3' UTRs in 302 bacterial genomes (see Materials and methods for details). As already documented [34], the overall usage of Rho-independent terminators fluctuates among species, with a maximum in Firmicutes (approximately 2,689 predictions in *Bacillus cereus*) and a minimum in Actinobacteria (30 predictions in *Nocardioides *sp.) or in certain atypical species such as the proteobacteria *Ehrlichia ruminantium *(8 predictions), an obligate intracellular pathogenic organism. Controls performed using randomized UTR sequences indicate a low false positive rate in both 5' and 3' UTRs (2.3% and 2.5%, respectively; see Materials and methods). Based on experimentally verified *B. subtilis *operons, we estimate that our protocol would retrieve about 88% of 3' terminators [35].

To identify putative attenuators, we applied additional filters to the set of 5' terminators, based on orientation and distance relative to flanking genes. In our set of 302 bacterial genomes, this led to the identification of 15,930 putative attenuators, 1,004 of them overlapping sequences previously annotated as ORFs encoding short hypothetical proteins. In *B. subtilis*, this protocol detected 32 of 57 (56%) known attenuation systems (riboswitches, T-boxes and other elements).

Terminators found in 5' UTR regions are thermodynamically less stable than 3' terminators (average folding free energy of -16.5 kcal versus -20.2 kcal/mol, *P *< 2e-16) and their average stem length is slightly shorter (7.0 versus 7.6 bp, *P *< 2e-16). As seen above, this difference cannot be imputed to a higher false discovery rate in 5' UTR, although it is in agreement with expected properties of structures that must fold alternatively; all known 5' regulatory terminators allow an alternative readthrough, which is not the case for 3' terminators. Interestingly, 5' terminators do not seem to be evolutionarily more conserved in sequence than 3' terminators. Analyzing data from a recent screen for non-coding conserved elements in bacteria [36], we observed that 58% of 5' terminators, versus 66% of 3' terminators, overlap a conserved region. This suggests that 5' terminators are not associated with conserved sequences more often than canonical terminators.

### Synteny analysis reveals more than 50 gene families subject to frequent attenuation control

We classified 5' terminators based on homology relationships between downstream genes, an operation that amounts to seeking syntenic attenuator/gene pairs. This method is related to that of Merino and Yanofsky [17], who sought over-represented families of orthologous genes flanking putative attenuators. These authors defined attenuators based on mutually exclusive stems, while we look for single terminator motifs. In principle our approach should be more sensitive to transcriptional attenuators as some achieve their alternative state through contacts with external factors and do not require stable alternative base pairing. On the other hand, Merino and Yanofsky were able to detect translational attenuators, which we do not detect here.

**Figure 1:**
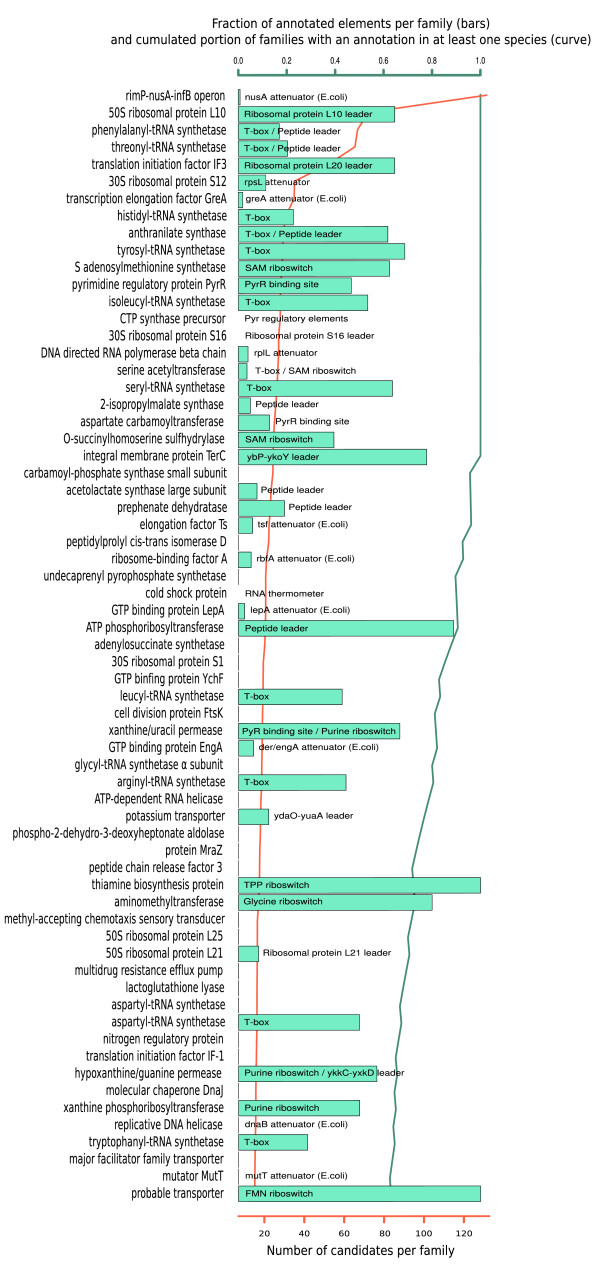
**Sixty-five families of genes most frequently controlled by attenuation**. For each gene family described on the left, the histogram bar shows the fraction of candidates already described in the Rfam database or in the literature. The green curve corresponds to the cumulated portion of families with at least one described candidate, from the first family to the 65th. The red curve indicates the absolute number of candidates in each family.

To identify protein families showing a greater propensity for regulation by transcriptional attenuation, we used the Hogenom database of gene families [37] and ranked families based on numbers of 5' terminators. In a first procedure, we scored gene families according to absolute numbers of predicted attenuators across all species without any consideration of gene family size, which favored large families of paralogs (Table S0 in Additional file 1). To avoid bias towards large paralog families, we used a second scoring procedure where gene families were ranked according to their frequencies and species distribution (Figure 1). The significance of attenuator enrichment was confirmed independently for each family. Scores, *P*-values and family information are shown in Table S1 in Additional file 1. Our synteny-based scoring eliminates over 90% of the false positives corresponding to terminators of independent small RNA genes. While 2.2% (343 elements) of the total 15,930 predicted 5' terminators map to annotated small RNAs, this fraction is reduced to 0.16% (3 out of 1,845) when considering only attenuator candidates from the 65 high-ranking families. Therefore, although some terminators of independent RNA genes are included in our initial screen, most of them are dismissed when we consider high-scoring gene families. Finally, we analyzed sequence conservation to detect possible functional elements associated with attenuators. To this aim, we performed pairwise sequence comparison of the terminator regions, followed by hierarchical clustering. Attenuator elements harboring a sequence conserved in at least two species are listed in Table S2 in Additional file 1 for the first 30 attenuator families.

We assessed prior knowledge of attenuator regulation in each gene family through a systematic literature survey and comparison to the Rfam RNA family database [38] and to the RegulonDB database of *E. coli *transcriptional networks [39]. The high incidence of known terminator systems among high-ranking families in Figure 1 underscores the specificity of our detection method. Forty-two out of 65 high-ranking families (65%) were already described as attenuator-regulated in at least one species, covering virtually all known classes of attenuator systems (Figure 1). This proportion reaches 100% for the first 20 families. Within known attenuator families, however, a large fraction of elements were not described previously: 67% of elements are unannotated in the top 30 families. Furthermore, several major families of attenuators are essentially uncharacterized: 27 out of 65 are completely uncharacterized or are less than 1% characterized. In the following sections we describe five previously uncharacterized attenuator families, selected either because they are particularly widespread or functionally interesting or because they display intriguing phylogenetic patterns.

#### The *rimP*-leader: the most ubiquitous transcription attenuator

The *rimP *gene ranks first in our list of genes most often regulated by attenuation (Figure 1). *rimP*, previously known as *yhbC *in *E. coli *and *ylxS *in *B. subtilis*, encodes a protein recently shown to be involved in 30S ribosomal subunit maturation [40]. It is the first gene of an operon encompassing the *nusA *and *infB *genes, which are present in almost all bacteria. While *infB *encodes the translation initiation factor IF-2, the NusA protein is characterized as a transcriptional pausing, readthrough, termination and anti-termination factor, and is shown to participate in the Rho-dependent anti-termination complex [41].

**Figure 2:**
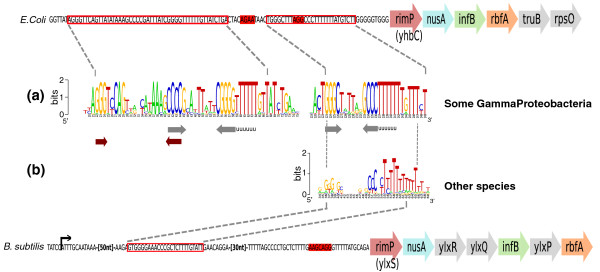
**The *rimP*-leader**. Highlighted boxes indicate putative ribosome binding sites. **(a) ***rimP*-leader identified in several Gammaproteobacteria (listed in Supplementary data 1 in Additional file 1), composed of a putative termination/antitermination structure (shown by thick arrows under the sequence) followed by the general *rimP*-leader motif described in the text. **(b) ***rimP*-leader found in the majority of species, including Firmicutes and Gammaproteobacteria. This leader sequence consists of a hairpin that is G-rich on its 5' arm and C-rich on its 3' arm, followed by the T-stretch characterizing Rho-independent terminators. The black arrow indicates the transcription start site recently detected by deep sequencing [46]. Sequence logos were produced using Weblogo [81].

Analysis of predicted attenuators in *rimP*-*nusA*-*infB *operons (Figure 2b) revealed the presence of a short and highly conserved motif corresponding to the terminator, the '*rimP-*leader', but gave no evidence of larger conserved elements characteristic of riboswitches, T-boxes or ribosomal protein-dependent attenuators. The terminator stem contains an unusual highly conserved GGGc(...)gCCC motif. We were unable to detect such a conserved motif in any other 5' or 3' terminator, suggesting this sequence signature is specific to the *rimP-*leader. We could find, however, the same motif along with the downstream U-stretch in many 5' UTRs of *rimP*-*nusA*-*infB *operons where no terminator structure was detected (Supplementary data 1 in Additional file 1). These additional motifs were missed because the potential hairpin was too short for detection with our programs, consistent with our previous observation that regulatory terminators are less stable than regular terminators. The distance separating the terminator from the gene start varies between 4 and 130 nucleotides, and may consequently encompass the ribosome-binding site (RBS). In several Gammaproteobacteria (listed in Supplementary data 1 in Additional file 1), the *rimP*-leader appears more complex, with a second terminator found in tandem and upstream of the former (Figure 2a), and presenting a clear potential anti-terminator structure. Interestingly, this terminator presents a CCCg(...)cGGG motif, inverse to the downstream motif.

High sequence conservation in the *rimP*-leader terminator stem and the absence of any visible antiterminator structure argue for regulation involving a termination or anti-termination protein that can specifically recognize a nucleic-acid motif [12,13]. If feedback control by RimP, now known to interact with rRNA [40], may be hypothesized, the best candidate for this direct interaction is probably the NusA protein, the expression of which was shown 25 years ago to repress expression of the operon [42,43]. NusA contains an amino-terminal domain that interacts with RNA polymerase, an S1 domain frequent in RNA-associated proteins, and two RNA-binding K homology (KH) domains [44]. It was already shown to be involved in the attenuation of the Trp, His and S10 operons [45] by interacting with the upstream arm of the terminator hairpin, but the RNA motif we describe here was not observed in these instances.

While this manuscript was under review, a deep sequencing study of 5' regulators in *B. subtilis *[46] observed that transcripts encoding certain core transcription elongation subunits, including *ylxS *(that is, *rimP*), appear to contain a long 5' leader region. The authors suggested these regions may contain elements regulating the associated genes. The 180-nucleotide leader they observed by deep sequencing in *B. subtilis rimP *transcripts indeed covers the attenuator we predict for this gene (Figure 2).

#### The *rpsL*-leader: a multiform ribosomal protein leader

**Figure 3:**
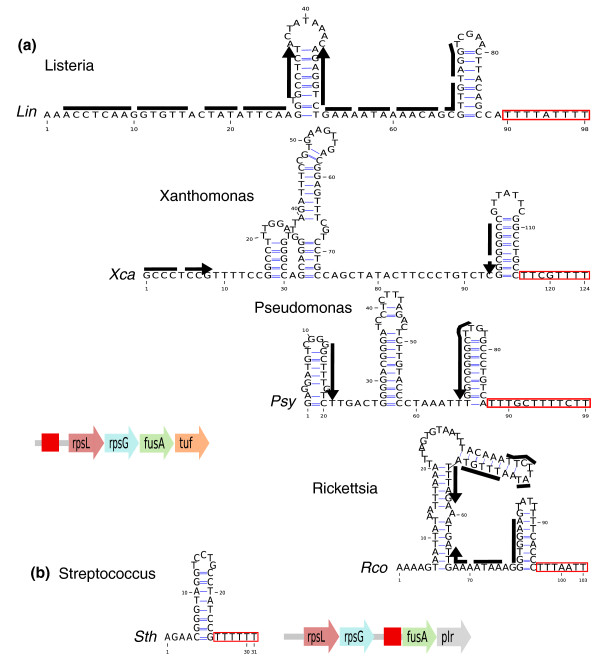
**The *rpsL*-leader**. Representative structures are shown for reference species, each corresponding to the consensus terminal structure of elements found in closely related species. Boxes indicate terminator T-stretches. Black arrows indicate putative anti-terminator structures. **(a) **Elements found upstream of the *rpsL *gene in *Listeria*, *Xanthomonas*, *Pseudomonas *and *Rickettsia *genera. **(b) ***rpsL*-leader found upstream of the gene *fusA *in streptococci.

The *rpsL *gene also appears at the top of the list of genes frequently regulated by transcriptional attenuation (Figure 1). Like *rimP*, it belongs to an operon of largely conserved genes, *rpsL *and *rpsG*, encoding the ribosomal proteins S12 and S7 respectively, and *fusA *and *tufA*, encoding the elongation factors EF-G and EF-Tu, respectively. Sequence-based clustering allowed us to identify variants among the different '*rpsL-*leaders' (Table S2 in Additional file 1). We derived consensus structures for several of these elements and were able to find potential alternative antiterminator structures in each case (Figure 3).

The translation of *rpsL *and *rpsG *was already shown to be controlled by S7, the product of *rpsG*, in *E. coli *[47], and Merino and Yanofsky [17] predicted putative transcription attenuators upstream of *rpsL *in 24 species. However, their results scantly overlap our own predictions: we have only four common candidates, while we scanned 22 of their 24 species. The occurrences we missed with our protocol involved non-canonical terminators (with a long, GC-poor and/or bulged hairpin), or terminators that were too far from the ATG to meet our distance filter (8 of 18 cases).

The persistence of an attenuator element upstream of *rpsL *in widely divergent species argues for a common origin. However, we found no globally conserved feature, neither in sequence nor in structure, associated with this attenuator. This probably explains why no common structure has been proposed for the *rpsL-*leader previously. In the *Streptococcus *genus, we found no attenuator upstream of *rpsL*, contrary to Merino's analysis, which found one (this terminator is too far from the ATG codon (189 nucleotides) to satisfy our distance filter). However, we found a candidate further downstream between *rpsG *and *fusA *in streptococci (Figure 3b). It is possible that two similar elements are present in this operon, since Meyer *et al. *[48] found two similar RNA structures upstream of *rpsL *and *fusA *in the Proteobacteria *Candidatus Pelagibacter ubique*. This would be consistent with co-regulation of the two genes. The element identified in [48], however, does not meet our terminator criteria and does not present any common sequence feature with any of our predicted attenuators.

Is the structure and sequence diversity in *rpsL*-leaders compatible with an interaction with the same S7 protein partner in all species? The highly flexible RNA binding portion of S7 [49] could tolerate some variation in RNA targets or, alternatively, the leader may bind different protein partners. In the current view of ribosomal protein leaders, each leader family displays a characteristic motif that mimics corresponding binding sites in ribosomal RNA. This view may be too restrictive and the example of *rpsL *suggests that the modalities of interaction may differ across distant phyla.

#### ABC-leaders: conferring specificity to regulatory elements

ATP-binding cassette (ABC) transporters constitute one of the largest and most ancient protein families, with hundreds of paralogs transporting a wide variety of substrates across the plasmic membrane, including ions, amino acids, lipids and drugs [50,51]. In Bacteria, these multiproteic complexes are encoded by operons comprising genes for ATPase, permease and periplasmic components. A number of them were shown to be regulated by transcriptional factors [52], whereas very few are known to be subject to transcriptional attenuation [53]. ABC transporters do not appear in Figure 1, where scores are weighted for family size; however, this family presents the highest absolute number of genes regulated by attenuation (Table S0 in Additional file 1), with a total of 205 candidates in our study, and an enrichment *P*-value of 8.8e-05. Further scrutiny of these candidates is important because of the great diversity of transporters and potential variability of regulatory elements controlling them. We found different sequence motifs associated with these 'ABC-leaders' (listed in Table S3 in Additional file 1).

**Figure 4:**
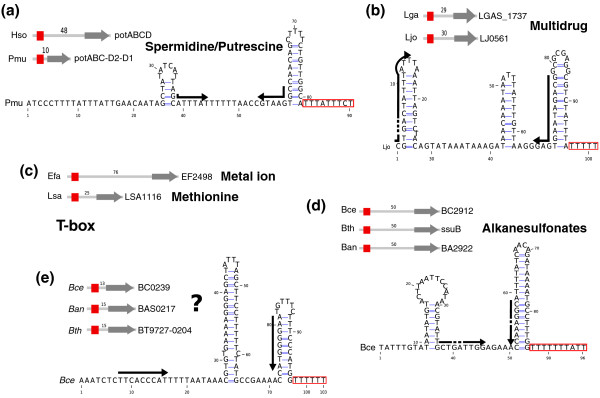
**ABC-leaders**. **(a) **The ABC-leader found upstream of the *potABCD *operon, encoding a spermidine/putrescine import system, in the Gammaproteobacteria *Haemophilus somnus *and *Pasteurella multocida*. **(b) **The ABC-leader found in the lactobacilli *Lactobacillus gasseri *and *Lactobacillus johnsonii*, upstream of a multidrug export system operon. **(c) **The T-box found in the Firmicutes *Enterococcus faecalis *and *Lactobacillus sakei*, upstream of genes for metal ion and methionine transporters, respectively. **(d) **The ABC-leader found in bacilli upstream of an alkanesulfonates transporter operon. **(e) **The ABC-leader found in bacilli 15-nucleotides upstream of a transporter of unknown specificity. Candidates are represented using the same conventions as in Figure 3.

Figure 4 shows five ABC-leaders associated with transporters of either known (Figure 4a-d) or unknown (Figure 4e) substrates. Each is able to form an antiterminator structure and the conserved sequence/structure motif (see alignments in Supplementary data 2 in Additional file 1) suggests that it responds to a unique substrate. The candidate shown in Figure 4c is a probable T-box, but the other candidates do not resemble any known *cis*-regulator. Their regulatory mechanisms thus remain to be determined. The size of the conserved structure is sufficient to form an aptamer that could directly detect a substrate, thus defining new classes of riboswitches; however, we cannot exclude an indirect regulation involving a protein factor. Indeed, a number a RNA-binding proteins target palindromic RNA [12,13] that may also act as a terminator hairpin.

The analysis of such a multiple paralog family raises interesting evolutionary questions on the origin of associated attenuators, that is, whether they all derive from an ancient attenuator that would have regulated the ancestral ABC transporter, or if certain genes of this family tend to 'attract' attenuators for their regulation. The relatively low proportion of attenuated ABC transporter genes and the ability of attenuators to 'hop' from one gene to another (see below) argue for the second hypothesis.

#### Regulators of the *hisS *genes: switching between sensing systems

Syntenic attenuation systems do not necessarily use the same sensing system. There are well known examples of switches from a T-box or a riboswitch in certain species (for example, Firmicutes) to a leader peptide in others (for example, Proteobacteria) [9,10]. Our protocol, which does not require a conserved RNA sequence or structure, is well suited to detect such exchanges, and at least five are present in our list of frequently attenuated genes (Figure 1).

**Figure 5:**
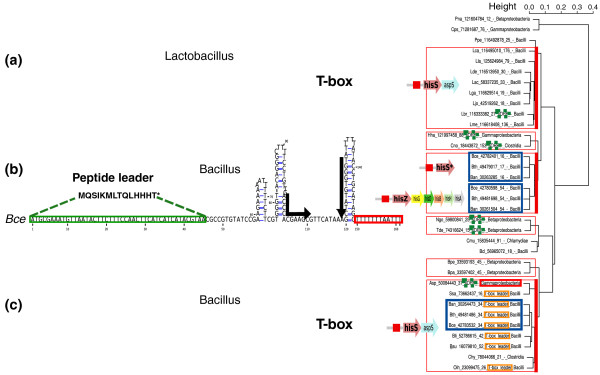
**Regulators of the *hisS *gene family**. The dendrogram on the right represents a hierarchical clustering of candidate attenuator sequences. Clusters of conserved sequences defined by a threshold E-value of 10^-4 ^are framed. Blue frames highlight three groups of paralogs found in bacilli. Dotted frames indicate candidates previously annotated as short hypothetical proteins ('sORF'). **(a) **A T-box found upstream of *hisS *in lactobacilli. **(b) **Histidine leader peptide identified in bacilli upstream of two paralogs of *hisS*. **(c) **A T-box found upstream of *hisS *in bacilli and other species, including isolated Gammaproteobacteria and Chlorobia.

A particularly striking case of switch between sensing systems is provided by the *hisS *gene (Figure 5). In the *Bacillus *genus, *hisS*, encoding the histidyl-tRNA synthetase, has been long known to be regulated by a T-box, like many other tRNA synthetases [9]. This gene, however, underwent two successive duplications: a recent one that appears specific to bacilli, and a more ancestral one that occurred before divergence of the Proteobacteria and Firmicutes. Interestingly, all three paralogs now found in bacilli are predicted to be regulated by a different type of attenuator, as shown in Figure 5. In addition to the known *hisS *T-box, we found novel attenuators upstream of hisS* (the *Bacillus hisS *paralog) and *hisZ*, encoding an ATP phosphoribosyltransferase regulatory subunit.

We performed a sequence-based clustering of the 5' UTR regions in the *hisS *family to identify sets of related motifs (Figure 5). The 5' UTRs of *hisZ *and of hisS* have highly similar sequences that include short ORFs encompassing a stretch of histidine codons. This strongly argues for the presence of a histidine leader peptide regulating both genes. To our knowledge, no leader peptide system had been shown to exist outside of Proteobacteria [22] and Actinobacteria [54], if we exclude the atypical *ermC *leader [55] that controls translation and has no amino acid specificity but senses a global slowdown in translation. This result thus strengthens the evolutionary relevance of leader peptide systems and expands the range of RNA-based regulation in Gram-positive bacteria.

Leader peptides may have spread to Firmicutes by horizontal transfer. However, we may also hypothesize that short reading frames may have emerged repeatedly from random sequences, especially in the favorable cellular environment of species such as those in the Firmicutes phylum. In support of convergent evolution, no similarity in the non-coding or leader peptide sequence is observed between the Firmicutes and Proteobacteria. That the two *hisS *gene duplications are observed only in certain Firmicutes species suggests a recent event: a gene resulting from the first duplication may have evolved or captured an attenuation system (for example, from a Gram-negative bacteria) before undergoing a second duplication.

Sequence analysis of *hisS *attenuators also reveals a T-box element in Lactobacillales (Figure 5a). Furthermore, we observed one possible horizontal transfer of attenuator elements from Firmicutes to Gammaproteobacteria: an unknown proteobacterial attenuator, which corresponds to a sequence encompassing a putative short ORF, clearly resembles Firmicute T-boxes (Figure 5c, red box). This illustrates the remarkable lability of attenuator elements, which can be acquired from other species and subsequently evolve to fit the preferred regulatory mechanisms of their new host.

#### The related *greA*- and *rnk*-leaders

**Figure 6:**
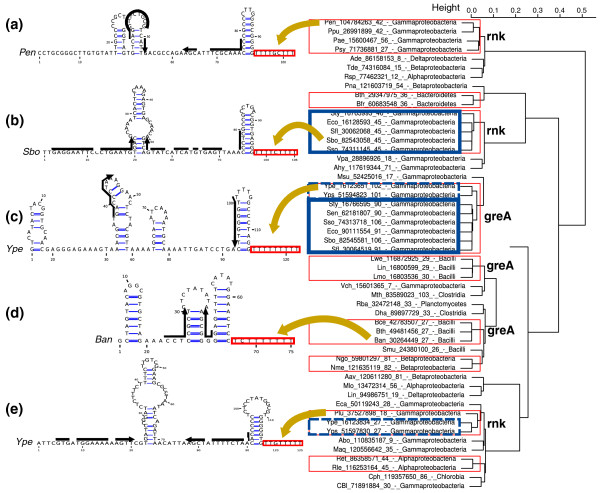
**The *greA*- and *rnk*-leaders**. **(a) **The *rnk*-leader identified upstream of *rnk *in *Pseudomonas*. **(b,c) **The *rnk*-leader and *greA*-leader identified in Enterobacteria, upstream of *rnk *and *greA*, respectively. **(d) **The *greA*-leader identified upstream of *greA *in bacilli. **(e) **The *rnk*-leader identified upstream of *rnk *in *Yersinia*. Candidates are represented using the same conventions as in Figure 3.

The *greA*/*rnk *gene family ranks seventh in our list of genes frequently regulated by attenuation. Although the propensity of *greA*/*rnk *genes for transcriptional attenuation was detected previously [17], experimental evidence for an *E. coli greA *attenuator is recent [4]. The gene family includes two major paralogs, *greA*, which encodes a transcription elongation factor, and *rnk*, which encodes a regulator of nucleoside diphosphate kinase. We found attenuators upstream of these genes in species ranging from Proteobacteria to bacilli and Clostridia. In several species, we identified attenuators in both genes. Figure 6 shows the result of a sequence-based clustering of *greA*/*rnk *attenuators and secondary structure models for different sequence clusters. In each case, we were able to detect a clear antiterminator structure; however, no common feature could be detected between the *greA*- and *rnk*-leaders.

Very interestingly, the limited experimental evidence available on these two putative *cis *non-coding RNAs argues for a second mechanism in *trans*. Potrykus *et al. *[4] showed that overexpression of the short form of the *greA *transcript, released after attenuation has occurred, leads to repression of several genes. Furthermore, an intergenic region corresponding to the *rnk*-leader was found in a systematic screening [56] to co-immunoprecipitate with Hfq, a conserved bacterial protein known to facilitate interaction between small RNAs and their target mRNA. Although leaders doubling as *trans*-acting RNAs is a recent and, for the time being, rare finding [57], we may have found here two such cases with the *greA*- and *rnk*-leader families.

### Identification of attenuator 'regulons'

The term 'regulon' or 'modulon' [58] has been coined to describe a set of genes subject to a common regulatory element. We analyzed attenuator regulons involving members of one or more gene families. To this intent, we compared sequences surrounding predicted 5' terminators across all genes with no consideration for orthology in a set of related species. We then clustered similar 5' sequences based on pairwise distances in so-called 'terminator clusters'. We describe here results obtained in the Enterobacteria (13 species) and *Bacillus *(8 species) subfamilies. Surveyed species are listed in Table S6 in Additional file 1. We identified a total of 192 and 270 terminator clusters in Enterobacteria and bacilli, respectively. We distinguished clusters based on the nature of downstream genes. Clusters involving only orthologous genes overlap the previous analysis and are given in Tables S4 and S5 in Additional file 1. We focus below on clusters involving several non-orthologous genes or groups of genes present in a single or a few related species.

#### 'Mobile attenuators' associated with transposable elements

**Figure 7:**
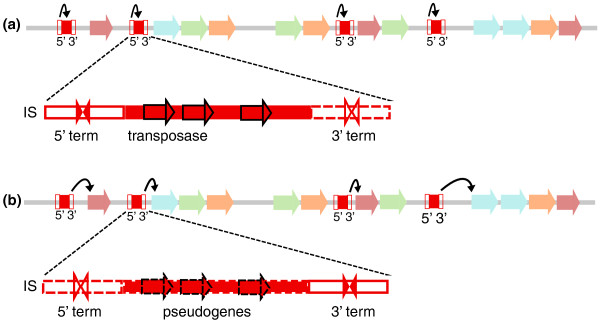
**Mobile elements and the emergence of new attenuators**. **(a) **5' terminators detected upstream of transposase or insertion sequence (IS)-related genes correspond to intrinsic IS 5' elements that prevent expression of the IS when it is inserted in a transcribed region. **(b) **ISs also contain intrinsic 3' terminators that terminate transcription of the 3'-most IS genes. When IS-related genes undergo pseudogenization, some 3' terminators may remain active as attenuators of downstream cellular genes (exaptation). As IS insertion may occur at nearly random genomic sites, similar attenuator candidates may be found upstream of totally unrelated genes.

Forty-six terminator clusters in Enterobacteria and 96 clusters in bacilli are associated with transposable elements. We describe them as 'mobile attenuators'. Terminator sequences in these clusters show a high level of conservation, consistent with an origin from transposable elements of recent dissemination. They fall into two classes. The first class (Figure 7a) corresponds to terminators located upstream of transposases or other insertion sequence (IS)-related genes and is mainly species-specific. Of note, transposase or IS-related genes also rank high in the list of frequently attenuated gene families, when no normalization for family size is applied (Table S0 in Additional file 1). These genes belong to transposon families such as ISBma2 (mainly present in the Betaproteobacteria *Burkholderia mallei*, in *Bacillus thuringiensis *and in the Clostridia *Symbiobacterium thermophilum*, *Thermoanaerobacter tengcongensis *and *Clostridium novyi*), IS3/IS2/IS600/IS1329/IS407A (found sporadically in all phyla) and ISL3 (mainly present in different Firmicutes species). The second class of mobile attenuators (Figure 7b) represents families of related transposon-borne sequences located immediately upstream of different, unrelated cellular genes.

The emergence of mobile attenuators can be explained by the structure of the IS containing both 3' and 5' transcription terminators [59,60] (Figure 7). Terminators of the first class correspond to IS 5' terminators, whose function is to limit transposon proliferation when inserted in a coding region under control of an active promoter. Such transposition events would be deleterious for the host, and consequently for the element's own survival. Clusters of conserved attenuators located upstream of unrelated genes (Figure 7b) may correspond to 3' terminators of ISs. The significant proportion (25 to 35%) of conserved terminator clusters that result from IS transposition suggests they have a significant impact in genome evolution, particularly in terms of regulation. The possible pseudogenization of transposed genes may allow exaptation of their 5' and/or 3' terminators and give birth to new regulatory elements (Figure 7b). It should be noted that this exaptation of transposable elements for regulatory functions is probably a universal mechanism, as several instances are already described in eukaryotic genomes [61,62].

#### Non-insertion sequence related regulons

**Figure 8:**
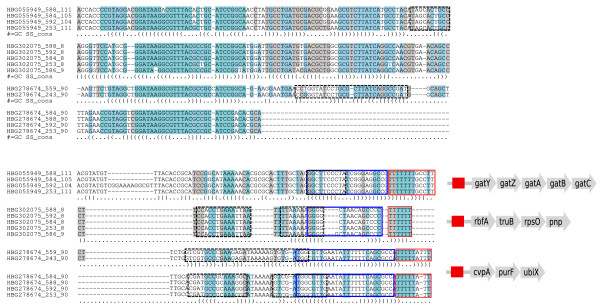
**A possible attenuator based on bacterial interspersed mosaic elements (BIMEs)**. A conserved element found before the *gatY*, *rbfA *and *cvpA *genes in Enterobacteria, corresponding to BIMEs. Blue frames indicate terminators, black frames indicate possible anti-terminators. Conservation of these repeats in sequence and position argues for their utilization as attenuator elements.

Ten terminator clusters in Enterobacteria and 23 in bacilli are found in different species associated with the same set of non-homologous genes. They may therefore constitute attenuator regulons (Tables S4 and S5 in Additional file 1). Several known riboswitches fall in this class (15 out of 23 clusters in bacilli), which is consistent with the ability of riboswitches to occur upstream of unrelated genes from distant species. Analysis of these clusters thus seems promising for the identification of new regulons, although this class also contains sequences from repeated elements. For instance, the largest cluster detected in Enterobacteria (Figure 8) involves elements conserved upstream of the *gatY*, *rbfA*, *cvpA *and, in some cases, *mscS*, *dppB*, *yidB *and *sbp *genes. Several members of this cluster present significant similarities with the rtT transcript identified by Bösl and Kersten [63], a by-product of the *tyrT *operon that could have a role in stringent response, supporting the biological significance of these candidate attenuators. Interestingly, we found these highly conserved elements to belong to bacterial interspersed mosaic elements (BIMEs) [64]. Even though this is a repeated element, it probably constitutes a *bona fide *attenuator as well. Indeed, BIMEs have been shown to act as bidirectional transcription terminators [65] and to induce transcriptional attenuation when placed upstream of a tRNA reporter gene [66]. The high conservation of these repeats, both in sequence and physical location, among Enterobacteria supports an exaptation scenario, possibly as an attenuator system. A second large attenuator cluster involving the genes *artJ*, *ygdL*, *yhfZ *and *yijO *was also found to correspond to BIMEs (Table S4 in Additional file 1).

**Figure 9:**
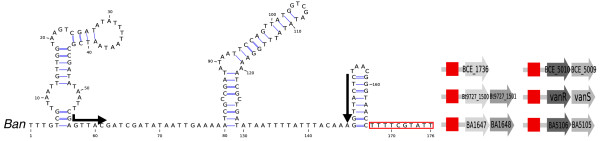
**The *vanR*-leader**. This element is conserved in bacilli, upstream of genes encoding a flavin oxidoreductase and a TCS DNA binding element (VanR). Candidates are represented using the same conventions as in Figure 3.

Clusters of attenuators located upstream of different genes suggest coexpression, and therefore functional relationships between these genes. In bacilli, an interesting such element, which we called the '*vanR*-leader', was found upstream of a gene encoding a flavin oxidoreductase and of the *vanR *gene, encoding a TCS transcription factor involved in vanillate catabolism (Figure 9; alignment in Supplementary data 3 in Additional file 1), suggesting a concerted regulation of these two genes. Another common element is found upstream of a sulfonate ABC transporter operon and upstream of an unknown gene. Its involvement in sulfonate metabolism may consequently be hypothesized (alignment in Supplementary data 3 in Additional file 1). Finally, a cluster of related attenuators, which we named '*fabH*-leaders', involves elements upstream of the *fabH *gene, involved in fatty acid metabolism, and upstream of genes encoding a DNA binding protein, for which an association with the same pathway would be interesting to test (alignment in Supplementary data 3 in Additional file 1).

## Conclusions

### Broad-spectrum detection techniques are required to capture a larger fraction of attenuator diversity

In this study we apply a detection protocol that is able to capture a large spectrum of 5' regulatory sequences. Analysis of associated genes allowed us to list genes most frequently controlled by attenuation, and to further describe their attenuators. While previous computational screens based on sequence/structure homology were successful at identifying many distinct elements such as T-boxes or riboswitches [9,10,19-21], they tend to suggest a discrete occurrence of specific elements, while the reality looks more as a continuous landscape of evolving elements that explore all possible sensing systems offered by 5' UTR regulation. An illustration of this diversity is provided by the extensive analysis released by Weinberg *et al. *[67], who screened all available bacterial genomes for structured RNA and unearthed more than a hundred new significant motifs, most of which are probable *cis*-regulators. Strikingly, none of these motifs overlaps our major attenuator families in Figure 1. Indeed, many of the sensing systems used by attenuators do not involve an RNA structure or evolve too rapidly to be caught by conservation analysis. Here we captured them using a simple terminator motif that, although it is not present in all *cis*-regulatory RNAs (some use translational control or cryptic terminators), was able to detect a variety of elements not represented in current databases or literature.

An obvious limitation of our protocol is that it misses cases of Rho-dependent termination. However, only one instance of a Rho-dependent terminator-based attenuator has been described to date, in *E. coli *[68]. We can furthermore hypothesize that regulation more likely uses Rho-independent structures, which do not need any additional protein to function and thus may be more responsive to external challenges. Another limitation of computational attenuator detection is that it cannot unambiguously discriminate attenuators from independent non-coding RNAs when the later meet all search criteria (that is, if they are terminated by a Rho-independent structure and located directly upstream of a gene in the same orientation). The main non-experimental clue to this question could lay in the synteny conservation between different strains: linkage of attenuator element and flanking gene strongly argues in favor of a *cis*-acting element. However, as some *trans*-acting small RNAs tend to retain their physical location for co-regulation reasons, no absolute rule can be drawn.

Our analysis should result in a better annotation of bacterial intergenic regions. It is likely, for instance, that many short ORFs currently annotated as hypothetical proteins and located upstream of operons, which we purposely considered as intergenic sequences, will turn out to be leader peptides or other attenuation systems. Indeed, among the top 30 families of Figure 1, 14 candidates from 8 different families were previously annotated as short hypothetical proteins, which was probably erroneous.

### A harvest of mobile terminators and clues for possible exaptative scenarios

From an evolutionary viewpoint, it is interesting that unrelated organisms may use different sensing systems to regulate the same genes. We observed such interchanges in most of the families we studied. A recurrent scenario that was previously documented is the switch between a T-box in Firmicutes and a leader peptide in Proteobacteria [9,10]. One of our noticeable finding, however, is the presence of 'regular' leader peptides in Firmicutes, replacing T-boxes.

**Figure 10:**
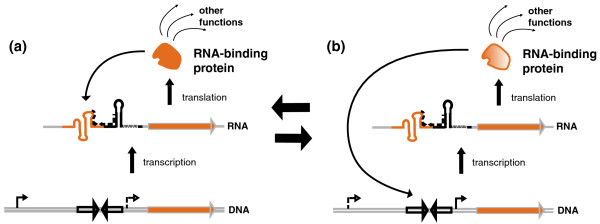
**Models for the evolution of self-regulation by RNA- and DNA-binding proteins**. **(a) **RNA-binding protein. Among other cellular functions involving interactions with RNA molecules, RNA-binding proteins often regulate their own expression by interacting with the 5' UTR of their mRNA. In the case of transcriptional attenuation, the transcription start site in the DNA is followed by a palindrome corresponding to the terminator. Emergence of an additional transcription start site downstream of the terminator palindrome could give birth to a new transcription factor binding site. **(b) **DNA-binding protein. These proteins (mainly transcriptional factors) often regulate their own expression through interaction with a palindromic DNA sequence in the promoter. Emergence of an additional transcription start site upstream of this transcription factor binding site palindrome could give birth to a new transcriptional attenuator.

Interestingly, we noticed a tendency for RNA-binding protein genes to be more often regulated by attenuation than other genes: while about 5% of *B. subtilis *operons present a predicted attenuator, this proportion reaches 14% among operons encoding at least one RNA-binding protein (data not shown). As several RNA-binding proteins are now shown to regulate their own expression at the transcriptional level (for example, ribosomal proteins [11], PyrR [12] or *E. coli *threonyl-tRNA synthetase [69]), it is tempting to postulate that many other RNA-binding proteins may harbor such dual-functionality, as illustrated in Figure 10a. In the same way, DNA-binding factors tend to regulate their own expression at the DNA- level (Figure 10b) [70,71]. It is tempting to consider possible evolutionary shifts between these two models. A number of attenuator elements identified in this study were previously assigned to palindromes interacting with transcription factors at the DNA level (MarR- or LysR-binding sites for instance). The switch from one system to another is conceivable with the emergence of a second transcription start site upstream or downstream of the palindrome sequence. Such tandem transcription start sites are quite frequent [72,73] and may give rise to bifunctional elements acting both as terminator and transcription factor binding sites (Figure 10) and possibly interacting with two different protein partners. It would be interesting to test such a hypothesis. Other potentially bifunctional terminators would involve sequestration of a RBS by the terminator hairpin, inducing a concerted transcriptional and translational attenuation. While no such case has been experimentally demonstrated to date, it is noticeable that 18 candidate attenuators in *E. coli *are located under 15 nucleotides from the start codon, three of which are annotated as RBS-sequestrator in the RegulonDB database [39]. A switch from one system to the other is therefore a realistic hypothesis.

## Materials and methods

### Identification of putative elements involving a Rho-independent terminator

Genome sequences and ORF annotations were retrieved from the National Center for Biotechnology Information (NCBI) database. The list of analyzed species and corresponding GenBank IDs is presented in Table S7 in Additional file 1. In a first stage, we looked for Rho-independent terminators in intergenic regions, as well as in regions encompassing short ORFs (<200 nucleotides) encoding hypothetical proteins as well as leader peptides (considered as a particular case of transcriptional attenuation), so as to potentially reannotate some of them. A terminator search was carried out using a combination of ERPIN [32] and RNAMOTIF [33]. ERPIN is a profile detection algorithm that takes as input a structure-annotated alignment. Here we used a training set of 1,200 terminator sequences from *B. subtilis *and *E. coli*. For the RNAMOTIF search, we used the descriptor of Lesnik *et al. *[74]. This descriptor consists essentially of a 4- to 18-bp helix, a 0- to 2-nucleotide spacer and a 12-nucleotide T-rich region. Parameters and input files for each program are given in Supplementary data 4 in Additional file 1. In this study, terminators were first sought using ERPIN, and in a second stage using RNAMOTIF when ERPIN was not able to detect any motif. In tests conducted with randomized terminator sequences, the combination of these two programs produced, on average, 47 false positive hits per 1,000 intergenic regions of size 115 nucleotides [35]. In this study, we further compared the specificity of terminator detections in 5' and 3' UTR sequences by generating, for each predicted terminator, two sets of 100 randomized sequences of the same di-nucleotide composition as 5' and 3' UTRs, respectively, and applying our prediction protocol to each set.

To be considered as candidate attenuators, terminators had to be in the same orientation as the downstream gene and at a suitable distance to avoid incorporation of canonical 3' terminators (from the upstream gene or from an undetected non-coding RNA present in the intergenic region). To this effect, terminators located at a distance ratio (that is, the ratio of the distance from the downstream gene to the distance from the upstream gene) greater than 1:1 or further than 300 nucleotides from the first codon of the downstream gene were discarded. Final predictions are listed in Table S8 in Additional file 1.

### Annotation of attenuator candidates and genes

Attenuator candidates were defined as the sequences from the limit of the upstream gene - or a maximum of 200 nucleotides - to the position immediately 3' of the terminator. Candidates were assigned to Rfam 10.0 [38] families by similarity search using Blast [75] with the following parameters: -p blastn, -G 2, -E 1, -W 7, -e 0.0001. Hits were retained if the match covered more than 50% of the full length of the Rfam entry. Candidates that failed to match a Rfam family with Blast were further submitted to covariance search using the rfam_scan.pl script provided on the Rfam web site [38]. Attenuator candidates that could not be annotated using these methods were submitted to a literature search based on the function of the downstream gene, or searched in the RegulonDB [39] for *E. coli *candidates. We assigned functions to bacterial genes using BlastP against the Hogenom database [37] and retrieving the corresponding Hogenom IDs.

### Hogenom family scoring

Each Hogenom family of homologous genes [37] was scored in two ways. The absolute score was defined as the absolute number of predicted attenuators upstream of all genes in the family across all 302 species (Table S0 in Additional file 1). The normalized score was computed as follows: for a given Hogenom family, the number of candidates in each species was normalized by the total number of genes in this species for this family. These single-species scores were then summed across all species. Lastly, in order to favor evolutionarily parsimonious distributions, we arranged single-species scores in a phylogenetic order and weighted consecutive non-zero scores by a factor of +0.1 times the length of the non-zero stretch. We confirmed the statistical significance of the enrichment of 5' terminators in each Hogenom family using Fisher's exact tests with the following values: number of genes in the Hogenom family; total number of genes assessed; number of genes with 5' terminators in the Hogenom family; total number of genes with 5' terminators.

### Clustering of attenuator sequences

For sequence analysis, we extracted regions around putative Rho-independent terminators, from the limit of the upstream gene (or a maximum of 200 nucleotides) to the position immediately 3' of the terminator. The distance between two sequences was defined as the inverse of the best hit score obtained by Blast/bl2seq [75]. We then performed hierarchical clustering from the distance matrix using the 'hclust' procedure from the R package [76]. Clusters were defined by pruning the tree at a height of 0.038 (corresponding to an E-value of approximately 10^-4^) determined empirically so as to cluster exclusively elements known to belong to the same family. For each cluster, we performed a multiple sequence alignment using ClustalW [77] followed by consensus structure prediction with the RNAalifold program [78].

RNA alignments and structures were refined and analyzed using the Ralee Emacs extension [79]. RNA structures were drawn using the Varna applet [80].

## Abbreviations

ABC: ATP-binding cassette; BIME: bacterial interspersed mosaic element; bp: base pair; IS: insertion sequence; ORF: open reading frame; RBS: ribosome-binding site; TCS: two-component system; UTR: untranslated region.

## Authors' contributions

MN and DG both contributed to the experimental design, data analysis and writing of the manuscript. MN performed the experiments.

## Supplementary Material

Additional file 1 **Supplementary tables and figures**. Table S0: gene families showing the highest absolute numbers of attenuator candidates. Table S1: genes most frequently regulated by attenuation in bacteria (normalized by family size). Table S2: list of sequence clusters observed in the 30 gene families most often regulated by attenuation (tabulation-separated). Table S3: sequence clusters obtained among candidates upstream of ABC-transporter genes. Table S4: complete list of clusters obtained by analyzing all candidates from enterobacterial species listed in Table S6. Cluster classes: 'a', clusters including only orthologous genes. 'b', clusters including only non-orthologous genes, sometimes from a single species; 'c', 'super-clusters' containing several sets of orthologous genes. Table S5: complete list of clusters obtained by analyzing all the candidates of *Bacillus *species listed in Table S6. 'a', clusters including only orthologous genes; 'b', clusters including only non-orthologous genes, sometimes from a single species; 'c', 'super-clusters' containing several sets of orthologous genes. Table S6: list of species analyzed for the identification of attenuators 'regulons'. Table S7: complete list of analyzed species, along with GenBank identifiers of corresponding DNA molecules and clade. Table S8: complete list of attenuators predicted in 5' UTR of genes, using the protocol described in [31] (tab-delimited table). Supplementary data 1: list of *rimP*-leaders from Gammaproteobacteria; list of *rimP*-leaders from other species; list of intergenic regions where no terminator could be detected, but showing sequence similarity to putative attenuators. Supplementary data 2: Stockholm alignments of the five ABC-leaders shown in Figure 4. Supplementary data 3: lists and Stockholm alignments of attenuator 'regulons' (candidates present upstream of several non-homologous genes) in Firmicutes. Supplementary data 4: parameters, commands and descriptor files used for terminator prediction. Click here for file
