# Supplementary material for: Premature terminator analysis sheds light on a hidden world of bacterial transcriptional attenuation
Source: Genome Biol. 2010 Sep 29;11(9):R97. doi: 10.1186/gb-2010-11-9-r97 (PMC2965389; doi:10.1186/gb-2010-11-9-r97)
Supplement: Additional file 1 — Supplementary tables and figures. Table S0: gene families showing the highest absolute numbers of attenuator candidates. Table S1: genes most frequently regulated by attenuation in bacteria (normalized by family size). Table S2: list of sequence clusters observed in the 30 gene families most often regulated by attenuation (tabulation-separated). Table S3: sequence clusters obtained among candidates upstream of ABC-transporter genes. Table S4: complete list of clusters obtained by analyzing all candidates from enterobacterial species listed in Table S6. Cluster classes: 'a', clusters including only orthologous genes. 'b', clusters including only non-orthologous genes, sometimes from a single species; 'c', 'super-clusters' containing several sets of orthologous genes. Table S5: complete list of clusters obtained by analyzing all the candidates of Bacillus species listed in Table S6. 'a', clusters including only orthologous genes; 'b', clusters including only non-orthologous genes, sometimes from a single species; 'c', 'super-clusters' containing several sets of orthologous genes. Table S6: list of species analyzed for the identification of attenuators 'regulons'. Table S7: complete list of analyzed species, along with GenBank identifiers of corresponding DNA molecules and clade. Table S8: complete list of attenuators predicted in 5' UTR of genes, using the protocol described in [31] (tab-delimited table). Supplementary data 1: list of rimP-leaders from Gammaproteobacteria; list of rimP-leaders from other species; list of intergenic regions where no terminator could be detected, but showing sequence similarity to putative attenuators. Supplementary data 2: Stockholm alignments of the five ABC-leaders shown in Figure 4. Supplementary data 3: lists and Stockholm alignments of attenuator 'regulons' (candidates present upstream of several non-homologous genes) in Firmicutes. Supplementary data 4: parameters, commands and descriptor files used for terminator prediction. [file gb-2010-11-9-r97-S1.ZIP › Suppl_data/SuppData4.pdf]

Naville et al. Supplemental Data 4.

ERPIN and RNAMOTIF Commands and Input files

The following commands were used:

```
erpin rho-indep.epn seq.fa -1,4 -add 1 4 2 -pcw 1 -cutoff 90%

rnamotif -descr term_RNAmotif.txt seq.fa | rmprune
```

seq.fa is the genomic sequence to be searched.

Files rho-indep.epn and term_RNAmotif.txt are provided below.

rho-indep.epn (ERPIN input file)

```
>collect 11 true coli terminators; 12 rounds of erpin w/cutoff 5; 1 round erpin cutoff 14 vs coli and bsub post-CDS (-20+100 nt after STOP). FP: S=14: 15 hits in 10 Mb coli terms, 17 in 10 Mb bsub terms; S=10: 210/260; S=5: 2640/2990. True P: S=14: 443 coli/741 bsub;  S=10: 633/931; S=5: 859/1149
11111111111222222223333333333333333333333333333332222222254444444444444
>190->255 235  [colik12-term-20+100.fasta] FW 34-79 21.43
CACAGAAAAAAGCCCGCACctgaca------------------------GTGCGGGC-TTTTTTTTTCGAC
>3734->5020 5000  [colik12-term-20+100.fasta] FW 33-83 18.90
ATCTCAATCAGGCCGGGTTtgcttttatgc-------------------AGCCCGGC-TTTTTTATGAAGA
>5234->5530 5510  [colik12-term-20+100.fasta] FW 18-61 14.16
TAAATGACAAATGCCGGGTaaca--------------------------ATCCGGCA-TTCAGCGCCTGAT
>26277->27227 27207  [colik12-term-20+100.fasta] FW 38-81 19.13
TTATGCGAAAATGCCGGTCttgt--------------------------TACCGGCA-TTTTTTATGGAGA
>28374->29195 29175  [colik12-term-20+100.fasta] FW 65-107 14.63
TTGATTATAAAGGGCTTTAatt---------------------------TTTGGCCC-TTTTATTTTTGGT
>30817->34038 34018  [colik12-term-20+100.fasta] FW 63-106 16.13
AATAACTAATACGGTTCTCtgat--------------------------GAGGACCG-TTTTTTTTTGCCC
>84191->85312 85292  [colik12-term-20+100.fasta] FW 15-78 15.32
CGCTAACTGATTGCAGAATaggtcagacatgaatgtctggttt------ATTCTGCA-TTTTTTATTGAAT
>106557->107474 107454  [colik12-term-20+100.fasta] FW 36-103 16.37
CGTATAAAATTCGACTGGCaaatctggcactctctccggccaggtga--ACCAGTCG-TTTTTTTTTGAAT
>108279->110984 110964  [colik12-term-20+100.fasta] FW 31-72 19.24
TTGAAGTAAAAGGCGCAGGat----------------------------TCTGCGCC-TTTTTTATAGGTT
>113444->114487 114467  [colik12-term-20+100.fasta] FW 18-67 16.01
TAATCTCCCAACGCTGGCGtggagcaaca--------------------CGCCAGCG-TTATCCCATCCCA
>125695->127587 127567  [colik12-term-20+100.fasta] FW 17-60 20.36
GTAAGTAAAAGAGCCGGCCcaac--------------------------GGCCGGCT-TTTTTCTGGTAAT
>127912->129336 129316  [colik12-term-20+100.fasta] FW 44-86 17.15
GCAATTAAAAAAGCGGCTAacc---------------------------ACGCCGCT-TTTTTTACGTCTG
>131615->134212 134192  [colik12-term-20+100.fasta] FW 45-95 19.66
AAACGACACAATGCCCGGTgaatgagatt--------------------CCCGGGCATTTTTTTATTTCTA
>137083->138633 138613  [colik12-term-20+100.fasta] FW 30-73 15.55
ATTCGGAAATATGCCCGGAtctc--------------------------TCCGGGCA-TTAAACTGATGAC
>169778->170575 170555  [colik12-term-20+100.fasta] FW 32-85 15.16
CCTCTTATTTCGCGCCGTCgactgttaacggc-----------------GATGGCGCTTTCTCCGTTGTTA
>180884->182308 182288  [colik12-term-20+100.fasta] FW 20-67 16.35
ATCTCCCTCAACCCCTTCCtgaaaacg----------------------GGAAGGGG-TTCTCCTTACAAT
>190857->191708 191688  [colik12-term-20+100.fasta] FW 21-65 17.19
TTATCAAAAAGGAGCCGCCtgag--------------------------GGCGGCTTCTTTTTGTGCCCAT
>192872->193429 193409  [colik12-term-20+100.fasta] FW 28-88 19.00
AACGACAAAAACGCCGCTCagtagatccttgcggatcggc---------TGGCGGCG-TTTTGCTTTTTAT
>200971->201996 201976  [colik12-term-20+100.fasta] FW 37-97 15.06
CGCCAAACTTTACGGCCTGtctcattcttacgattgcgg----------CAGGCCGTGTTATTATTGTCGT
>208621->209580 209560  [colik12-term-20+100.fasta] FW 29-72 17.81
AGTTCTGAAAAAGGGTCACttcg--------------------------GTGGCCCT-TTTTTATCGCCAC
>229167->229970 229950  [colik12-term-20+100.fasta] FW 20-61 15.45
AGCCTCTCTGACAGCTCCTcc----------------------------GGGAGCTG-TTTTTACATGCTC
>231926->232549 232529  [colik12-term-20+100.fasta] FW 17-63 20.37
ATAAATAAAAAAGGCACCGgggaat------------------------CGGTGCCT-TTTTATTATCTGG
>240343->240816 240796  [colik12-term-20+100.fasta] FW 26-67 16.58
GGATAAAGAAACGGAGCCTtt----------------------------CGGCTCCG-TTATTCATTTACG
>255977->256435 256415  [colik12-term-20+100.fasta] FW 19-61 17.91
AATCTTTTCAACGCCTGGCact---------------------------GCCGGGCG-TTGTTCTTTTTAA
>272086->273216 273196  [colik12-term-20+100.fasta] FW 2-66 15.61
GGAAATTCTTCTCGGCTGActcagtcatttcatttcttcatgtt-----TGAGCCGA-TTTTTTCTCCCGT
>282425->284392 284372  [colik12-term-20+100.fasta] FW 27-81 14.20
TAAGAGATAAAACCGTCTGcggaatatttcccg----------------CAGACGGCTTTGTTGTTTTTGA
>290724->291455 291435  [colik12-term-20+100.fasta] FW 70-115 15.04
AATACAAATAAGGCCTTCTcactg-------------------------AGAAGGCCATTACCGACTTACA
>302215->302829 302809  [colik12-term-20+100.fasta] FW 28-70 16.51
AATGATAAAAATGGCGCAAata---------------------------CAGCGCCA-TTTTTATAGGTTA
>328687->330720 330700  [colik12-term-20+100.fasta] FW 25-76 15.55
TATTGCATAAAACCGCGCCatgtctgcatat------------------GGCGCTGT-TTCTCCTACCTCT
>392239->393642 393622  [colik12-term-20+100.fasta] FW 53-100 15.22
AATCTAAATAATGCCCGTCaaggattt----------------------GACGGGCA-TTACTGCAAAGGA
>407401->407685 407665  [colik12-term-20+100.fasta] FW 16-63 14.58
TGTAATTCCTCGCCTTCCCcttgaac-----------------------GGGAGGGCATTTTTCTGAAATA
>418815->420134 420114  [colik12-term-20+100.fasta] FW 31-78 18.84
ATTTGTTTTAACCACGGGGctgcgatg----------------------CCCCGTGG-TTTTTTATTGTGT
>428729->429700 429680  [colik12-term-20+100.fasta] FW 34-77 18.57
GATGTTGAAAATCCCGGTCagaa--------------------------GATCGGGA-TTTTTTTTGATGT
>453663->454013 453993  [colik12-term-20+100.fasta] FW 67-107 16.78
TTTTATGAAAAACGGCCTG------------------------------CGGGCCGTTTTGTTTTGTCTGG
>454357->455655 455635  [colik12-term-20+100.fasta] FW 43-92 15.67
```

```
ATTCGCACAAAGGCCCGTCaccgccaggt--------------------GGTGGGCT-TTTTTTTGTCATG
>456650->457924 457904  [colik12-term-20+100.fasta] FW 42-86 18.12
GTTAACCAAAAAGGGGGGAttta--------------------------TCTCCCCT-TTAATTTTTCCTC
>458112->460466 460446  [colik12-term-20+100.fasta] FW 42-96 18.40
AATAAAAACAGGGCTGGCAggctaattcgggct----------------TGCCAGCCTTTTTTTGTCTCGC
>460675->460947 460927  [colik12-term-20+100.fasta] FW 45-88 17.45
CGAAGTTCAAGGGCGCATCtact--------------------------GATGTGCC-TTTTTTATTTGTA
>461139->463010 462990  [colik12-term-20+100.fasta] FW 58-97 16.19
TAACAAGAAAAGGTCGCTT------------------------------TCGCGGCC-TTTTCCATTTCTG
>466624->467454 467434  [colik12-term-20+100.fasta] FW 26-67 15.93
TCCCTTCCAGCACCGGGCAat----------------------------TGCCCGGT-TTTTTTTGCGTTG
>469860->471641 471621  [colik12-term-20+100.fasta] FW 44-95 14.19
TGCAAAAATGTAACGCACTgtgcactgtcat------------------AGTGCGTT-TTCATTTTCAAAC
>472190->473476 473456  [colik12-term-20+100.fasta] FW 26-77 18.47
ACTGCAAAAAACAGCCGGAcggttttcacc-------------------TCCGGCTATTTTTTTAATTGTG
>494344->496218 496198  [colik12-term-20+100.fasta] FW 16-66 17.88
CCTGATGTAATGCCGGATGaccttcgtgt--------------------CATCCGGCATTTTTCTTTTCAT
>532235->533050 533030  [colik12-term-20+100.fasta] FW 22-63 15.49
GTCTGTCGCATCCCGCTCTgc----------------------------GGAGCGGG-TTTTTTTGACAAA
>538371->539732 539712  [colik12-term-20+100.fasta] FW 24-68 14.12
GGCCCCTGCAATGCCCGTCcttgc-------------------------GGCGGGCA-TTCTCCGGTTAAG
>601182->602558 602538  [colik12-term-20+100.fasta] FW 54-105 18.16
ACCATCCGAAACCGCTCTCatccattcgat-------------------GAGAGCGGTTTTTTTAATTACT
>613380->617261 617241  [colik12-term-20+100.fasta] FW 28-78 18.52
TTATTTATAAACCCATAATtacagaaaata-------------------ATTATGGG-TTTTTTATTTGTT
>638946->640541 640521  [colik12-term-20+100.fasta] FW 40-87 16.49
GCAATTGCTTAGCCGCCGGggtcaaac----------------------CTGGCGGC-TTTTTTATGGCAT
>653085->653765 653745  [colik12-term-20+100.fasta] FW 19-66 16.51
GACATGATAAAGCCGGAGGaaacttg-----------------------CCTCCGGCATTACTATTTACTT
>656515->656724 656704  [colik12-term-20+100.fasta] FW 33-73 18.68
AGAATTCAAAACCCGCTTAa-----------------------------TCAGCGGG-TTTTTTTTGGTCT
>658170->658373 658353  [colik12-term-20+100.fasta] FW 56-97 19.47
GATACAAAAAACCCGCTTCaa----------------------------AAAGCGGG-TTTTTTATCAGAC
>703167->705113 705093  [colik12-term-20+100.fasta] FW 60-106 17.86
AACAATGACAAGCGGTGGAgatcttc-----------------------TCTGCCGC-TTTTTTTTTCATC
>709013->709339 709319  [colik12-term-20+100.fasta] FW 55-98 17.87
TTGCATAAAAAAGCCAACCcgca--------------------------GGTTGGCT-TTTCTCGTTCAGG
>709013->709339 709319  [colik12-term-20+100.fasta] FW 56-98 15.54
TGCATAAAAAAGCCAACCCgc----------------------------AGGTTGGCTTTTCTCGTTCAGG
>712781->714421 714401  [colik12-term-20+100.fasta] FW 28-71 20.35
TAATAAAAAAAGGGCGGTCgcaa--------------------------GATCGCCC-TTTTTTACGTATG
>728357->728563 728543  [colik12-term-20+100.fasta] FW 36-81 20.44
TACATTGAAAACCGCCAGCggaaac------------------------GCTGGCGG-TTTTTTGTATCAG
>819107->819811 819791  [colik12-term-20+100.fasta] FW 29-72 15.01
CACCATCACTATCCCGGCCatg---------------------------TGTCGGGATTTTTTTCTTACTA
>834471->835433 835413  [colik12-term-20+100.fasta] FW 20-69 14.84
ATTCTTTTCTCAGCCGGATgagccatgtc--------------------ATCCGGCT-TTTACCCCACCGC
>849673->850188 850168  [colik12-term-20+100.fasta] FW 31-72 20.82
GATATAAAAAATCCGCCTCtc----------------------------GGGGCGGA-TTTTTGTTTTTAA
>852870->853988 853968  [colik12-term-20+100.fasta] FW 28-70 19.23
CAAATAAAAAAGGCGGATTatg---------------------------AGTCCGCC-TTTTGCTTTAAGT
>877471->877854 877834  [colik12-term-20+100.fasta] FW 17-61 17.69
CTGAAAACTAAGGGGGGAGAaagcg------------------------TCTCCCCC-TTCATGTTTAAGT
>879950->881152 881132  [colik12-term-20+100.fasta] FW 21-69 14.83
TCTTCTGATAACCGGATGGcgcgaaacg---------------------TCATCCGG-TTATACGTCATTA
>882896->884128 884108  [colik12-term-20+100.fasta] FW 15-65 14.33
GGGTAAAAAAATGCCTGACtgctttgtgcg-------------------ATCAGGCA-TTCTCGAATTAAT
>896307->897152 897132  [colik12-term-20+100.fasta] FW 42-84 14.31
TGGCTACATAATGCCGCGCatg---------------------------TCGCGGCA-TTGTTTTCATGGA
>919570->921516 921496  [colik12-term-20+100.fasta] FW 25-69 18.58
TGAGATAAAAATGCCAGCCgatcg-------------------------GGCTGGCA-TTTTGCCTTTAGG
>938651->939943 939923  [colik12-term-20+100.fasta] FW 34-73 19.75
TGAATCTAAAAAGCGCCTG------------------------------CGGGCGCT-TTTTTTGTCTCCC
>943256->944119 944099  [colik12-term-20+100.fasta] FW 19-60 16.84
AATCATAACAACCGGGGTTtc----------------------------GGCCCCGG-TTCTCTTTTATTT
>945094->946242 946222  [colik12-term-20+100.fasta] FW 20-75 18.09
AATGAATTCAAGCAGAGTGtgaacttactgtttca--------------CACTCTGC-TTTTTTGTTTCTT
>948891->949481 949461  [colik12-term-20+100.fasta] FW 55-102 17.40
GGAATAAAAAAGCGGCAACtcaataaa----------------------GTTGCCGC-TTTACGGGGAAAT
>956876->957964 957944  [colik12-term-20+100.fasta] FW 29-73 17.10
TTTTGCTTAATCCCCACAGccagc-------------------------CTGTGGGG-TTTTTATTTCTGT
>958035->959318 959298  [colik12-term-20+100.fasta] FW 53-95 16.90
CTTTATCAAAACGTCGGCAcat---------------------------TGTCGGCG-TTTTTTTTCGGAC
>963051->963335 963315  [colik12-term-20+100.fasta] FW 39-79 17.47
AACGAGAGAAAAGCACCTGt-----------------------------CGGGTGCT-TTTTTCATTTCTC
>975549->980009 979989  [colik12-term-20+100.fasta] FW 15-58 19.20
AGTTAAAATTAAGGCGGCAgcaa--------------------------TGCCGCCT-TTTCTTTTTCCGA
```

```
>1003991->1005001 1004981  [colik12-term-20+100.fasta] FW 39-91 14.06
CTTTATAAGATAATCAGGGctttattttcag------------------CCCTGGTTGTTTTATATTCATC
>1014134->1014682 1014662  [colik12-term-20+100.fasta] FW 31-83 17.56
TTGTAAATATAACCGTCTCcggtatgttgcct-----------------GAGGCGGT-TTTTTTGTCTCTA
>1014938->1015105 1015085  [colik12-term-20+100.fasta] FW 31-71 17.06
TTAAAAAGAAACCTCCGCAt-----------------------------TGCGGAGG-TTTCGCCTTTTGA
>1017708->1018160 1018140  [colik12-term-20+100.fasta] FW 49-88 19.22
GGCAAAAAAAACCCCGCAG------------------------------CAGCGGGG-TTTTTCTACCAGA
>1027088->1027582 1027562  [colik12-term-20+100.fasta] FW 14-66 18.43
CAAATAAAAAATCCCCGGAaggcaaaaacct------------------TCCGGGGATTTGTTCAGGGATT
>1084215->1085279 1085259  [colik12-term-20+100.fasta] FW 27-69 14.38
TATCGGTGCAGAGCCCGGGcga---------------------------ACCGGGCT-TTGTTTTGGGTGT
>1099471->1100010 1099990  [colik12-term-20+100.fasta] FW 43-85 16.69
AACAGTAAAATGCCGGATGata---------------------------ATTCCGGC-TTTTTTATCTGTC
>1103670->1104125 1104105  [colik12-term-20+100.fasta] FW 31-72 16.45
GTATTACAGAAACAGGGCGca----------------------------AGCCCTGT-TTTTTTTCGGGAG
>1133952->1134734 1134714  [colik12-term-20+100.fasta] FW 16-60 14.44
TCTAAGGCTTAACCGGTGGcaggt-------------------------TCACCGGT-TTACTGATTTTTG
>1139256->1140209 1140189  [colik12-term-20+100.fasta] FW 51-103 16.75
TATGTTTTGTCTGCCTGCTctgggatcgctgg-----------------GGCGGGCA-TTTTTTTGCCTAT
>1151162->1152403 1152383  [colik12-term-20+100.fasta] FW 34-75 17.95
ACCCTTATAAAAGGTCCGCtt----------------------------GCGGGCCT-TTTTTCTTAGCTT
>1157092->1158525 1158505  [colik12-term-20+100.fasta] FW 34-75 17.05
GGGGAGACTAAGGCAGCCAga----------------------------TGGCTGCC-TTTTTTACAGGTG
>1161861->1162502 1162482  [colik12-term-20+100.fasta] FW 19-64 15.48
AATCCCATCACGCGCGACGaattgc------------------------TGTCGCGC-TTTTTCCCGCAGT
>1166822->1167361 1167341  [colik12-term-20+100.fasta] FW 32-82 17.16
TCTGCAATTTGGCCCCTCAttcgctcaggc-------------------TGAGGGGC-TTTTTTTGCGACT
>1168296->1168553 1168533  [colik12-term-20+100.fasta] FW 49-98 17.83
GCTGATTTTTTCCCCGCGAcatgccgtg---------------------TCGCGGGGATTTTTTTATCCGG
>1194346->1195596 1195576  [colik12-term-20+100.fasta] FW 35-77 18.56
AAATTTATTAACGGGAGCGtaa---------------------------CGCTCCCG-TTGTTTTTTGTTA
>1222918->1223130 1223110  [colik12-term-20+100.fasta] FW 24-65 14.76
TCAACGATTAAACTGCGCTtc----------------------------GGCGCAGT-TTTCGTTTACAGG
>1234161->1234880 1234860  [colik12-term-20+100.fasta] FW 32-71 16.24
TAAAGAGCAAACCCCTCAA------------------------------ACGAGGGG-TTTTTTGTTGTTT
>1243951->1244205 1244185  [colik12-term-20+100.fasta] FW 22-75 14.05
ATTTTCATAAGGCGGATAGcgatacagatgccg----------------CTATCCGC-TTTCACATCAGAA
>1285072->1285749 1285729  [colik12-term-20+100.fasta] FW 30-71 17.00
AGTTCACATAGACCCTGCTtc----------------------------GGCGGGGT-TTTTTTATGGGCA
>1290680->1291588 1291568  [colik12-term-20+100.fasta] FW 36-82 19.14
TATCCACGAAACGGCGTTGagcaat------------------------CGACGCCGTTTTTTTATAGCTT
>1303788->1304792 1304772  [colik12-term-20+100.fasta] FW 19-62 19.64
AAAAACAATAAGGGCTGACagtt--------------------------GTCAGCCC-TTTTTCACGCTAA
>1309113->1309832 1309812  [colik12-term-20+100.fasta] FW 24-63 16.66
GAAAGTCAAAAGCCTCCGA------------------------------CCGGAGGC-TTTTGACTATTAC
>1324876->1325751 1325731  [colik12-term-20+100.fasta] FW 18-62 16.79
TAATCAAAGTATGCCGGACgtcat-------------------------ATCCGGCA-TTTTTACAGATTA
>1327356->1328405 1328385  [colik12-term-20+100.fasta] FW 18-61 17.38
TAAAAGACAAACGCGAGGCtaag--------------------------ACCTCGCG-TTTTGCTTTAATC
>1329072->1331669 1331649  [colik12-term-20+100.fasta] FW 41-82 18.17
TTATAAACAAAGGGTCGCGaa----------------------------AGCGGCCC-TTTTTTATTGCAT
>1340679->1341008 1340988  [colik12-term-20+100.fasta] FW 18-61 17.37
TAACATAAAAAGCCACGGAtata--------------------------TCCGTGGC-TTTCGAATATTTT
>1367713->1368027 1368007  [colik12-term-20+100.fasta] FW 26-65 17.04
TTCACCATGAGCGGCGCTT------------------------------ATGCGCCG-TTTTTTTTTCTGT
>1383535->1384596 1384576  [colik12-term-20+100.fasta] FW 18-79 14.13
TAATGCAATATCGGGTGCTgaccggatatctttacgccga---------AGTGCCCGTTTTTCCGTCTTTG
>1426547->1427008 1426988  [colik12-term-20+100.fasta] FW 36-81 18.31
AGTGTTATGACAGCCCGCCggttca------------------------GGCGGGCT-TTTTTGTGGGGTG
>1445540->1447042 1447022  [colik12-term-20+100.fasta] FW 33-73 20.88
ATAAGTAAAAAACGGCACCt-----------------------------GGTGCCGT-TTTTTTGTCTGAA
>1462495->1463085 1463065  [colik12-term-20+100.fasta] FW 45-92 17.88
TTAATTATAAAGCAGAGTTatgttta-----------------------AGCTCTGCTTTATTTATTTGAG
>1468714->1472037 1472017  [colik12-term-20+100.fasta] FW 31-80 18.23
GTGATCCCTAAACCGCAACgctgatacag--------------------GTTGCGGT-TTTTTTATTGCCG
>1472245->1473105 1473085  [colik12-term-20+100.fasta] FW 21-66 17.55
CGAATATACAAAAGGGAAAgatgca------------------------TTTCCCTT-TTTTTCTTTTTTA
>1481142->1484987 1484967  [colik12-term-20+100.fasta] FW 36-87 18.79
GATAAAGAAAAAACCCGGTaagcatttagc-------------------GCCGGGTTTTTTATTAATTCTA
>1489701->1489874 1489854  [colik12-term-20+100.fasta] FW 54-98 17.10
GCTAGTCAAATGCGCGGGGaaaat-------------------------CCCCGCGC-TTGCCCTTACCTG
>1496732->1496899 1496879  [colik12-term-20+100.fasta] FW 20-60 16.59
AACTTTCCTAAGCCGCCAGc-----------------------------CAGGCGGC-TTTTTTAACAACT
>1500481->1501149 1501129  [colik12-term-20+100.fasta] FW 18-62 15.28
TAGTTCCAGACGCGCCATCgtta--------------------------GATGGCGCTTTTTATCCGGTGC
>1549362->1550015 1549995  [colik12-term-20+100.fasta] FW 35-79 15.63
```

```
TAAGCTGAAAATGGCGCTGtaaa--------------------------AGGCGCCATTTTCATATTGTAG
>1615052->1616242 1616222  [colik12-term-20+100.fasta] FW 15-54 16.13
CAATAGTTGAAAGGCCCAT------------------------------TCGGGCCT-TTTTTAATGGTAC
>1625541->1626287 1626267  [colik12-term-20+100.fasta] FW 18-59 14.13
TAATTTTTATACCCGGCGTaa----------------------------CTGCCGGG-TTATTGCTTGTCA
>1627239->1627442 1627422  [colik12-term-20+100.fasta] FW 19-59 16.08
AAGCGTGTGAATGCCGCCGa-----------------------------TGGCGGCA-TTGCTTTTTTACT
>1667723->1668976 1668956  [colik12-term-20+100.fasta] FW 18-70 14.48
TGAAAAATAAGTCCGGACTgcggtaaatacc------------------CGTCCGGACTTATTGCCAGCTC
>1669984->1670805 1670785  [colik12-term-20+100.fasta] FW 17-67 16.43
ATAATGTTCAAACGCTGCCcgacagcgcg--------------------GGCAGCGTCTTCATCAGGCAAG
>1676451->1677395 1677375  [colik12-term-20+100.fasta] FW 30-70 16.49
TACTCCTATAAGGCGGCTTg-----------------------------ATGCCGCC-TTTCGCATTTTTA
>1686600->1687775 1687755  [colik12-term-20+100.fasta] FW 68-111 14.02
TATAACACCTTCAGGCGGCcag---------------------------TCCGCCTGATTTCATTTTATGG
>1687876->1689384 1689364  [colik12-term-20+100.fasta] FW 23-70 15.50
CACGGCCAAAACGCCGGAGaatttca-----------------------CTCCGGCG-TTTTCATCTGAAA
>1700257->1701258 1701238  [colik12-term-20+100.fasta] FW 15-62 17.04
AAGTAACAAAATGGATGGTgcaaatgc----------------------ACCATCCA-TTTTTCATGCAAG
>1710793->1712295 1712275  [colik12-term-20+100.fasta] FW 33-79 18.92
CTCTTTTACAAGCCGCTAActtttcg-----------------------TTAGCGGC-TTTTTTTTTGTTC
>1739437->1740585 1740565  [colik12-term-20+100.fasta] FW 21-65 14.19
AGGCTATTCTATCGCCCCCtctcc-------------------------GGGGGCGA-TTTCAGATCAGGC
>1753722->1755134 1755114  [colik12-term-20+100.fasta] FW 41-82 20.28
TTGTATATCGAAGCGCCCTga----------------------------TGGGCGCT-TTTTTTATTTAAT
>1755445->1755681 1755661  [colik12-term-20+100.fasta] FW 31-73 20.43
GAAGTGAAAAATGGCGCACatt---------------------------GTGCGCCA-TTTTTTTTGTCTG
>1781001->1782701 1782681  [colik12-term-20+100.fasta] FW 34-83 18.24
GGATCACATAACCCCGGCGactaaacgc---------------------CGCCGGGGATTTATTTTATTTC
>1805820->1806680 1806660  [colik12-term-20+100.fasta] FW 27-67 15.46
TTGAGGATAATGGCCGCTCc-----------------------------GTGCGGCC-TTTTGATTAAATA
>1807404->1808072 1808052  [colik12-term-20+100.fasta] FW 13-64 17.29
TCGGTTAATGACCAGGGGCagtgatctcgct------------------GCCCCTGG-TTCTTTATCTGAA
>1860795->1861790 1861770  [colik12-term-20+100.fasta] FW 35-78 19.64
GTGATCTAAAAAGAGCGACttcg--------------------------GTCGCTCT-TTTTTTTACCTGA
>1866979->1868262 1868242  [colik12-term-20+100.fasta] FW 15-66 14.05
GGCTAAAACTATCAGCCAGgtcattatcgc-------------------CTGGCTGATTTTTAGCTTACTG
>1868409->1869884 1869864  [colik12-term-20+100.fasta] FW 27-69 14.82
AAATATTCAGAGCCATGCTttt---------------------------TGCGTGGC-TTTTGCATACAAT
>1879936->1881021 1881001  [colik12-term-20+100.fasta] FW 30-72 19.12
AGTTTATTTGAACCGCGTCact---------------------------GACGCGGT-TTTTTTATTCGTT
>1892097->1892456 1892436  [colik12-term-20+100.fasta] FW 40-93 15.66
AAATGGGTCAGGACGCTTTtaatcacatataa-----------------AAAGCGTCCTTTTTCTCACAAT
>1894956->1896320 1896300  [colik12-term-20+100.fasta] FW 18-70 14.28
TAATACTTCTTACTCGCCCatctgcaacggat-----------------GGGCGAAT-TTATACCCGCTTT
>1901910->1902770 1902750  [colik12-term-20+100.fasta] FW 26-68 18.41
TTGTACACTACCGGGGCCTtt----------------------------TGGCCCCGTTTTTTTATCTGGA
>1908189->1909673 1909653  [colik12-term-20+100.fasta] FW 14-59 20.20
GGCATAAAAAAAGCGCGTCgatcag------------------------GACGCGCT-TTTTAGTATTTAC
>1919789->1920040 1920020  [colik12-term-20+100.fasta] FW 21-61 15.20
TCCAAAAAGAACCCGTCGGc-----------------------------ATGGCGGG-TTATTTGTCCTGG
>1928905->1930083 1930063  [colik12-term-20+100.fasta] FW 35-79 19.23
CGCTACAAAAATGCCCGATcctcg-------------------------ATCGGGCA-TTTTGACTTTTAC
>1958086->1959819 1959799  [colik12-term-20+100.fasta] FW 31-80 17.16
GTGAGAGTGAAGCCTGATCagggttagcc--------------------GATCAGGC-TTTTTTATTGCCA
>1984948->1985451 1985431  [colik12-term-20+100.fasta] FW 54-107 18.77
GGTTTTGAGAAACCGCTGCctcatctgtttgaa----------------GCAGCGGT-TTTTTTAATGGGA
>1985467->1985805 1985785  [colik12-term-20+100.fasta] FW 77-118 15.71
GGACACAAAAAAACCTGCCgg----------------------------AGCAGGTT-TTTTGTTATCGGA
>1986739->1987236 1987216  [colik12-term-20+100.fasta] FW 17-63 14.42
CTAATGCTCTCCGGCGGCAggtttac-----------------------TGCCGCCG-TTTCAAATCAGTG
>1993841->1994065 1994045  [colik12-term-20+100.fasta] FW 16-68 19.38
AGTAATATAAAACCGGCTGatagcgtgccttt-----------------CAGCCGGT-TTTTGCATCTGGC
>2032861->2033265 2033245  [colik12-term-20+100.fasta] FW 53-106 16.79
ACTGAACACTAGCCTGAGTtcgtccctcatcga----------------ACTCAGGC-TTTTTTATTGGCA
>2033857->2034708 2034688  [colik12-term-20+100.fasta] FW 45-102 14.02
GCATAATCAATCGCGGCCGcgttagtactcacgacg-------------TGGCTGCGTTTTTTCACGGTTA
>2042885->2050036 2050016  [colik12-term-20+100.fasta] FW 31-79 16.10
TAATTTCTTAAAGAGTGTTccattcgg----------------------GGCACTCTTTTTTACATTCCCT
>2094636->2095247 2095227  [colik12-term-20+100.fasta] FW 59-106 16.04
GGCAAAAAAACGGGCAAGGtgtcacca----------------------CCCTGCCC-TTTTTCTTTAAAA
>2145633->2147048 2147028  [colik12-term-20+100.fasta] FW 18-81 17.72
TAATTGAAAAATGGCTCAGcagcggcttaaatctgcctgcgc-------CTGAGCCAGTTTTTCTGCTGCC
>2192320->2194353 2194333  [colik12-term-20+100.fasta] FW 21-64 16.92
TCCCCCTTCAAGGCGCTGCatcg--------------------------ACAGCGCC-TTTTCTTTATAAA
>2220205->2221920 2221900  [colik12-term-20+100.fasta] FW 60-115 14.43
AATCGTTCTGTGCCGTCTGccccgccgccatt-----------------TGGGCGGC-TTTTTGTTTTTTA
```

```
>2263215->2264042 2264022  [colik12-term-20+100.fasta] FW 30-73 17.21
CATGCAGAAAAGGGATAGCtcag-------------------------GCTGTCCC-TTTTTTAATTTAT
>2267999->2268565 2268545  [colik12-term-20+100.fasta] FW 58-101 18.36
AGTTAACTGAAAGCACTGCttag-------------------------GCAGTGCT-TTTTTGTTTTCAT
>2280537->2280821 2280801  [colik12-term-20+100.fasta] FW 31-72 20.57
AGTTGTAAAAAACCCCGCTcc---------------------------GGCGGGGT-TTTTTGTATCTGC
>2324129->2325313 2325293  [colik12-term-20+100.fasta] FW 20-69 18.05
ATCAATAAAAACACCCGATagcgaaagtt-------------------ATCGGGTG-TTTTCTTGAACAT
>2353541->2354731 2354711  [colik12-term-20+100.fasta] FW 22-74 15.50
CTCCTTTCTGATGCCCGGTaagcatgtggtt-----------------ACCGGGCATTTTTGCGTACACG
>2387133->2387984 2387964  [colik12-term-20+100.fasta] FW 49-94 15.18
TTTTCAGAAAAACCGCCGAgcatg------------------------TCGGCGGCTTTCTGACTTACAA
>2412767->2414911 2414891  [colik12-term-20+100.fasta] FW 50-96 18.11
GGATATCTGAACCGGAAATaatcact----------------------ATTTCCGG-TTTTTTATTCTCT
>2415080->2416621 2416601  [colik12-term-20+100.fasta] FW 19-61 20.36
AAAAATGTTAAGAGCCGCAat---------------------------TGCGGCTCTTTTTTCATTCTGC
>2493599->2494585 2494565  [colik12-term-20+100.fasta] FW 42-95 14.20
CTCCTCAAATCGGCCATAAtaacctcatggttt---------------TTATGGCT-TTTTTCTTAAGGT
>2516833->2517225 2517205  [colik12-term-20+100.fasta] FW 18-64 21.01
TAAATAAAAAACGGCAGGAtattatc----------------------TCCTGCCG-TTTATCTTTTTAC
>2529483->2530244 2530224  [colik12-term-20+100.fasta] FW 50-92 14.19
GTGTGAAACAGGGGTGGCTta---------------------------TGCCGCCCCTTATTCCATCTTG
>2530429->2531400 2531380  [colik12-term-20+100.fasta] FW 37-78 17.11
ATGCGTAAAAAAGCACCTTtt---------------------------TAGGTGCT-TTTTTGTGGCCTG
>2533854->2534363 2534343  [colik12-term-20+100.fasta] FW 29-71 18.45
GCAGTGAAAAATGGCGCCCat---------------------------CGGCGCCATTTTTTTATGCTTC
>2558277->2558918 2558898  [colik12-term-20+100.fasta] FW 17-61 15.21
ATAACGGAATCAGGCGGGCaatgt------------------------TCCCGCCT-TTTCTTTGCCTTA
>2562000->2562392 2562372  [colik12-term-20+100.fasta] FW 41-83 15.91
ACAACCAATACACCCGGCCcct--------------------------CGCCGGGT-TTTTTGTGATCTG
>2585615->2588728 2588708  [colik12-term-20+100.fasta] FW 21-66 18.20
GCAATAAAAAAGGCGACATgccaat-----------------------GTGTCGCC-TTTTTCAACTTTC
>2620254->2620892 2620872  [colik12-term-20+100.fasta] FW 27-70 18.38
CGTAATTAAAGCGCCAGCTctgc-------------------------CGCTGGCG-TTTTTCAATTCAC
>2623135->2624676 2624656  [colik12-term-20+100.fasta] FW 16-67 16.57
CTTAATACTTTGCGGGCCGacgagaaatgt------------------CGGCCCGCATTATTCAGGCACT
>2628346->2628885 2628865  [colik12-term-20+100.fasta] FW 53-99 18.24
GAAGATAAAAAACCCTCTGtagtaa-----------------------CAGAGGGTTTTGTTCATTCATA
>2650355->2651359 2651339  [colik12-term-20+100.fasta] FW 32-71 19.79
GCAATAAAAAAACCGCCGAA----------------------------TTTGGCGG-TTTTTTATTGCTA
>2671294->2671788 2671768  [colik12-term-20+100.fasta] FW 16-71 14.91
GTTAGGAAAGATGCCGGATgcggcgtgaacgccctt-------------ATCCGGCA-TTTAATAAATTAC
>2716755->2717174 2717154  [colik12-term-20+100.fasta] FW 16-60 14.67
TTTAATCTTACCGGGGCGCatctt------------------------GTGCCCCG-TTTTCTCCTCTGC
>2734166->2734903 2734883  [colik12-term-20+100.fasta] FW 29-74 21.02
TGAAACACAAAACGGCAGCccttga-----------------------GCTGCCGT-TTTTTTATTCTGT
>2735174->2735515 2735495  [colik12-term-20+100.fasta] FW 35-74 17.09
TGTATCGCCAACGCGCCTT-----------------------------CGGGCGCG-TTTTTTGTTGACA
>2735619->2735666 2735646  [colik12-term-20+100.fasta] FW 58-102 20.87
ACGAACAATAAGGCCTCCCaaatc------------------------GGGGGGCC-TTTTTTATTGATA
>2751815->2751967 2751947  [colik12-term-20+100.fasta] FW 41-81 19.42
ACGTTAAAAAAGGTGCTCAa----------------------------TGAGCACC-TTTTTTCTGTCTG
>2765725->2766594 2766574  [colik12-term-20+100.fasta] FW 39-87 18.63
TTATTCTCCGAGCCCTGTCgcaagtgc---------------------GGCAGGGCTTTCTTTATTTGTT
>2769169->2769636 2769616  [colik12-term-20+100.fasta] FW 36-85 15.38
TACCATAACAACCCTGATTgcctccggc--------------------GGTCGGGGGTTTCTTTTTTCTC
>2771339->2773042 2773022  [colik12-term-20+100.fasta] FW 17-56 15.74
GTAACGCATTTGGCTCCAA-----------------------------TTGGAGCC-TTTTTATGACTAT
>2798155->2798496 2798476  [colik12-term-20+100.fasta] FW 41-86 16.64
CGCGTCTTTGACCCCGAAGcctgt------------------------CTTCGGGGTTTCTTTTTGCCTG
>2810637->2812175 2812155  [colik12-term-20+100.fasta] FW 20-64 18.41
AGTACAACTAAGCCAGTTCattt-------------------------GAACTGGCTTTTTTCAATTAAT
>2863124->2864488 2864468  [colik12-term-20+100.fasta] FW 22-66 17.36
CTTAATAAAAAGGTCCGATgggc-------------------------ATCGGACCTTTTATTGTGCACA
>2903664->2904605 2904585  [colik12-term-20+100.fasta] FW 25-68 19.70
ACCATAAAAAATGCCAGCCcgga-------------------------GGCTGGCA-TTTTTAAATCAGA
>2927598->2928965 2928945  [colik12-term-20+100.fasta] FW 21-66 14.17
TCGCTCTCCAAAGGCCTCGttttg------------------------CGAGGCCTCTTCCCGATTTCTC
>2969619->2970659 2970639  [colik12-term-20+100.fasta] FW 16-57 16.67
CATAAGATTAACGCCGGTTaa---------------------------AACCGGCG-TTAAATATTAATG
>3001511->3001990 3001970  [colik12-term-20+100.fasta] FW 17-62 15.28
GTAAAAGGATATCCGGCCTgaattc-----------------------AGGCCGGA-TTCACTGAGGTTA
>3006785->3007996 3007976  [colik12-term-20+100.fasta] FW 27-70 14.63
TTCACTTATTCCCCTCCGGttcg-------------------------CCGGAGGG-TTTTTGGAGTTTG
>3025678->3026508 3026488  [colik12-term-20+100.fasta] FW 15-62 14.39
ATCTAATAGATTCTCCGCGcctttcag---------------------CGCGGAGA-TTTCTTTCAGAGA
>3084725->3085879 3085859  [colik12-term-20+100.fasta] FW 31-75 17.91
```

```
CCTGCGTTCAAAGGCCAGCctcgc-------------------------GCTGGCCT-TTTTCTTTTGGAT
>3089897->3090847 3090827  [colik12-term-20+100.fasta] FW 51-106 14.49
CTCTGAGTGACAGCGCCCTtctttccacgcatac---------------TGGGCGCTGTTGCTTTTTTGAA
>3103684->3104988 3104968  [colik12-term-20+100.fasta] FW 24-72 20.45
CGCAAAGAAAAACGGGTCGccagaagg----------------------TGACCCGTTTTTTTTATTCTTA
>3190880->3192541 3192521  [colik12-term-20+100.fasta] FW 28-74 19.81
TCCAGGAATAATCCCTGGCccaaaaa-----------------------GCCGGGGA-TTTTTTATATCTG
>3208422->3208637 3208617  [colik12-term-20+100.fasta] FW 57-107 16.29
GTAGTTGTAAGGCCGTGCTtccgaaaggaa-------------------TGCGCGGC-TTATTTTCGTTTA
>3210688->3212529 3212509  [colik12-term-20+100.fasta] FW 64-108 18.02
CCTCTGCACAAACGCCACCttttc-------------------------GGTGGCGT-TTTTTATCGCCCA
>3237584->3238828 3238808  [colik12-term-20+100.fasta] FW 18-66 16.34
TAATTGTTTAACCCCTTTCgtctacggc---------------------GGAAGGGG-TTTTCTCAACTTT
>3242744->3244162 3244142  [colik12-term-20+100.fasta] FW 41-82 18.18
TTCCCTTCAAAGCCGCCTTct----------------------------CAGGCGGC-TTTTTCATCACTG
>3248112->3248594 3248574  [colik12-term-20+100.fasta] FW 36-79 17.92
AACGATAAAAAACGAGGAGgaag--------------------------CTCCTCGT-TTTTGCTATTGGA
>3249944->3250309 3250289  [colik12-term-20+100.fasta] FW 23-69 15.54
CAAATCTGCTCGCCGGATGtctgact-----------------------CATCCGGC-TTTTATCATTATG
>3250551->3250907 3250887  [colik12-term-20+100.fasta] FW 20-61 15.19
ATCTTAGAATTGGGGCGATat----------------------------TTCGCCCC-TTTTTATTAACAA
>3272923->3274494 3274474  [colik12-term-20+100.fasta] FW 34-79 14.66
GTCCCTCGCAAAACATGGCcttagt------------------------GCCATGTT-TTTATTGTTTAAA
>3283119->3283910 3283890  [colik12-term-20+100.fasta] FW 17-66 14.20
GTAAATGGAACGAGGCACTgcgtctggt---------------------GGTGCCTCTTTACTTAAGGAAT
>3285067->3285651 3285631  [colik12-term-20+100.fasta] FW 18-70 16.26
TAATACGTTAGGGCGTTATctgacctgtcag------------------ATAACGCCCTTTTCCTTCCTCT
>3326604->3328037 3328017  [colik12-term-20+100.fasta] FW 20-64 16.04
GTCAAAAAGAAACCCCGGCacatg-------------------------GCCGGGGC-TTCAGATTATTGA
>3372504->3373871 3373851  [colik12-term-20+100.fasta] FW 28-68 16.31
AAAAGACAAAACAGGCCGCc-----------------------------TGGGCCTG-TTTTGTATTACTT
>3379835->3380902 3380882  [colik12-term-20+100.fasta] FW 32-73 19.57
AAAACAAAAAACCGGAGTCtg----------------------------TGCTCCGG-TTTTTTATTATCC
>3383122->3383436 3383416  [colik12-term-20+100.fasta] FW 37-77 15.10
CCGACGATATTGCCCCCGGt-----------------------------TCGGGGGC-TTTTTTTTGCGCT
>3403554->3404903 3404883  [colik12-term-20+100.fasta] FW 29-69 19.72
AAGCGTCAAAAGGCCGGATt-----------------------------TTCCGGCC-TTTTTTATTACTG
>3408908->3409204 3409184  [colik12-term-20+100.fasta] FW 39-88 20.12
TTGATTAAAAAGGCGCTACtcggcatggg--------------------GAAGCGCC-TTTTTTATAGGTG
>3412670->3415774 3415754  [colik12-term-20+100.fasta] FW 24-63 16.08
AGAAACATAAAGGCGCTTT------------------------------CGGGTGCC-TTTATTATTTCCA
>3416027->3416248 3416228  [colik12-term-20+100.fasta] FW 18-60 15.60
TAACTGAACAGGGCGCGGAgt----------------------------TCCGCGCTCTTTTTAACGATAC
>3420072->3420830 3420810  [colik12-term-20+100.fasta] FW 36-79 18.00
AAAAGCAAAAAGGCCATCCtttc--------------------------GGATGGCC-TTTCGCTTGATTT
>3431851->3432798 3432778  [colik12-term-20+100.fasta] FW 26-67 16.42
CACTCTTCTAAGCCCGGTCt-----------------------------TGCCGGGCGTTTTTATACTTAT
>3434155->3435531 3435511  [colik12-term-20+100.fasta] FW 17-56 15.83
GTAATTAATAAGGCGTCTA------------------------------ATGACGCC-TTATTATTTCCCT
>3475277->3475495 3475475  [colik12-term-20+100.fasta] FW 21-61 19.62
GGCGTAAAAAAAGCGGGGAt-----------------------------TCCCCGCT-TTTTTGTCACTTT
>3483757->3484389 3484369  [colik12-term-20+100.fasta] FW 21-69 15.34
TCCCGTCGGAGTGGCGCGTtacctggta---------------------GCGCGCCA-TTTTGTTTCCCCC
>3490205->3491386 3491366  [colik12-term-20+100.fasta] FW 18-68 17.26
TAATTGCTCATGCCGGACGgcactatcgt--------------------CGTCCGGCCTTTTCCTCTCTTC
>3494188->3494514 3494494  [colik12-term-20+100.fasta] FW 18-63 16.19
TAATGTTTTAACGGGAGGCgcaat-------------------------GCCTCCCCTTTTTGCATGGTCC
>3520485->3523061 3523041  [colik12-term-20+100.fasta] FW 16-56 17.04
TCTGATTAAAAAGGCGCTT------------------------------CGGCGCCTTTTCAGTTTGCTGA
>3527406->3528290 3528270  [colik12-term-20+100.fasta] FW 18-65 15.55
TAATGAGTATGTCCGGCAGagagggct----------------------CTGCCGGA-TTTTACGACTAAT
>3530456->3532078 3532058  [colik12-term-20+100.fasta] FW 58-103 19.40
AGAAAGCAAAACGGGAGGCaccttc------------------------GCCTCCCG-TTTATTTACCCTT
>3540364->3540600 3540580  [colik12-term-20+100.fasta] FW 35-86 14.90
CTGTTTTGGAAAGCCCGGTatgcgtctgca-------------------TCCGGGCTTTTTTGCGTGCGGC
>3584574->3585014 3584994  [colik12-term-20+100.fasta] FW 56-113 14.76
ATGCAATCCACACCCCAGCgcagcaactctgctgcgc------------GCTGGGGT-TTATTGACGGTAT
>3607532->3608143 3608123  [colik12-term-20+100.fasta] FW 26-79 15.17
AAGATGATGAGGCGGCCTCagggacgtgttccg----------------GAGGCCGT-TTTTTTAATCGCC
>3632372->3633523 3633503  [colik12-term-20+100.fasta] FW 29-82 17.04
TACCGGATAAAACCAGCGTtgaccatttgcgta----------------ACGCTGGT-TTTTCTTAGGCAT
>3637741->3638175 3638155  [colik12-term-20+100.fasta] FW 36-77 19.25
GTGTTCCTGAACGCCCGCAta----------------------------TGCGGGCG-TTTTGCTTTTTGG
>3638492->3639961 3639941  [colik12-term-20+100.fasta] FW 26-73 15.84
CTTGCAGAAAGCAGGGTAGcgttatcg----------------------CTACCCTG-TTTTAGTTTTACA
>3643929->3645281 3645261  [colik12-term-20+100.fasta] FW 18-68 16.73
TAAATGTTAAAGGGCTAAGagtagtgtgct-------------------CTTAGCCC-TTAATTACGTTTC
```

```
>3648921->3649703 3649683  [colik12-term-20+100.fasta] FW 2-66 15.61
GGAAATTCTTCTCGGCTGActcagtcatttcatttcttcatgtt-----TGAGCCGA-TTTTTTCTCCCGT
>3714927->3715913 3715893  [colik12-term-20+100.fasta] FW 29-75 14.85
TGCGTAGAGTAAAGCCCGAtaatcgc----------------------TCGGGCTT-TTACTCTTTATTG
>3717678->3717890 3717870  [colik12-term-20+100.fasta] FW 38-82 19.95
AGAATCTAAGATCCCTGCCattt-------------------------GGCGGGGATTTTTTATTTGTT
>3723516->3724511 3724491  [colik12-term-20+100.fasta] FW 16-65 17.03
GTTAAGTAAAAGCCCGGTCacattggact-------------------GACCGGGC-TTACGTGAGTTAT
>3794575->3795834 3795814  [colik12-term-20+100.fasta] FW 26-71 14.68
AACAAGTTTAAGAAGTGAGttaaaa-----------------------CTCACTTC-TTATCTATACAAC
>3834580->3835764 3835744  [colik12-term-20+100.fasta] FW 35-90 14.64
CTAAGAAAAATCGGCGTGGcgcaaacagagccat--------------CCTCGCCATTTTTGCTATCATG
>3854042->3854491 3854471  [colik12-term-20+100.fasta] FW 23-73 16.56
AGCCTGAATAATGCCCGCCgggtgatcatc------------------AGCGGGCA-TTATCGTCAGAAC
>3882705->3884351 3884331  [colik12-term-20+100.fasta] FW 35-77 20.14
GCTAAAATAAGGGCGGTCAgt---------------------------TGACCGCCTTTTTTCTTTTCGT
>3886344->3887774 3887754  [colik12-term-20+100.fasta] FW 20-73 14.59
ATTAATACTACAGAGTGGCtataaggatgtta----------------GCCACTCTCTTACCCTACATCC
>3887865->3889112 3889092  [colik12-term-20+100.fasta] FW 19-65 15.02
AAATCCTTCAAGAAGCCAGccattcg----------------------CTGGCTTC-TTGCCTCTCAGGA
>3892281->3892847 3892827  [colik12-term-20+100.fasta] FW 17-68 19.12
CTAAATAAAAAACCCGCCAgcaatcatgca------------------TGGCGGGTTTTTAACGCGCTAT
>3945709->3946047 3946027  [colik12-term-20+100.fasta] FW 13-55 15.28
ACGATTAATAAAAAGGGCGaaa--------------------------TGCCCTTT-TTTTATGTCAGTA
>3947945->3948043 3948023  [colik12-term-20+100.fasta] FW 32-83 18.14
TAACGAACTAAGACCCCCGcaccgaaaggtc-----------------CGGGGGTT-TTTTTTGACCTTA
>3950107->3951036 3951016  [colik12-term-20+100.fasta] FW 20-65 17.07
AATACAAAAAAATGGGACGGcacgca----------------------CCGTCCCA-TTTACGAGACAGA
>3955591->3957066 3957046  [colik12-term-20+100.fasta] FW 67-110 15.14
AAAACACTCAAGCCTTCTCctg--------------------------GAGAAGGCCTTGCTATTAGTTG
>3964032->3965291 3965271  [colik12-term-20+100.fasta] FW 29-72 19.42
TATGCCAAAAACGCCACGTgttt-------------------------ACGTGGCG-TTTTGCTTTTATA
>4010643->4012904 4012884  [colik12-term-20+100.fasta] FW 17-64 17.20
GTAAAATCCAAACCGGGTGgtaatac----------------------CACCCGGTCTTTTCTCATTACA
>4040999->4041625 4041605  [colik12-term-20+100.fasta] FW 30-73 20.98
AATTACATAAAGCCCGTGAgtat-------------------------TCACGGGC-TTTTTTATTATTT
>4083596->4084429 4084409  [colik12-term-20+100.fasta] FW 20-71 16.09
ATTAAAATAAGTCCTGCGAaatatattaaa------------------TTGCAGGACTTATTCATTTCGT
>4098391->4099011 4098991  [colik12-term-20+100.fasta] FW 67-109 17.83
TAAAAAATGAACCATCGCCaac--------------------------GGCGGTGG-TTTTTTTGTGATC
>4103532->4103900 4103880  [colik12-term-20+100.fasta] FW 50-93 18.88
ATCCCTGTCTTCCCCCACAtgc--------------------------TGTGGGGGTTTTTTTTATCCTC
>4106414->4107403 4107383  [colik12-term-20+100.fasta] FW 32-78 19.13
TAATTATCAAACCCGGTGGtttctcg----------------------CGACCGGG-TTTTTTATTTGTC
>4116095->4116340 4116320  [colik12-term-20+100.fasta] FW 71-110 15.64
CGCAGAAAAAAGGCACCTT-----------------------------GCGGTGCC-TTTCTTATCATTC
>4124593->4124805 4124785  [colik12-term-20+100.fasta] FW 28-67 18.99
TTTCCGAAAAAAGCGCCGC-----------------------------ACGGCGCT-TTTTTTGTGCCTG
>4154429->4155802 4155782  [colik12-term-20+100.fasta] FW 34-76 17.10
TATAAATTTGAGCCTGGCTtat--------------------------CGCCGGGC-TTTTTTATGGCAA
>4173523->4174707 4174687  [colik12-term-20+100.fasta] FW 44-86 16.21
GCGCACTAAAAGGGCATCAtt---------------------------TGATGCCCTTTTTGCACGCTTT
>4175322->4175867 4175847  [colik12-term-20+100.fasta] FW 49-101 16.37
TCGTTGCACAAGGCGTGAGattggaatacaat----------------TTCGCGCC-TTTTGTTTTTATG
>4182928->4187151 4187131  [colik12-term-20+100.fasta] FW 35-74 18.90
ATAACGTAAAAACCCGCTT-----------------------------CGGCGGGT-TTTTTTATGGGGG
>4187364->4187903 4187883  [colik12-term-20+100.fasta] FW 15-54 15.55
AAATAACAAAACCCACCTT-----------------------------AAGGTGGG-TTTCGCCAGAGAA
>4197859->4198131 4198111  [colik12-term-20+100.fasta] FW 40-79 14.59
GTTTTAACGAAGGGGTGGT-----------------------------TTCACCCC-TTTTGTCTTTCTG
>4211859->4212788 4212768  [colik12-term-20+100.fasta] FW 54-95 16.77
CACCTTACCTCAGGCACCTtc---------------------------GGGTGCCT-TTTTTATTTCCGA
>4225310->4226941 4226921  [colik12-term-20+100.fasta] FW 73-118 14.91
TATGAACAAGATGCCGGATcatgc------------------------ATCCGGCAATTCACTTAAAAAC
>4233485->4233727 4233707  [colik12-term-20+100.fasta] FW 81-120 14.17
ATTAACCATAACCACACTC-----------------------------CGGTGTGG-TTATTCTGCCCCT
>4235213->4237309 4237289  [colik12-term-20+100.fasta] FW 21-72 15.17
GTCATGGGAAAGGTGCCAGttttcgcactca-----------------CTGGCACC-TTCATTCTTAATG
>4250595->4251467 4251447  [colik12-term-20+100.fasta] FW 21-64 18.59
TGATGTAAAAAAGCCGGATgatc-------------------------ATCCGGCT-TTCTTCTGGGTTG
>4254694->4255302 4255282  [colik12-term-20+100.fasta] FW 22-71 15.42
ATATCTCTGAGACCGCGATgccgcctggc-------------------GTCGCGGT-TTGTTTTTCATCT
>4258178->4258885 4258865  [colik12-term-20+100.fasta] FW 52-96 16.58
TTATATAAAAGGACCCAATattt-------------------------ATTGGGTTCTTTTTTCTCTATC
>4259248->4260285 4260265  [colik12-term-20+100.fasta] FW 41-87 15.62
AAATTCACCACGCCCTGCGcaccgt-----------------------CGCGGGGCGTTTTGCTGTTAAA
>4268237->4268593 4268573  [colik12-term-20+100.fasta] FW 18-60 15.23
```

```
TGAAAGGAAAAGGCCGCTCaga---------------------------AAGCGGCC-TTAACGATTACAG
>4276058->4277407 4277387  [colik12-term-20+100.fasta] FW 31-74 17.20
GATTCTGAAAACGGGTGGCaatg--------------------------GCTGCCCG-TTTTTATTTTCTC
>4277559->4279208 4279188  [colik12-term-20+100.fasta] FW 50-91 14.11
AATCTGAAAAAGGTGCAATct----------------------------TTTGCACC-TTTAACAGGTTAG
>4366341->4366718 4366698  [colik12-term-20+100.fasta] FW 18-74 18.99
TAATGTCGAAACGCCGGATtatgtggttatgccatt-------------TTCCGGCG-TTTTTCGTTTTGG
>4368603->4370249 4370229  [colik12-term-20+100.fasta] FW 39-86 19.08
AAATAAACAAACCCCCGGGcagaaatg----------------------TCTGGGGG-TTTTTCTTTTGGT
>4400878->4401882 4401862  [colik12-term-20+100.fasta] FW 38-90 16.01
AGGTCAATAAAGCCACCGCatcctcagggatg-----------------TCGGTGGT-TTTCTTTTTCTAT
>4406853->4407584 4407564  [colik12-term-20+100.fasta] FW 29-72 18.27
AATTATGAAAAGCCATCCAgatt--------------------------TGGATGGT-TTTTTTTTGTCTA
>4423686->4424135 4424115  [colik12-term-20+100.fasta] FW 35-77 21.47
AGACGTAAAAACGCCGACCatt---------------------------GGTCGGCG-TTTTGCTTTCTAT
>4427442->4428854 4428834  [colik12-term-20+100.fasta] FW 22-70 14.02
CGCATTATCATGCTGGATGgcgcaatgc---------------------CATCCAGC-TTTTAGATCACTC
>4445471->4445812 4445792  [colik12-term-20+100.fasta] FW 21-62 14.39
CCATATGCATACGCCACCTtc----------------------------GGGTGGCG-TTGTTTTTTGCGA
>4477310->4478491 4478471  [colik12-term-20+100.fasta] FW 22-66 19.12
AGATAAAAAAAGGCCGGATgcatg-------------------------CTCCGGCC-TTGCGTTTTCATC
>4484883->4485968 4485948  [colik12-term-20+100.fasta] FW 27-66 15.01
GAACGAATAAAGGGAGCGA------------------------------AAGCTCCC-TTTATTATTGTTA
>4532583->4533599 4533579  [colik12-term-20+100.fasta] FW 67-119 16.06
ACCAATGTTAAGGCGACTTccgttttattgc------------------GAGTCGCC-TTTCCTGATTATA
>4550470->4551930 4551910  [colik12-term-20+100.fasta] FW 33-90 14.74
CCTACAGACTTACTGGTCAatcaaactgatatttggt------------TGACCAGT-TTTCGTTTTTTTG
>4552145->4552918 4552898  [colik12-term-20+100.fasta] FW 23-71 16.56
TATTATAATAAGCGCAAGGgtaaacgtt---------------------CCTTGCGC-TTTCTTAAATTAA
>4573163->4574425 4574405  [colik12-term-20+100.fasta] FW 16-69 15.83
CCTGATGATGAGCCGCTCCcgatgtggtgtcg-----------------GGAGCGGTATTTTCTATAAAAC
>4593544->4594581 4594561  [colik12-term-20+100.fasta] FW 36-89 15.33
AAAGTTGCCATGCAGCGTCcggggaagtgttg-----------------GGCGCTGTTTTTTTTGTTTCTT
>4618452->4619171 4619151  [colik12-term-20+100.fasta] FW 43-91 17.94
TGCCTTGTGAAGCCGGAGCgggagact----------------------GCTCCGGCTTTTTAGTATCTAT
>4635747->4637099 4637079  [colik12-term-20+100.fasta] FW 26-67 19.58
AAAAATAAAAACGGCGCTAaa----------------------------AAGCGCCG-TTTTTTTTGACGG
>9928<-10494 9808  [colik12-term-20+100.fasta] FW 2-43 18.27
TTGTTTTGAGATCCCCCCGct----------------------------CGGGGGGA-TTTTTTTATTCGC
>20815<-21078 20695  [colik12-term-20+100.fasta] FW 20-59 17.42
TTTGTGAAAAAGCCCGCGC------------------------------AAGCGGGT-TTTTTTATGCCTG
>59687<-60346 59567  [colik12-term-20+100.fasta] FW 43-82 19.13
TTGTACATAAATCCCCGAG------------------------------TCCGGGGA-TTTTTTGTTTCTG
>65855<-66550 65735  [colik12-term-20+100.fasta] FW 23-69 16.57
AATCAAACGAAACCAGGCTatactca-----------------------AGCCTGGT-TTTTTGATGGATT
>83622<-83708 83502  [colik12-term-20+100.fasta] FW 14-57 22.30
TCAAACAAAAAACCCGCGCcatt--------------------------GCGCGGGT-TTTTTTATGCCCG
>129407<-131260 129287  [colik12-term-20+100.fasta] FW 8-50 16.50
GACGTAAAAAAAGCGGCGTggt---------------------------TAGCCGCT-TTTTTAATTGCCG
>156299<-156883 156179  [colik12-term-20+100.fasta] FW 23-85 15.04
TGAATGAATATACAGGGAAataataatttctattttatatta-------TTCCCTGTTTTAATTAACTCTA
>217057<-218775 216937  [colik12-term-20+100.fasta] FW 1-44 16.29
ATGCCAGAAAGGGTCCTGAatt---------------------------TCAGGGCCCTTTTTTTACATGG
>220113<-220928 219993  [colik12-term-20+100.fasta] FW 28-69 14.38
ATATCATTCAGGACGGGCGct----------------------------TGCCCGTC-TTGTCATTTTTAC
>232597<-233955 232477  [colik12-term-20+100.fasta] FW 9-53 20.43
GATAATAAAAAGGCACCGAttccc-------------------------CCGGTGCC-TTTTTTATTTATG
>246242<-246502 246122  [colik12-term-20+100.fasta] FW 55-119 14.53
CTTGCACAAAAGCGTCATAaggatatgaataaattgaaatatct-----TATGACGC-TTCTTATCAATAA
>262552<-262893 262432  [colik12-term-20+100.fasta] FW 56-105 15.52
TATTGCGTAATGCGCCTGCtactacccg---------------------GCAGGCGCGTTATCTTTTTACG
>264528<-264767 264408  [colik12-term-20+100.fasta] FW 43-89 15.83
TATATCCCCATACGCCAGCcatcgcc-----------------------GCTGGCGT-TTTTATTGACGGA
>264844<-265311 264724  [colik12-term-20+100.fasta] FW 16-68 15.99
CCAGCAAATTCACCCCGACagctagcaggct------------------GCCGGGGTTTTCTTTTATCAGG
>291546<-292172 291426  [colik12-term-20+100.fasta] FW 1-44 14.08
AGTCGGTAATGGCCTTCTCagt---------------------------GAGAAGGCCTTATTTGTATTGT
>394354<-395511 394234  [colik12-term-20+100.fasta] FW 3-57 18.53
TAATACTCTGAAGGCAAACggttgatagcgacca---------------GTTTGCCT-TTATTCATTTCGA
>430353<-431237 430233  [colik12-term-20+100.fasta] FW 1-44 15.59
ATTATGAAAATGCCGGGATtta---------------------------TTCCCGGCATTTCTGATTGTTA
>446039<-446929 445919  [colik12-term-20+100.fasta] FW 9-50 16.51
TCTCTATTAAAAAGGTGCTac----------------------------GGCACCTT-TTTTCTTAGCATT
>480478<-483627 480358  [colik12-term-20+100.fasta] FW 10-49 19.51
GTAATCACTAAGGCCGCGT------------------------------AAGCGGCC-TTTTTTATGCATA
>542485<-543270 542365  [colik12-term-20+100.fasta] FW 33-77 15.19
TGTTGGATTTTGCCACGGTttat--------------------------ACCGTGGCTTTTTTTGTAACAT
```

```
>553166<-553660 553046  [colik12-term-20+100.fasta] FW 36-80 15.66
CTGCGTGGAAGAACCGGCGatcac-------------------------CGCCGGTT-TTCTGCGTTTTTT
>644340<-645803 644220  [colik12-term-20+100.fasta] FW 22-73 20.54
TAAATCATCACGCCCGCCAggtaagcccacc------------------TGGCGGGC-TTTTTTATGATTT
>656778<-657161 656658  [colik12-term-20+100.fasta] FW 4-44 18.73
ACCAAAAAAAACCCGCTGAt-----------------------------TAAGCGGG-TTTTGAATTCTTG
>658474<-659439 658354  [colik12-term-20+100.fasta] FW 26-70 19.96
TCTGATAAAAAACCCGCTTtttg--------------------------AAGCGGGTTTTTTGTATCGAAC
>661975<-663186 661855  [colik12-term-20+100.fasta] FW 49-91 14.83
CAGTAAAAAACTCCCGCCTtc----------------------------TGGCGGGAGTTGCTATTTAATT
>690129<-691007 690009  [colik12-term-20+100.fasta] FW 39-89 15.57
CATTAATTGAACGCCAGCGcattcgcctgc-------------------TGCTGGCG-TTATTATTCGGTG
>696736<-698400 696616  [colik12-term-20+100.fasta] FW 2-50 16.54
GAAATGCGAAACAGCCCCGgtgaaatc----------------------CGGGGCTGTTTCAGTTATTGAG
>709423<-709869 709303  [colik12-term-20+100.fasta] FW 10-51 17.63
GAACGAGAAAAGCCAACCTgc----------------------------GGGTTGGC-TTTTTTATGCAAG
>709423<-709869 709303  [colik12-term-20+100.fasta] FW 54-97 15.78
AAATAAGAAAAGCGAGAGTtaca--------------------------GCTCTCAC-TTATTTGTTATTA
>716169<-717488 716049  [colik12-term-20+100.fasta] FW 8-51 17.86
AAAGTAAAAAAGGGCGATTgaat--------------------------GATCGCCC-TTTCTGTTTGTTG
>720279<-720956 720159  [colik12-term-20+100.fasta] FW 28-73 16.60
TTATTAATTAAAGCCGTAAtaataa------------------------ATACGGCT-TTTTATCTTAAAC
>752408<-753691 752288  [colik12-term-20+100.fasta] FW 21-64 14.80
AAATATTTTAACCCGCCGTtcat--------------------------ATGGCGGG-TTGATTTTTATAT
>784160<-784540 784040  [colik12-term-20+100.fasta] FW 12-56 14.24
TAGCCAATAATGGGAGAGCggag--------------------------TCTCTCCCTTTTAATTTTTCAC
>786066<-786818 785946  [colik12-term-20+100.fasta] FW 14-60 20.04
TAAAATGAGAAAGCCGACTgcaagtg-----------------------AGTCGGCT-TTTTTGTTGCTAA
>847631<-848134 847511  [colik12-term-20+100.fasta] FW 32-76 16.30
CCCGATAAAAGCGGCTTCCtgaca-------------------------GGAGGCCG-TTTTGTTTTGCAG
>850237<-851820 850117  [colik12-term-20+100.fasta] FW 1-41 16.22
AAAACAAAAATCCGCCCCG------------------------------AGAGGCGGATTTTTTATATCAC
>854047<-854967 853927  [colik12-term-20+100.fasta] FW 12-54 18.13
TTAAAGCAAAAGGCGGACTcat---------------------------AATCCGCC-TTTTTTATTTGCC
>889719<-889976 889599  [colik12-term-20+100.fasta] FW 2-42 14.41
TCGTCTGACAAGCCCTCGCg-----------------------------TTGAGGGC-TTTACTGATTTTT
>914575<-915270 914455  [colik12-term-20+100.fasta] FW 2-46 17.67
TTAACGCAGAAGGCCTGGCgtaat-------------------------GTCAGGCC-TTTTTTTAAGATG
>921589<-921813 921469  [colik12-term-20+100.fasta] FW 27-71 19.45
TAAAGGCAAAATGCCAGCCcgatc-------------------------GGCTGGCA-TTTTTATCTCAAA
>925448<-925666 925328  [colik12-term-20+100.fasta] FW 33-73 16.51
AACGAGTAAAAGGTCGGTTt-----------------------------AACCGGCC-TTTTTATTTTGTG
>930308<-931273 930188  [colik12-term-20+100.fasta] FW 19-71 17.68
AAAAGTAAAGAAGGCGACAccatgcgactatg-----------------GGTCGCCT-TTATTTTTTCCCC
>949563<-950303 949443  [colik12-term-20+100.fasta] FW 3-50 19.52
TTCCCCGTAAAGCGGCAACtttattga----------------------GTTGCCGC-TTTTTTATTCCGC
>950495<-952777 950375  [colik12-term-20+100.fasta] FW 33-76 17.84
CGTACAATAAAGGCTCCACgaaa--------------------------GTGGGGCC-TTTTTTAGCGCGA
>985117<-986205 984997  [colik12-term-20+100.fasta] FW 21-63 18.34
ATGCCGAAAAAACAGGACTtt----------------------------GGTCCTGTTTTTTTTATACCTT
>986808<-988208 986688  [colik12-term-20+100.fasta] FW 5-48 19.66
AGCGTCTTCAAGAGCCAGCgccc--------------------------GCTGGCTC-TTTTTTTATCTTT
>1015175<-1015693 1015055  [colik12-term-20+100.fasta] FW 22-63 17.64
AAAAGGCGAAACCTCCGCAa-----------------------------TGCGGAGGTTTCTTTTTAAAGA
>1018236<-1019276 1018116  [colik12-term-20+100.fasta] FW 10-51 20.23
CTGGTAGAAAAACCCCGCTgc----------------------------TGCGGGGT-TTTTTTTGCCTTT
>1027627<-1027995 1027507  [colik12-term-20+100.fasta] FW 3-54 19.91
CCCTGAACAAATCCCCGGAaggtttttgcct------------------TCCGGGGA-TTTTTTATTTGGC
>1029982<-1030641 1029862  [colik12-term-20+100.fasta] FW 13-57 16.56
AACACTTTCATAGCCTCGCtttat-------------------------GCGGGGCT-TTGTTTTTTGTTA
>1041253<-1043433 1041133  [colik12-term-20+100.fasta] FW 71-117 15.67
ATCAGCCTCAGAGCATTCAggtaac------------------------TGAATGCTCTTTTTTATGCATT
>1100074<-1100907 1099954  [colik12-term-20+100.fasta] FW 41-83 15.50
TTTAAGCGAAAGCGTGAGTgcc---------------------------CCTCACGC-TTTATTTTTGATT
>1107007<-1108164 1106887  [colik12-term-20+100.fasta] FW 45-93 16.88
TCTGACCATAACCCGCTTCcagaaactg---------------------GTGGCGGG-TTCTTTTTTGAGA
>1119924<-1120178 1119804  [colik12-term-20+100.fasta] FW 15-55 15.35
TCCACCCAAAAGGCACCTCa-----------------------------CCGGTGCC-TTTACCATTTCTG
>1120465<-1120710 1120345  [colik12-term-20+100.fasta] FW 9-48 18.29
TGATTTTTTTTGCCGGGTC------------------------------GCCCCGGC-TTTTTTATTTTAC
>1140405<-1143590 1140285  [colik12-term-20+100.fasta] FW 12-55 21.07
AAAGTAATCAAGCCCTGGTaact--------------------------GCCAGGGC-TTTTTTATTTCAT
>1181006<-1182052 1180886  [colik12-term-20+100.fasta] FW 1-45 14.15
ATTCAGCAAGCGGGCGGTGgtat--------------------------TACCGCCCGTTTTTTAGTTAGC
>1196090<-1196755 1195970  [colik12-term-20+100.fasta] FW 46-85 17.00
GTATGTGAAGGGGCCGCGC------------------------------TCGCGGCC-TTTTTTACATTCC
>1201482<-1202156 1201362  [colik12-term-20+100.fasta] FW 4-43 16.75
```

```
AATGTTTATGAACCTGCTT-----------------------------CGGCAGGT-TTTTTTATACTTG
>1210903<-1211226 1210783  [colik12-term-20+100.fasta] FW 49-92 17.81
AAATAAAACAACGGGCGTGtta--------------------------TACGCCCGTTTCAATATTTAAC
>1221528<-1221863 1221408  [colik12-term-20+100.fasta] FW 4-50 17.67
TTTCATATGTACCGCGTCGatgaata----------------------CGACGCGG-TTTTTTAGCTAAC
>1232399<-1233940 1232279  [colik12-term-20+100.fasta] FW 1-45 15.84
ATAACAACTTACCGGGCATataa-------------------------ATGCCCGGTTTGCCTTTTCGCC
>1234932<-1236464 1234812  [colik12-term-20+100.fasta] FW 3-44 18.15
AACAACAAAAAACCCCTCGtt---------------------------TGAGGGGT-TTGCTCTTTAAAC
>1255944<-1257035 1255824  [colik12-term-20+100.fasta] FW 51-92 20.92
AGTATATAAAATCCACGCAat---------------------------TGCGTGGA-TTTTTCATTTCAC
>1260151<-1261098 1260031  [colik12-term-20+100.fasta] FW 8-47 17.16
CCGGCTCAAAGACCCGCTG-----------------------------CGGCGGGT-TTTTTTGTCTGTA
>1291732<-1292145 1291612  [colik12-term-20+100.fasta] FW 18-58 19.00
CTTGCTTAAAATCCCGCCAg----------------------------CGGCGGGA-TTTTTTATTGTCC
>1304845<-1305252 1304725  [colik12-term-20+100.fasta] FW 14-57 19.03
AGCGTGAAAAAGGGCTGACaact-------------------------GTCAGCCC-TTATTGTTTTTAA
>1312742<-1313248 1312622  [colik12-term-20+100.fasta] FW 15-60 14.60
ATGTCCTTATTGACCCCGTatatt------------------------ACGGGGTCGTTTTTGTGCGGAA
>1345002<-1346936 1344882  [colik12-term-20+100.fasta] FW 2-49 14.16
TCTCCTTTCACGGCCCATTcctcatg----------------------GATGGGCCGTTTATTTCCCCGC
>1348275<-1349063 1348155  [colik12-term-20+100.fasta] FW 10-53 15.20
TTGGTAAAGATGGGCGGCGttc--------------------------TGCCGCCCGTTATCTCTGTTAT
>1435284<-1438808 1435164  [colik12-term-20+100.fasta] FW 28-71 19.04
CTAAAAAAAAAGCCCGGACgact-------------------------GTTCGGGC-TTGTCTTTTTATA
>1447100<-1449373 1446980  [colik12-term-20+100.fasta] FW 8-48 19.83
CAGACAAAAAAACGGCACCa----------------------------GGTGCCGT-TTTTTACTTATGA
>1449621<-1451666 1449501  [colik12-term-20+100.fasta] FW 2-49 16.15
TCGGTGAATGAAGGGCAACggcgaata---------------------GTTGCCCT-TTTATTTCACTAA
>1518987<-1521089 1518867  [colik12-term-20+100.fasta] FW 1-43 15.09
ATTACCTTGATGCCCGGTAtt---------------------------TGCCGGGCATTTACTTTAACGA
>1553850<-1553987 1553730  [colik12-term-20+100.fasta] FW 22-69 15.89
ATGACGATTAAACACCATTgcctgcgc---------------------AATGGTGT-TTTTGTTTTTATC
>1565528<-1566847 1565408  [colik12-term-20+100.fasta] FW 13-62 14.57
TACTTTGTAACAAGCCGGTgctttaccc--------------------ACCGGCTTGTTACCCTCTTAAA
>1566978<-1568513 1566858  [colik12-term-20+100.fasta] FW 16-62 18.63
TTATAAGACAAGGGAGCGAtaattca----------------------TCGCTCCC-TTTTTCGTGCTTG
>1573271<-1575643 1573151  [colik12-term-20+100.fasta] FW 9-52 14.21
GATTTTACTTAACCGGGTAaatt-------------------------CGCCCGGT-TTTCGCATGGAGA
>1577657<-1578829 1577537  [colik12-term-20+100.fasta] FW 44-93 14.65
CCTTAACAAGGCACCCGACataaattcct-------------------GTCGGGTG-TTTCGTCATCATC
>1578866<-1580581 1578746  [colik12-term-20+100.fasta] FW 3-47 15.75
TAAACCTTCATGCGGCGGAtttt-------------------------TCCGCCGCCTTATTGAGCGAGA
>1590689<-1592089 1590569  [colik12-term-20+100.fasta] FW 1-48 14.38
AACCGTATAAGCCGCATGTcgagatg----------------------GCATGCGGCTTAATATTGCCGA
>1607253<-1608704 1607133  [colik12-term-20+100.fasta] FW 10-49 16.52
TTACTTCTGAACCCGGTTT-----------------------------CGACCGGG-TTTCTTATTTCAC
>1643143<-1643298 1643023  [colik12-term-20+100.fasta] FW 60-99 16.29
ACCCGCACTTAACCCGCTT-----------------------------CGGCGGGT-TTTTGTTTTTATT
>1734145<-1735314 1734025  [colik12-term-20+100.fasta] FW 2-47 17.26
TTAAACAAAAACGCAGAAGgataac-----------------------CTTCTGCG-TTACAGTTGCTGA
>1755745<-1756749 1755625  [colik12-term-20+100.fasta] FW 14-56 19.90
GACAAAAAAAATGGCGCACaat--------------------------GTGCGCCA-TTTTTCACTTCAC
>1790291<-1790755 1790171  [colik12-term-20+100.fasta] FW 3-53 16.75
ATGTCGAAAAATGGACGGCgatgatacatc------------------GCCGTCCA-TTTACTCCTGAAT
>1793277<-1793576 1793157  [colik12-term-20+100.fasta] FW 7-47 21.84
TCTAACTAAAAAGGCCGCTc----------------------------TGCGGCCT-TTTTTCTTTTCAC
>1797250<-1797294 1797130  [colik12-term-20+100.fasta] FW 39-80 14.64
CAGCGCCTGAAAGCCTCCCag---------------------------TGGAGGCT-TTTTTTGTATGCG
>1797417<-1797773 1797297  [colik12-term-20+100.fasta] FW 2-44 15.24
GCCAGTTGAAAGAGGGAGCtag--------------------------TCTCCCTC-TTTTCGTTTCAAC
>1819942<-1820280 1819822  [colik12-term-20+100.fasta] FW 23-66 14.41
GTAACAACGGAAACCGGCCattg-------------------------CGCCGGTT-TTTTTTGGCCTGA
>1843023<-1844984 1842903  [colik12-term-20+100.fasta] FW 31-85 15.78
ATATTCTGAAATATCCAGCggatcaagaaaattc--------------GTTGGATA-TTTTTTTTGCATG
>1852120<-1852878 1852000  [colik12-term-20+100.fasta] FW 52-98 14.91
GATGATAAAGATGCGGCAAgtgaat-----------------------TTGCCGCAATTGCTACCTTTTT
>1863750<-1864496 1863630  [colik12-term-20+100.fasta] FW 26-66 17.69
GTGCATAAAAACAGGGCGTc----------------------------ACGCCCTG-TTTTGCATTACGG
>1877031<-1877279 1876911  [colik12-term-20+100.fasta] FW 7-50 14.95
AAATTGCACAAAGGCTGCAcaca-------------------------GGCAGCCT-TTGCTATTTTTTA
>1886085<-1887770 1885965  [colik12-term-20+100.fasta] FW 10-52 15.92
TCAGTCGTCAGACGCCGGTtaa--------------------------TCCGGCGT-TTTTTTTGACGCC
>1887975<-1888556 1887855  [colik12-term-20+100.fasta] FW 15-57 17.08
AAGAAAAAGAAACAGCGGCtgg--------------------------TCCGCTGT-TTCTGCATTCTTA
>1905250<-1905459 1905130  [colik12-term-20+100.fasta] FW 38-83 17.55
TCTCGCTATAAAGCCTCGTcgaat------------------------GCGAGGCTTTTTACTATGCTTT
```

```
>1907332<-1908123 1907212  [colik12-term-20+100.fasta] FW 1-54 15.09
ATGAGAGTAAGAACCTGTCggaatatcaaaca-----------------GACAGGTTCTTTATTTAGCATG
>1909719<-1910600 1909599  [colik12-term-20+100.fasta] FW 10-55 22.21
AAATACTAAAAAGCGCGTCctgatc------------------------GACGCGCT-TTTTTTATGCCCT
>1921389<-1921730 1921269  [colik12-term-20+100.fasta] FW 25-82 18.73
TCCAATAAAAAAACCGCCTcagttctttcaccagaac------------GGGCGGTT-TTTAACATTTCAG
>1930139<-1930780 1930019  [colik12-term-20+100.fasta] FW 1-44 16.61
AAAGTCAAAATGCCCGATCgag---------------------------GATCGGGCATTTTTGTAGCGTT
>1932863<-1934338 1932743  [colik12-term-20+100.fasta] FW 70-120 18.35
TACACTTTCAGGCCTCGTGcggattcacc--------------------CACGAGGCTTTTTTTATTACAC
>1938337<-1939596 1938217  [colik12-term-20+100.fasta] FW 6-54 18.35
CATCCATTCGCAGCCGGTAcgcagtcag---------------------TACCGGCT-TTTTTTATTTGGT
>1956544<-1956984 1956424  [colik12-term-20+100.fasta] FW 39-86 17.34
GCTCGTACCAGGCCCCTGCaatttca-----------------------ACAGGGGCCTTTTTTTATCCCT
>1970860<-1971363 1970740  [colik12-term-20+100.fasta] FW 19-63 17.64
ATTGAAATGAACCCGATGAtctgc-------------------------GCATCGGG-TTTTTTATTTCAA
>1975290<-1975868 1975170  [colik12-term-20+100.fasta] FW 18-64 16.17
CAACATTCCAGCAGCGGTAacgacg------------------------TACCGCTGCTTTTTTTTGCCCC
>1980578<-1980838 1980458  [colik12-term-20+100.fasta] FW 30-78 14.43
TTTCCCACCATAGCCAACCgccataac----------------------GGTTGGCTGTTCTTCGTTGCAA
>1994133<-1994855 1994013  [colik12-term-20+100.fasta] FW 24-74 20.02
AGATGCAAAAACCGGCTGAaaggcacgcta-------------------TCAGCCGG-TTTTATATTACTG
>1997608<-1998408 1997488  [colik12-term-20+100.fasta] FW 10-53 16.67
ATGACAAAAAAGGGCGCTTtcac--------------------------TAGCGCCT-TTTTTATTTACGC
>2060413<-2061345 2060293  [colik12-term-20+100.fasta] FW 16-60 18.36
AGAAAGCAAAAAGCCTGCTagaa--------------------------AGCAGGCTTTTTTGAATTTGGC
>2077054<-2077449 2076934  [colik12-term-20+100.fasta] FW 7-53 14.43
GTTTTATTCAATGAGGGTTgcccggc-----------------------AACCCTCA-TTGCTCATTGATT
>2083726<-2085090 2083606  [colik12-term-20+100.fasta] FW 10-63 21.51
TCTAATAATAAACCGGAGTcgaatgaacatttg----------------GCTCCGGT-TTTTTTATTCTTT
>2095343<-2096359 2095223  [colik12-term-20+100.fasta] FW 13-61 17.24
TTAAAGAAAAAGGGCAGGGtggtgaca----------------------CCTTGCCCGTTTTTTTGCCGGA
>2097884<-2099290 2097764  [colik12-term-20+100.fasta] FW 18-64 17.27
ATAAAATTGAGGCCCGGCGtatattg-----------------------CACCGGGC-TTTTTTTTGCCAA
>2165324<-2165542 2165204  [colik12-term-20+100.fasta] FW 4-45 17.21
AGAAACAGGAAGCCCCTCAgt----------------------------CGAGGGGC-TTTTTTGTCGATG
>2185400<-2186434 2185280  [colik12-term-20+100.fasta] FW 47-90 16.62
TGTCTGCATTTCCCCCTCAtga---------------------------TGAGGGGGCTTTTTTTAGCGAT
>2189700<-2190242 2189580  [colik12-term-20+100.fasta] FW 27-71 15.94
GTAATAAACATGGCCGCTAtccca-------------------------TGGCGGCC-TTTTAATTTAAGA
>2190535<-2190816 2190415  [colik12-term-20+100.fasta] FW 23-76 18.28
GCATTTAGAAAGCCGAAATcatttatataaatg----------------ATTTCGGC-TTTTTTATTGATA
>2245083<-2246552 2244963  [colik12-term-20+100.fasta] FW 12-55 17.88
TTCCTTGCTAAGCCCTCTCaacc--------------------------GAGAGGGC-TTTTTCAATTCCA
>2255449<-2256387 2255329  [colik12-term-20+100.fasta] FW 40-81 14.52
GCATACCAAAACCTGGCGCtg----------------------------ACGCCGGG-TTTACCGGCATGA
>2284410<-2286920 2284290  [colik12-term-20+100.fasta] FW 57-101 18.67
AGATATAAAAAAACCAACCgtaag-------------------------GGTTGGTT-TTTTCTTGGGATT
>2309666<-2310769 2309546  [colik12-term-20+100.fasta] FW 11-50 19.00
ATATCGAACAAAGGGCCTG------------------------------CGGGCCCT-TTTTTCATTGTTT
>2325387<-2326163 2325267  [colik12-term-20+100.fasta] FW 28-77 18.04
GTTCAAGAAAACACCCGATaactttcgct--------------------ATCGGGTG-TTTTTATTGATTA
>2388068<-2389345 2387948  [colik12-term-20+100.fasta] FW 15-57 15.89
AAGTCAGAAAGCCGCCGACatg---------------------------CTCGGCGG-TTTTTCTGAAAAA
>2403723<-2404661 2403603  [colik12-term-20+100.fasta] FW 36-89 19.84
GTAAAAAACGAGCCGCTGGgggtgttttaaacc----------------CCAGCGGC-TTTTTTTTAGTAA
>2409459<-2410127 2409339  [colik12-term-20+100.fasta] FW 10-51 17.46
ATCATGATTTAGCCCCGTCtc----------------------------GTCGGGGC-TTTTTTATGGCAG
>2410697<-2411152 2410577  [colik12-term-20+100.fasta] FW 15-59 15.35
ACGCATATAAAAGAAGGCAtactc-------------------------AGCCTTCT-TTTTTTTCACCGT
>2431032<-2431946 2430912  [colik12-term-20+100.fasta] FW 1-47 17.30
ATAACTGATAAGGGCAGGGccactgg-----------------------CTCTGCCC-TTTTGCTATTCTC
>2438405<-2439625 2438285  [colik12-term-20+100.fasta] FW 70-111 16.84
CTAACGCAAAAGGGAACCTga----------------------------TGGTTCCC-TTTTTCACATCAT
>2458670<-2458978 2458550  [colik12-term-20+100.fasta] FW 8-58 16.18
TCGGGTACGTACGCCCCTGtcagttgctgg-------------------CAGGGGCG-TTTTCTTTTCTCT
>2462272<-2463027 2462152  [colik12-term-20+100.fasta] FW 5-50 18.08
ACAAATAAAAAAGGTGAGTcgcaaa------------------------ACTCACCT-TTTTTGTTGTTAT
>2487262<-2488206 2487142  [colik12-term-20+100.fasta] FW 19-68 14.60
TTTACATAAATGCCGAAGTgtcttttca---------------------CCTTCGGCTTTTTTCAAGGCAT
>2495077<-2496315 2494957  [colik12-term-20+100.fasta] FW 13-56 18.82
AGAAATGCAAACAGGAGCCtgat--------------------------GGCTCCTG-TTTTTCATTGCAC
>2517277<-2518692 2517157  [colik12-term-20+100.fasta] FW 11-57 21.20
AAAAAGATAAACGGCAGGAgataata-----------------------TCCTGCCG-TTTTTTATTTATG
>2519613<-2520497 2519493  [colik12-term-20+100.fasta] FW 73-114 18.81
GATAGCAAAAAAGCGCCTTt----------------------------AGGGCGCTTTTTTTACATTGGTG
>2528267<-2529253 2528147  [colik12-term-20+100.fasta] FW 21-64 16.86
```

```
CTCCTCTTCGAACCCCCGCttg---------------------------TCGGGGGTTTTTAGCATTGATG
>2537737<-2538834 2537617  [colik12-term-20+100.fasta] FW 12-63 15.69
GAATGTTAAACGCCCGGAGgcgcttcccgcg------------------ATCCGGGC-TTTTTAATGGCAA
>2548661<-2549236 2548541  [colik12-term-20+100.fasta] FW 46-88 14.23
GCGCTCAACAATGCCACGGatt---------------------------GCGTGGCA-TTATTATTTTCAG
>2618266<-2618919 2618146  [colik12-term-20+100.fasta] FW 8-52 20.13
AAAATAATTAAAGCCGACTttaag-------------------------AGTCGGCT-TTTTTTTGAGTAA
>2628978<-2630555 2628858  [colik12-term-20+100.fasta] FW 19-62 18.87
AATGAACAAAACCCTCTGTtact--------------------------ACAGAGGG-TTTTTTATCTTCA
>2635494<-2636672 2635374  [colik12-term-20+100.fasta] FW 16-60 17.01
CACTTTGAAAACGGCTCCTggac--------------------------AGGGGCCGTTTTCCTGTTTTTA
>2642453<-2642884 2642333  [colik12-term-20+100.fasta] FW 51-107 14.41
GTACAATGCAGCGCCCCCGgacgagcagccgctca--------------CCGGGGCGTTTCTTTTTTCAAC
>2657583<-2657906 2657463  [colik12-term-20+100.fasta] FW 5-57 14.06
GCATACCGATAACCCCACCgtggtcgcctgcg-----------------CGTGGGGT-TTGTTTTACCTGA
>2689676<-2693563 2689556  [colik12-term-20+100.fasta] FW 49-94 15.69
ACCACTATCATAAGCTCCCaactcc------------------------GGGAGCTT-TTTTGTGTCTGTA
>2698638<-2699018 2698518  [colik12-term-20+100.fasta] FW 20-70 19.63
TAAAAAGTAAAACCCCCGAactggtaagga-------------------ACGGGGGT-TTTACTTTTTAAC
>2706774<-2707424 2706654  [colik12-term-20+100.fasta] FW 49-100 15.65
TTCTCTGCTAACGCCTCGGccactcccgcgt------------------CCGGGGCG-TTATTACAGCAGA
>2714086<-2714469 2713966  [colik12-term-20+100.fasta] FW 13-60 18.17
CAAGTTACTAAGCGGAAGAgggcgacc----------------------TCTTCCGC-TTTTTTTCGTTAA
>2729620<-2732193 2729500  [colik12-term-20+100.fasta] FW 1-41 15.09
AATGATAAAACGAGCCCTT------------------------------CGGGGCTCGTTTTTGTCTATAA
>2742203<-2742550 2742083  [colik12-term-20+100.fasta] FW 21-66 18.26
CCTGTTAAGAAGGGCTGGCcaattg------------------------GCTGGCCC-TTTTTTATCTGTT
>2748136<-2748729 2748016  [colik12-term-20+100.fasta] FW 12-63 15.81
CGTAATAATTCACGGCCCTgcatgcgaatgc------------------CGGGCCGT-TTTCGTTACTCCG
>2752029<-2752337 2751909  [colik12-term-20+100.fasta] FW 4-44 17.80
GACAGAAAAAAGGTGCTCAt-----------------------------TGAGCACC-TTTTTTAACGTCT
>2776167<-2780876 2776047  [colik12-term-20+100.fasta] FW 4-45 18.21
ATCAACGAAAAAGCCCACAtc----------------------------TGTGGGCT-TTCATGTTACCAG
>2820731<-2821792 2820611  [colik12-term-20+100.fasta] FW 11-52 17.94
TTGATACACAAGGGTCGCAtc----------------------------TGCGGCCC-TTTTGCTTTTTTA
>2864582<-2865574 2864462  [colik12-term-20+100.fasta] FW 9-56 17.35
TCTGTCAGAAAGGCCAGTCtcaagcga----------------------GGCTGGCC-TTTTCTGTGCACA
>2885601<-2886335 2885481  [colik12-term-20+100.fasta] FW 11-61 15.97
GACAAATTAAACGCCTCTGtcagaaatga--------------------TGGGGGCGTTTTTATTTTGCGA
>2904665<-2905963 2904545  [colik12-term-20+100.fasta] FW 15-58 21.25
TGATTTAAAAATGCCAGCCtccg--------------------------GGCTGGCA-TTTTTTATGGTTG
>2906051<-2907688 2905931  [colik12-term-20+100.fasta] FW 12-54 14.82
TAGAGCGGCAACGCGTACCctg---------------------------GGTACGCG-TTGTTTGTCTGGA
>2962383<-2963177 2962263  [colik12-term-20+100.fasta] FW 12-55 16.21
TCCTGCCAGAGCCGACGCCagtg--------------------------TGCGTCGG-TTTTTTTACCCTC
>2980519<-2981280 2980399  [colik12-term-20+100.fasta] FW 13-54 17.50
TACTTTTATGACCCTGCCGca----------------------------TGGCAGGG-TTTTTTATACCTG
>3031677<-3033194 3031557  [colik12-term-20+100.fasta] FW 11-53 19.19
TATGCTCACAACCCCGGCAaa----------------------------TGTCGGGGTTTTTTTATTTAAG
>3044188<-3047061 3044068  [colik12-term-20+100.fasta] FW 15-54 16.98
CTATTTTCTAAAGGCGCTT------------------------------CGGCGCCT-TTTTAGTCAGATG
>3055198<-3056430 3055078  [colik12-term-20+100.fasta] FW 12-59 19.01
CTGAAAATCAACGGGCAGGtcactga-----------------------CTTGCCCGTTTTTTTATCCCTT
>3081954<-3083930 3081834  [colik12-term-20+100.fasta] FW 60-117 17.41
GACAAATCCAACCCTTCCTcgtcgggcctaacgacg-------------CGGAAGGGTTTTTTTATATCGA
>3098923<-3099642 3098803  [colik12-term-20+100.fasta] FW 5-53 16.26
TCTATTTTAAGACGGCATAatactttt----------------------TATGCCGT-TTAATTCTTCGTT
>3105038<-3107233 3104918  [colik12-term-20+100.fasta] FW 3-48 19.49
AATAAAAAAAACGGGTCACcttctg------------------------GCGACCCG-TTTTTCTTTGCGT
>3117613<-3119295 3117493  [colik12-term-20+100.fasta] FW 1-50 15.82
AATCAATAAAGCCGGATGAtcgttaata---------------------TCATCCGGCTTCATTTACATTA
>3129356<-3130333 3129236  [colik12-term-20+100.fasta] FW 21-85 15.61
GGAAATTCTTCTCGGCTGActcagtcatttcatttcttcatgtt-----TGAGCCGA-TTTTTTCTCCCGT
>3132887<-3134386 3132767  [colik12-term-20+100.fasta] FW 3-49 16.10
CTTCCTGAAAATGCCCGGTcctggcg-----------------------ATCGGGCA-TTTCCATTTTTGA
>3181829<-3182482 3181709  [colik12-term-20+100.fasta] FW 25-72 18.18
TTAATCAAGAAACCGAAGTtgtagcag----------------------GCTTCGGT-TTTTATTTTTCCC
>3213368<-3214132 3213248  [colik12-term-20+100.fasta] FW 10-57 17.92
TAAAAAGAAAAAGGCTGACgatttctc----------------------GTCAGCCT-TTGCTATATCTGG
>3239467<-3240954 3239347  [colik12-term-20+100.fasta] FW 16-66 18.43
GTTAATAAAAAACGGCGTTtcataatgtga-------------------AGCGCCGT-TTGCTTTTCCATA
>3255928<-3256755 3255808  [colik12-term-20+100.fasta] FW 15-56 15.22
TTTCATCCTTGGGTGCACAaa----------------------------TGTGCACC-TTTTTTTTGTGAT
>3303612<-3305552 3303492  [colik12-term-20+100.fasta] FW 33-81 18.53
ATAATATATACAGCCCCGAttttacca----------------------TCGGGGCT-TTTTTTCTGTCTT
>3309056<-3309325 3308936  [colik12-term-20+100.fasta] FW 11-53 16.46
GTTTCAGAAAAGGGGGCCTgag---------------------------TGGCCCCT-TTTTTCAAGCTGA
```

```
>3320374<-3321711 3320254  [colik12-term-20+100.fasta] FW 13-54 16.93
ACTGGCTAAAAAGGCGGCGat----------------------------TGTCGCCT-TTTTTCTCAGCGA
>3322642<-3324576 3322522  [colik12-term-20+100.fasta] FW 25-70 19.50
TTGTACCGAAAACCCCGGGgcgtgc------------------------TCCGGGGT-TTTTTCTTATCAA
>3325880<-3326341 3325760  [colik12-term-20+100.fasta] FW 38-78 17.34
AGAAAGGAAAAAGGCCGCTa-----------------------------TGCGGCCT-TTTATCAACGAAC
>3330503<-3330760 3330383  [colik12-term-20+100.fasta] FW 18-65 19.40
TAACGGATGAAAGCCCCGCaacacgtt----------------------GCGGGGCT-TTTTACATTCGAT
>3375450<-3375842 3375330  [colik12-term-20+100.fasta] FW 28-67 19.12
TTTTCGAAAAAACCCGCTT------------------------------CGGCGGGT-TTTTTTATAGCTA
>3380965<-3381903 3380845  [colik12-term-20+100.fasta] FW 12-55 18.92
GATAATAAAAAACCGGAGCacag--------------------------ACTCCGGT-TTTTTGTTTTGAG
>3437253<-3437636 3437133  [colik12-term-20+100.fasta] FW 10-49 20.06
AACGTAAAAAAACCCGCCC------------------------------CGGCGGGT-TTTTTTATACCCG
>3445951<-3446205 3445831  [colik12-term-20+100.fasta] FW 18-61 20.02
AATACGAATAAACGGCTCAgaaa--------------------------TGAGCCGT-TTATTTTTTCTAC
>3464797<-3467490 3464677  [colik12-term-20+100.fasta] FW 34-78 16.88
TTTTTTGTCAATGGGCTACttttg-------------------------GTAGCCCA-TTATTTTTATTCA
>3467782<-3468966 3467662  [colik12-term-20+100.fasta] FW 19-60 18.47
TTGAATTGAAAAGGGCGCTtc----------------------------GGCGCCCT-TTTTGCATTTGTT
>3472315<-3472602 3472195  [colik12-term-20+100.fasta] FW 46-92 15.14
CTAAAATTCGGCGTCCTCAtattgtg-----------------------TGAGGACG-TTTTATTACGTGT
>3474244<-3475056 3474124  [colik12-term-20+100.fasta] FW 1-47 21.37
AGCATTAAGAACCGCCGCCtgacca------------------------GGCGGCGGTTTTTTTATTACAG
>3475544<-3476134 3475424  [colik12-term-20+100.fasta] FW 11-51 21.67
AGTGACAAAAAAGCGGGGAa-----------------------------TCCCCGCT-TTTTTTACGCCTC
>3561767<-3564214 3561647  [colik12-term-20+100.fasta] FW 8-51 17.78
CAATAAATAGAACGGGGCCaaag--------------------------GGTCCCGT-TTTTTTCCGCCAT
>3574697<-3575185 3574577  [colik12-term-20+100.fasta] FW 19-72 14.26
GCTTGTTTTAACAGCAGTAgggtctgttttac-----------------TGCTGCTGTTTTTAGTCATGAA
>3582712<-3584454 3582592  [colik12-term-20+100.fasta] FW 18-73 15.06
CGTGGGAAGAGGGCTATTTtgtcagggcaagccga--------------AGGTAGCC-TTTTTTATTTCGT
>3597560<-3598414 3597440  [colik12-term-20+100.fasta] FW 12-55 16.91
TAAGCAGAGAACCCTGGATgaga--------------------------GTCCGGGG-TTTTTGTTTTTTG
>3653596<-3653934 3653476  [colik12-term-20+100.fasta] FW 8-63 17.37
GCAAGTAAAAAGGAGTAGCaagttgagccatctt---------------GCTGCTCCTTTTTGCATTTTTA
>3663810<-3665210 3663690  [colik12-term-20+100.fasta] FW 10-72 16.51
CGTTGTAAAAACCGAATGCccagcctttaaaaaaacagctgg-------GCATTCGG-TTGCTTATTAATC
>3665421<-3666818 3665301  [colik12-term-20+100.fasta] FW 1-49 14.52
ATTAATTTGATCGCCCGAAcagcaatg----------------------TTTGGGCGATTTTTATTACGAT
>3708428<-3709636 3708308  [colik12-term-20+100.fasta] FW 75-116 16.67
TCGTAAAATTTGGGCCAATta----------------------------ACTGGCCC-TTTTTACTGCTTT
>3719957<-3722026 3719837  [colik12-term-20+100.fasta] FW 8-51 18.59
CGTTATTAAATAGCCTGCCatct--------------------------GGCAGGCT-TTTTTTATCGCTA
>3724553<-3724993 3724433  [colik12-term-20+100.fasta] FW 1-49 17.15
ACTCACGTAAGCCCGGTCAgtccaatg----------------------TGACCGGGCTTTTACTTAACTC
>3733982<-3734806 3733862  [colik12-term-20+100.fasta] FW 5-58 14.83
CATTTCCTTGAGCCTTATCcgacttgtcagtcg----------------GATAAGGC-TTTTTACTTTGTC
>3755644<-3757488 3755524  [colik12-term-20+100.fasta] FW 50-102 17.26
AATTTAAAAAAAGCACATTgtttaatgaatac-----------------AATGTGCT-TTTTATTAGATTA
>3790453<-3791325 3790333  [colik12-term-20+100.fasta] FW 42-81 16.38
GATAGGAAAAATGATGCTT------------------------------TGGCATCA-TTTTTTCTTTTTA
>3808877<-3809044 3808757  [colik12-term-20+100.fasta] FW 14-55 18.75
TGTAACAAAAAACCTCGCTcc----------------------------GGCGGGGT-TTTTTGTTATCTG
>3813490<-3814176 3813370  [colik12-term-20+100.fasta] FW 26-69 17.58
ACTGATTTTTAAGGCGACTgatg--------------------------AGTCGCCT-TTTTTTTGTCTGT
>3843403<-3844794 3843283  [colik12-term-20+100.fasta] FW 10-51 16.54
GGTGACTTTTGCCCGGCATg-----------------------------ACGCCGGGCTTTTTTTATTATT
>3850517<-3850615 3850397  [colik12-term-20+100.fasta] FW 18-66 18.99
CGATTCCAAAACCCCGCCGgcgcaaacc---------------------GGGCGGGG-TTTTTCGTTTAAG
>3864096<-3864530 3863976  [colik12-term-20+100.fasta] FW 6-50 19.00
CTATTCTCTTTGCCCCGCCattct-------------------------GGTGGGGC-TTTTTTTGTCTTA
>3868065<-3869402 3867945  [colik12-term-20+100.fasta] FW 44-91 16.26
CACTTTCTTAAAGCCGGTGcggtcagg----------------------CATCGGCT-TTTTACTATCTTG
>3892901<-3894238 3892781  [colik12-term-20+100.fasta] FW 11-59 20.62
CGCGTTAAAAACCCGCCATgcatgattg---------------------CTGGCGGG-TTTTTTATTTAGA
>3896311<-3896952 3896191  [colik12-term-20+100.fasta] FW 14-68 14.74
CTTAAGAAATAAGGCCGGAtgtgtgaattacgc----------------TCCGGCCTGTTCTCATTATTTA
>3903359<-3904195 3903239  [colik12-term-20+100.fasta] FW 38-105 16.50
AGCGGGCAAAACCTGAAAAaaattgcttgattcacgtcaggccgttt--TTTTCAGG-TTTTTTTTTGGAG
>3913181<-3913600 3913061  [colik12-term-20+100.fasta] FW 12-59 17.54
AAAAGCACAAAAGCCAGTCtggaaaca----------------------GGCTGGCT-TTTTTTTGCGCGT
>3984299<-3985495 3984179  [colik12-term-20+100.fasta] FW 45-94 15.54
ACATAAAAAAACGCCTGCTcttattacgg--------------------AGCAGGCG-TTAAAACAGGTCT
>4001841<-4002326 4001721  [colik12-term-20+100.fasta] FW 59-112 15.75
GCAAAACAAACGCGGCAGGcgtattactcgct-----------------CTTGCCGC-TTATTTTATTTGG
>4038486<-4038998 4038366  [colik12-term-20+100.fasta] FW 58-101 17.29
```

```
CAATGCAAAAAGGCCATCCgtca---------------------------GGATGGCC-TTCTGCTTAATTT
>4078805<-4079440 4078685  [colik12-term-20+100.fasta] FW 39-82 14.53
CGAGAAACGTACGGCGGATatga---------------------------TTCCGCCG-TTATTGTTCCCTC
>4124866<-4125474 4124746  [colik12-term-20+100.fasta] FW 17-56 18.23
GGCACAAAAAAAGCGCCGT-------------------------------GCGGCGCT-TTTTTCGGAAATC
>4148026<-4150677 4147906  [colik12-term-20+100.fasta] FW 8-53 14.39
TCTTCTGCAAACCCTCGTGcttttg-------------------------CGCGAGGG-TTTTCTGAAATAC
>4188313<-4189446 4188193  [colik12-term-20+100.fasta] FW 42-91 14.65
GAAGATGAAAAACGCAAGGttgttgaaag---------------------CGTTGTGT-TTTTTTATGGTAG
>4202220<-4203509 4202100  [colik12-term-20+100.fasta] FW 36-87 17.09
TCCCTAACTGACGGGCGGCttcggttttgtt-------------------GCCGCCCG-TTTTCTCCAGCGC
>4240205<-4241095 4240085  [colik12-term-20+100.fasta] FW 61-103 18.24
GACTGTTATTCGGCGCTCCac-----------------------------GGAGCGCCTTTTTTTCTTTCGT
>4294798<-4296945 4294678  [colik12-term-20+100.fasta] FW 5-48 17.99
CGTCCTTTCTACAGCCTCCtttc---------------------------GGAGGCTG-TTTTTTTATCCAT
>4333272<-4334609 4333152  [colik12-term-20+100.fasta] FW 17-66 17.17
CAAACATAAAAGCCAACCTtaagaactta---------------------AGGTTGGC-TTAATTTTGCTTT
>4350778<-4352295 4350658  [colik12-term-20+100.fasta] FW 7-54 14.82
ACTTTAATGAACGAAGCAGtcaggcga-----------------------CTGCTTCG-TTCATCGTTCCGC
>4354048<-4356195 4353928  [colik12-term-20+100.fasta] FW 4-49 14.22
AGCTCGTACAAGGGAAGTGgcttgc-------------------------CACTTCCC-TTTTTTGCTCATA
>4356275<-4357609 4356155  [colik12-term-20+100.fasta] FW 8-71 15.88
GTATTTTCCGAGGCTCCTCctttcattttgtcccatgtgttgg------GAGGGGCC-TTTTTTACCTGGA
>4364469<-4365950 4364349  [colik12-term-20+100.fasta] FW 17-60 20.95
CAAATAAAAAAGGCACGTCagat---------------------------GACGTGCC-TTTTTTCTTGTGA
>4376585<-4376944 4376465  [colik12-term-20+100.fasta] FW 10-54 18.55
CAATGTAAATCCGGCCCGCctatg--------------------------GCGGGCCG-TTTTGTATGGAAA
>4422094<-4422369 4421974  [colik12-term-20+100.fasta] FW 31-72 17.04
TAGTTTGCTTTGCCCCCTTcg-----------------------------CAGGGGGC-TTTTTTTTGAAAA
>4424206<-4425000 4424086  [colik12-term-20+100.fasta] FW 17-60 19.91
AGAAAGCAAAACGCCGACCaat----------------------------GGTCGGCGTTTTTACGTCTCGT
>4436285<-4436839 4436165  [colik12-term-20+100.fasta] FW 23-76 14.59
AAAAATAATTTTCCCCTATcgttatctatggg------------------ATAGGGGAATTAAGTCTCTGAG
>4460628<-4462283 4460508  [colik12-term-20+100.fasta] FW 3-52 15.49
TCTTCCCTAAGCCCCGGTAatgccggtca---------------------TTCCGGGG-TTTTGTCTGTCAG
>4468101<-4468526 4467981  [colik12-term-20+100.fasta] FW 3-46 15.70
CTTGATGGAAATCCGGGCTatca---------------------------TGCCCGGA-TTAAGTCTGATGA
>4468560<-4469021 4468440  [colik12-term-20+100.fasta] FW 10-58 17.29
GGTAATAAAAGTCTGGCTCcctataat-----------------------GAGCCAGACTTTTTACCGCTGT
>4478550<-4481405 4478430  [colik12-term-20+100.fasta] FW 16-61 18.31
TGAAAACGCAAGGCCGGAGcatgc--------------------------ATCCGGCCTTTTTTTATCTCTT
>4577638<-4579032 4577518  [colik12-term-20+100.fasta] FW 4-46 15.76
ATTATTTTCTGGCGCACCTttc----------------------------CGGTGCGC-TTTTTATTATTTC
>4637159<-4637875 4637039  [colik12-term-20+100.fasta] FW 15-58 20.55
CGTCAAAAAAAACGGCGCTtttt---------------------------AGCGCCGT-TTTTATTTTTCAA
>4866->6782 6762  [bsub-term-20+100.fasta] FW 18-69 14.62
TAATCATAAAAAGCCTTATttccaataagaa-------------------ATAAGGCT-TTTTTCTGAACAA
>6993->9458 9438  [bsub-term-20+100.fasta] FW 15-61 19.95
GTGTGAAAAAAAGCGCAGCtgaaata------------------------GCTGCGCT-TTTTTGTGTCATA
>20878->22155 22135  [bsub-term-20+100.fasta] FW 20-66 17.55
AATTATGGAAAGGCGTGCCtgacaa-------------------------GGTGCGCCTTTTTGCTTATGTA
>22494<-23147 22374  [bsub-term-20+100.fasta] FW 48-94 15.69
AGATTGTTTTGTCCGTTGCattcggt------------------------GCGGCGGA-TTTTTTATTTTAA
>42497->42856 42836  [bsub-term-20+100.fasta] FW 16-62 16.49
AATAGGGAAAAGGCTTCCGcataaga------------------------CGGGAGCC-TTTTTCAAAGGTA
>43919->44797 44777  [bsub-term-20+100.fasta] FW 15-62 14.80
CAATAAAAAAACGTTCTTGtgtcataa-----------------------CAAGAACG-TTTTTTGTACAAG
>44846<-45136 44726  [bsub-term-20+100.fasta] FW 66-113 14.80
CAATAAAAAAACGTTCTTGtgtcataa-----------------------CAAGAACG-TTTTTTGTACAAG
>45631->47625 47605  [bsub-term-20+100.fasta] FW 22-77 14.14
GATAAACAAAAGGTGTTTCacgtgtaacaattcgt---------------CGAACACC-TTTTGTGTTTCGA
>48627->49940 49920  [bsub-term-20+100.fasta] FW 26-70 15.24
ACTTATGTATTCAGAGGGTtttgc--------------------------GCCCTCTG-TTTTTTTCGTTAT
>51678->52550 52530  [bsub-term-20+100.fasta] FW 20-66 17.61
ACAGTTGAAAACCTGCATAggagagc------------------------TATGCGGG-TTTTTTATTTTAC
>53181->53366 53346  [bsub-term-20+100.fasta] FW 19-66 14.53
AAGGATGTGACCCGGGGGAcgtgctg------------------------TTCCCTGGTTTTTTTATTTTGG
>55864->56157 56137  [bsub-term-20+100.fasta] FW 28-93 16.51
CCAAAAAGCAAGGACTGCTgaaagggctgacataagccttttgcc----GGCGGTCC-TTTTTTAATTCTG
>60128->60358 60338  [bsub-term-20+100.fasta] FW 13-64 14.38
TTCAATAAAAAGCTTTGGTgtagacactag--------------------ACCAAAGCGTTTTTCTGTTCTC
>64097->64633 64613  [bsub-term-20+100.fasta] FW 44-93 16.50
AGGTATGTAAAGAACAGCTctccttggga---------------------CGCTGTTC-TTTTTCATGCGTG
>69624->70010 69990  [bsub-term-20+100.fasta] FW 37-84 17.13
AAATAAATGAAGCATCCGTtcatcccg-----------------------ACGGATGC-TTTTTTATTATCC
>76982->78895 78875  [bsub-term-20+100.fasta] FW 30-75 16.93
TTTCTAAAAAAACTGCCGGctgacg-------------------------CTGGCAGT-TTTTTTATGTAAA
```

```
>79877->80752 80732  [bsub-term-20+100.fasta] FW 2-46 15.90
TGACCAAACTACCCGCTAAgctct-------------------------TTAGCGGG-TTTTTAATTTGAG
>81768->82694 82674  [bsub-term-20+100.fasta] FW 21-64 17.61
AAAAAGCCAAAACTCCCGGttcg--------------------------CCGGGAGT-TTTTTTATATTTC
>88724->90223 90203  [bsub-term-20+100.fasta] FW 17-74 18.31
CTAATAAAAAAGAGCGGTAtcctccatagggaaagga------------TGCCGCTC-TTTTTAAATCCCT
>117529->117708 117688  [bsub-term-20+100.fasta] FW 55-96 14.99
TTACTTGCCAAAACCCGTTc-----------------------------AGCGGGTTTTTTATTGTGGCTT
>118588->119013 118993  [bsub-term-20+100.fasta] FW 23-76 14.75
TGTTTCTTGTCGGGTTGCGagttttaacaagtt----------------CGCAACCC-TTATTCGTGGGAG
>120604->120975 120955  [bsub-term-20+100.fasta] FW 32-76 15.71
CTGTAGGGGAAGCTCGCTTttatg-------------------------AGGCGAGC-TTTTTCTTTGCCA
>132881->134071 134051  [bsub-term-20+100.fasta] FW 25-68 18.12
ATGGTTTTAAACGAGACCCctgt--------------------------GGGTCTCG-TTTTTTGTTTGCT
>149951->150313 150293  [bsub-term-20+100.fasta] FW 33-94 14.41
GTGTATCTAAAGAAGGGCGggacagtttctaactggatcta--------TGCCCTTT-TTTTAGATACTGC
>154299->154691 154671  [bsub-term-20+100.fasta] FW 26-74 14.99
CGTTTTCAAAAAGCTCTCGaccttgggt---------------------TGGGAGCT-TTTTTCTTTCAAT
>157420->158478 158458  [bsub-term-20+100.fasta] FW 18-61 15.35
TAAAAGGTGAACCGGGATTcca---------------------------GATCCCGGCTTTCCCTTTATTC
>177082->178518 178498  [bsub-term-20+100.fasta] FW 19-65 19.11
AAAATAGAGAAGCCCAGATgaatgag-----------------------ATCTGGGC-TTTTTTATATAAC
>178733->179584 179564  [bsub-term-20+100.fasta] FW 14-60 19.07
GGCATAAAAAAACCGCTCAtagggt------------------------TGAGCGGTTTTTTCCGTATAGC
>185192<-186436 185072  [bsub-term-20+100.fasta] FW 31-83 14.02
GCATGAAAAAAACCCTTACttatgtgtaaaa------------------GTAAGGGTCTTTCGTTCAATAA
>200263->202065 202045  [bsub-term-20+100.fasta] FW 20-74 14.97
ATAAATGTTTAACCCCTTTggataagattatct----------------AAAGGGGTGTTTTTATGTCCAA
>211845->213017 212997  [bsub-term-20+100.fasta] FW 25-73 16.46
GAAAGTTCTAACATCCGCTcgttataca---------------------AGCGGGTG-TTTTTTTTAGCGT
>216898->217617 217597  [bsub-term-20+100.fasta] FW 42-102 14.35
TCAGTCAAAAAGGCGTTTTgtaagtcaaggtcaggaaaaa---------AAAATGCC-TTTTTTGATCATG
>226555->227943 227923  [bsub-term-20+100.fasta] FW 19-64 16.56
AACGATAAAAAAGAGACATtcacgg------------------------ATGTCTCT-TTTTTTATTTTTC
>228320->228511 228491  [bsub-term-20+100.fasta] FW 10-53 16.65
ATGAGATTTAGAGCCCTGCcgt---------------------------GCAGGGCTCTTTTATTTAGGAT
>229514->230767 230747  [bsub-term-20+100.fasta] FW 18-66 16.04
TAAATTAAAAAGCTCTCTTcctttatcg---------------------AAGAGAGC-TTTTTGATTACTT
>231337->232956 232936  [bsub-term-20+100.fasta] FW 22-71 16.03
ATATAGAAGAAAACCTTGCgatagttgtc--------------------GCAAGGTT-TTTTGCTTTTAAT
>243881->245035 245015  [bsub-term-20+100.fasta] FW 16-69 14.58
AATAGAGTTTGAACAGGTCttgtcatgggacaa----------------GGCCTGTT-TTTTTCTTTCTCC
>246083->246481 246461  [bsub-term-20+100.fasta] FW 9-61 14.86
TTCAAAGAATAGCAGTTTCcttgattttaag------------------GGAACTGCCTTTTTTATTTATT
>249968->251308 251288  [bsub-term-20+100.fasta] FW 16-61 16.26
AGTGAGCGGTGTCCCCTGTggtaa-------------------------ACAGGGGATTTTTACATATCGC
>252503->253471 253451  [bsub-term-20+100.fasta] FW 14-62 16.54
TGAATAAAAAAAGAACACCtcgtattga---------------------GGTGTTCT-TTTTTTCTATATA
>258521->258796 258776  [bsub-term-20+100.fasta] FW 15-62 15.49
CTCTAAATCAAACAGCCAGaataaaa-----------------------CTGGCTGTTTTCTTTAATTTCA
>259005->260075 260055  [bsub-term-20+100.fasta] FW 22-66 17.23
AATCGAAAAAGAACCTGCCcgga--------------------------GGCAGGTTCTTTTTATTTTGAA
>266711->267655 267635  [bsub-term-20+100.fasta] FW 15-59 16.15
ATATAAACCAAACAGCCGGctgac-------------------------CAGGCTGT-TTTTTTGCTGCGC
>273229->273930 273910  [bsub-term-20+100.fasta] FW 20-70 15.31
ATGGTGAGCAACCGCAGTTgaaacgtaag--------------------AGCTGCGGTTTTTTTAAACCAA
>277334->278272 278252  [bsub-term-20+100.fasta] FW 21-86 15.59
TTTTTATAAAACACACACGtttttcaattacaataagtgtaatta----CGTGTGTG-TTTTTTGATTTTG
>282011->282439 282419  [bsub-term-20+100.fasta] FW 18-62 14.15
TAGAGACAGGACACCGTTCaaatt-------------------------GAACGGTG-TTTTTCTTTGAAA
>285320->285532 285512  [bsub-term-20+100.fasta] FW 18-64 15.32
TGAATGCTGATGAGGCAGAcaccggg-----------------------TCTGCCTC-TTTTTTTATGAAA
>287053->288165 288145  [bsub-term-20+100.fasta] FW 20-67 18.85
AACATGAAAAAGCCCCTGAacactag-----------------------TCAGGGGCTTTTCATATTAATG
>290469->291596 291576  [bsub-term-20+100.fasta] FW 14-59 14.86
GTATTGAAGAAAAGAAGGCgaataa------------------------GCCTTCTT-TTTTTTGGCTTTT
>293053->294129 294109  [bsub-term-20+100.fasta] FW 18-68 18.39
TAATGGAAAAAACCTTGAAaagccaggctt-------------------TTCAAGGT-TTTTTTATTTCTG
>303984->305105 305085  [bsub-term-20+100.fasta] FW 16-63 15.28
AATAGGAAATGGCAGAGAActacagg-----------------------TTCTCTGCTTTTTTTGTGCTGT
>306013->307698 307678  [bsub-term-20+100.fasta] FW 21-65 15.40
TGATTGAAGTAGCCCGGCAttttt-------------------------AGCCGGGT-TTTTTTAGTACAT
>309554->310396 310376  [bsub-term-20+100.fasta] FW 17-66 15.23
ATAACATGAAAAGCAGTTTtccctaggga--------------------AAACTGCT-TTTTTTATAGAAA
>313579->314352 314332  [bsub-term-20+100.fasta] FW 18-68 14.60
TAAAACAAAACAAGGAGGGaggttcaatgt-------------------CCTTCCTT-TTTTGTTACCGAA
>316066->317157 317137  [bsub-term-20+100.fasta] FW 15-66 15.27
```

```
CAATAATAAAAACCCCGCTtgtggaacata-------------------AGCGGGGTATTTCAATTACATC
>317279->318481 318461  [bsub-term-20+100.fasta] FW 14-59 16.81
AGAATAGGAAAAGCACCTCttaaaa------------------------GAGGTGCT-TTCAGCGTGTCGA
>322673->323554 323534  [bsub-term-20+100.fasta] FW 18-60 19.63
TAATCAAAAAAGCAGCCTGtgt---------------------------CAGGCTGC-TTTTTTTGCGTTA
>327171->329153 329133  [bsub-term-20+100.fasta] FW 38-82 15.13
ACTTACCGAAAGAAACCATcaatg-------------------------ATGGTTTC-TTTTTTGTTCATA
>330327->331952 331932  [bsub-term-20+100.fasta] FW 18-68 17.95
TAATAGAAAAAAGCAGTACatgcccagcat-------------------GTACTGCT-TTTTTTATGTTAA
>337860->338678 338658  [bsub-term-20+100.fasta] FW 19-64 15.87
AAGTTGAAGAAAGCCCGCTctcgg-------------------------AGCGGGCTTTTGTCGTGTACAG
>338728<-339321 338608  [bsub-term-20+100.fasta] FW 69-114 15.87
AAGTTGAAGAAAGCCCGCTctcgg-------------------------AGCGGGCTTTTGTCGTGTACAG
>339597->340157 340137  [bsub-term-20+100.fasta] FW 13-56 21.97
CGATGTAAAAAGCCGTGCGcag---------------------------CGCACGGCTTTTTTTATCGTTT
>342110->343066 343046  [bsub-term-20+100.fasta] FW 22-68 16.20
AAATGTGAAAAGCCGCCGCatatcat-----------------------CAGGCGGT-TTTTTTCTGCAAA
>352430->353440 353420  [bsub-term-20+100.fasta] FW 12-58 14.58
CATAAATAAAACAGCCCTTgaggaa------------------------AAGGGCTGCTTGCCATCAGGTG
>365634->366827 366807  [bsub-term-20+100.fasta] FW 17-61 16.59
ATAGAAAAAAAGCCGTCCCatgg--------------------------GGAACGGCTTTTTTTAATGAAA
>366876<-367556 366756  [bsub-term-20+100.fasta] FW 68-112 16.59
ATAGAAAAAAAGCCGTCCCatgg--------------------------GGAACGGCTTTTTTTAATGAAA
>369344->369676 369656  [bsub-term-20+100.fasta] FW 18-63 14.23
TAAGATTTTAAGCATCCAAtgggaa------------------------TTGGGTGC-TTTTGCGTTATTT
>369830->371263 371243  [bsub-term-20+100.fasta] FW 15-62 14.25
TTATAAAGAGTCCCTGAGAgttattc-----------------------TCTCAGGGGTTTTTCATTACAC
>401969->402697 402677  [bsub-term-20+100.fasta] FW 14-65 15.63
ACCGTGATCAAAAGCGGACagcttcggctg-------------------TTCCGCTTTTTTTGTGTTGAAT
>402798->404027 404007  [bsub-term-20+100.fasta] FW 12-60 14.90
TCAATATAAAAGGATCAGCactgtcaat---------------------GCTGATCC-TTTTTAAATTTGA
>414930->415775 415755  [bsub-term-20+100.fasta] FW 14-65 18.29
TCAATAAAAAACAGCCCGCagatcaacatcc------------------GCGGGCTG-TTTCTGATTATAA
>417573->419327 419307  [bsub-term-20+100.fasta] FW 14-59 15.01
TGTCTGAAAAAAGCGCCCCccttta------------------------GGGGTTCT-TTTTTTTCGATTT
>422562->423683 423663  [bsub-term-20+100.fasta] FW 16-64 18.42
AATGATAAAAAGAGCGGGGggatgcggc---------------------CGCCGCTC-TTTTTATGTTCAC
>423788<-424468 423668  [bsub-term-20+100.fasta] FW 11-59 18.42
AATGATAAAAAGAGCGGGGggatgcggc---------------------CGCCGCTC-TTTTTATGTTCAC
>429543->429665 429645  [bsub-term-20+100.fasta] FW 17-64 17.09
GTAAGAACAAGCCCCTTCTcattagcg----------------------AGAAGGGG-TTTTTCTTTTCAA
>434616->435569 435549  [bsub-term-20+100.fasta] FW 18-68 19.18
TAAAACCAAAAAGAGCCTCcgctaaatagc-------------------GGGGCTCT-TTTTTTGTTAATC
>435616<-437034 435496  [bsub-term-20+100.fasta] FW 72-120 15.59
AAAACCAAAAAGAGCCTCCgctaaatag---------------------CGGGGCTC-TTTTTTTGTTAAT
>442530->443918 443898  [bsub-term-20+100.fasta] FW 28-77 16.73
CACGCTCCTGAGAGCTGCCggattttcc---------------------GGCAGCTCTTTTTGTGTTCCGG
>444924->445709 445689  [bsub-term-20+100.fasta] FW 18-70 16.69
TAAACATAAAAAGCGACCCagacatgacatct-----------------GGATCGCT-TTCTTTATTAGGC
>448778->449131 449111  [bsub-term-20+100.fasta] FW 1-44 17.08
GAAAGAGAAAAGGCTCCTGaaac--------------------------CAGGAGCC-TTTTTATTTTTAA
>455623->456372 456352  [bsub-term-20+100.fasta] FW 15-61 17.19
CTATAAAAAAAGAGAGTCCtaagat------------------------GGACTCTCTTTTTAGTTTGGCA
>461984->462625 462605  [bsub-term-20+100.fasta] FW 14-57 19.86
TTCATAGAAAACGCGCCTCatga--------------------------CAGGCGCG-TTTTTTATGTGTG
>465595->466497 466477  [bsub-term-20+100.fasta] FW 13-58 14.57
TTCCATAAAAAGCATCAGTttacca------------------------GCTGATGC-TTTTTCAATATTG
>466683->468767 468747  [bsub-term-20+100.fasta] FW 20-67 15.14
AACCTGCATGGCACACGTCaaaaattt----------------------GGCGTGTG-TTTTTCTGTGGAT
>474284->475093 475073  [bsub-term-20+100.fasta] FW 16-64 16.51
TTTAATAAGAAAGGCTGGAtcataaaga---------------------TCCAGCCT-TTTTGCGCTTCCT
>475137<-475427 475017  [bsub-term-20+100.fasta] FW 72-120 16.51
TTTAATAAGAAAGGCTGGAtcataaaga---------------------TCCAGCCT-TTTTGCGCTTCCT
>476111->478294 478274  [bsub-term-20+100.fasta] FW 18-60 19.08
TAATAAAAAAACCTGTGCCtat---------------------------CGCACAGG-TTTTTTATTGGAC
>483398->485509 485489  [bsub-term-20+100.fasta] FW 11-57 19.00
TACAGAATAAAACAGAGGCgatttta-----------------------GCCTCTGT-TTTTTTATTTTTG
>488383->490107 490087  [bsub-term-20+100.fasta] FW 14-60 16.37
ATTTTAAAAAAACAGGGGCcctaaga-----------------------GCCCTTGT-TTTTTTTTTTTTT
>493110->493928 493908  [bsub-term-20+100.fasta] FW 15-66 14.74
GAATAGCAGAAAGCAGACGgacaccgcgatc------------------CGCCTGCT-TTTTTTAGTGGAA
>494057->494428 494408  [bsub-term-20+100.fasta] FW 46-97 19.09
GTAAATAAAAAAGCGATCCggcacatcagct------------------GGATCGCT-TTTTTTTGGAATC
>499723->500988 500968  [bsub-term-20+100.fasta] FW 16-61 19.28
TCTGAAAAAAGAACGGCCCatccat------------------------GGGCCGTT-TTTTTAATTGTTT
>504246->504581 504561  [bsub-term-20+100.fasta] FW 12-61 14.00
CCTGTGTAATCGCGCTGTTcccttgggg---------------------AACGGCGTGTTTTTCTATACTG
```

>504709->505854 505834 [bsub-term-20+100.fasta] FW 13-57 16.97
AGTCATAAAAAAAAGAGCCttacc-------------------------GGCTCTTT-TTATGTATTGTTT
>507805->508869 508849 [bsub-term-20+100.fasta] FW 19-66 15.72
AATGAAGAAGGACACTGCCggtaaaag----------------------GGCAGTGT-TTTTTCCCGTATA
>510663->512198 512178 [bsub-term-20+100.fasta] FW 24-73 15.97
GATCGATTCAGAGCCCAAAacatgatgt---------------------TTTGGGCTTTTTTATGTTCGTG
>515693->516814 516794 [bsub-term-20+100.fasta] FW 17-62 17.29
ATAACCGCCAAAGGCCAAAcatga-------------------------TTTGGCCTTTTTTTCGTTAGAC
>523207->523806 523786 [bsub-term-20+100.fasta] FW 24-66 15.78
ACCAGAAAAAGAAGCTGGAca----------------------------TCCGGCTTCTTTTTTTTGCGGT
>525300->527633 527613 [bsub-term-20+100.fasta] FW 13-55 15.16
TAAAATAAAAGCACTGCTTac----------------------------GAGCAGTGTTTTTTCCTATAGA
>529236<-530342 529116 [bsub-term-20+100.fasta] FW 31-81 15.08
CGCATCAATGATACCAAGGgttttgacac--------------------CCTTGGTATTTTTTTGTGTTTA
>549953<-550972 549833 [bsub-term-20+100.fasta] FW 7-71 16.16
AAAAAAACTAAAGGGTGATacatattatacatgattaatatgt------ATCACCCTTTTCTTTCTTGTTC
>551765<-552214 551645 [bsub-term-20+100.fasta] FW 45-105 16.90
GCTCTAAAAAAGCCCTAGGttggatatggaatttaccaa----------GCTGGGGCCTTTTTATACATTA
>556476->557162 557142 [bsub-term-20+100.fasta] FW 18-70 15.18
TAATTTTAAAATCACTTTGtctttataaagga-----------------CAAAGTGA-TTTTTTGTTATAC
>558978->559178 559158 [bsub-term-20+100.fasta] FW 29-76 18.65
CGTTTATACAAACAGGCTCtttatata----------------------GGGCCTGT-TTTTTTATGTCTA
>567376->567822 567802 [bsub-term-20+100.fasta] FW 61-104 17.64
TATACAAAAAGAGAAGGCTtgtg--------------------------GGCCTTCT-TTTTTTGTTGTTG
>570085<-570948 569965 [bsub-term-20+100.fasta] FW 37-85 16.82
CGCATACAAAATACCAAGGgccagcaac---------------------CCTTGGTA-TTTTTATACCTTC
>575426<-575812 575306 [bsub-term-20+100.fasta] FW 71-119 15.51
ATCACAGAAATACCAAGGGcgtcaaaa----------------------CCCTTGGTATTTTTTCTGGTTA
>588674->589315 589295 [bsub-term-20+100.fasta] FW 34-80 14.49
AAATTGCCCAAACGTACATgcccga------------------------ATGTACGTTTTTTTCATTTCAT
>589431->591605 591585 [bsub-term-20+100.fasta] FW 17-63 16.74
GTAATAGAAAAAGCAGATCttttat------------------------GATCTGCTCTTTTGCTTATGGT
>592017<-592919 591897 [bsub-term-20+100.fasta] FW 17-63 14.39
TCCAAGAAAAATAGAGTCCataagag-----------------------GGACTCTA-TTTTTTGAAGTGC
>598443->598781 598761 [bsub-term-20+100.fasta] FW 27-68 15.80
AAGGCAAAAAAAGAACTCAaa----------------------------TGAGTTCT-TTTTTTATTCTTC
>604450<-606093 604330 [bsub-term-20+100.fasta] FW 64-105 15.12
ATAAAGAAAAAGGACAACAgc----------------------------TGTTGTCC-TTTTCAATGCCGC
>609103->611760 611740 [bsub-term-20+100.fasta] FW 19-65 14.18
AATCGAGAGAAGAGTGATGaatggt------------------------CATCACTCTTTTTTCATGACTA
>613356->614564 614544 [bsub-term-20+100.fasta] FW 13-57 16.20
ATTCATAAAAAAACCTGACatgac-------------------------GTCAGGTT-TTTTGTTTTATTT
>615587->616261 616241 [bsub-term-20+100.fasta] FW 24-74 14.97
GTATGATCCGGTACCAAGGgagagcagaac-------------------CCTTGGTA-TTTTTTTATGTAT
>617853->618998 618978 [bsub-term-20+100.fasta] FW 20-63 14.55
AGAAAAAGAACTCCCGTACctt---------------------------GTACGGGAGTTCTTGATTTAAA
>624209->624826 624806 [bsub-term-20+100.fasta] FW 17-60 17.05
ATAATAAAAAATCCTCCTTttac--------------------------AAGGAGGA-TTTTTTTGCTGTT
>647656->648420 648400 [bsub-term-20+100.fasta] FW 17-64 14.45
ATAATAAAAAATCCATTTCtcatgcga----------------------GAAATGGA-TTTTCTTTATTTT
>656245->657414 657394 [bsub-term-20+100.fasta] FW 45-91 17.05
TAAAAAGTATCGGAGCTGGataaaa------------------------CCAGCTCCGTTTTTTATCTTTA
>664036->664386 664366 [bsub-term-20+100.fasta] FW 40-87 17.43
AAGAAACGAAAGGCCAACTgcgacttc----------------------AGTTGGCC-TTTCCTATTTATA
>664492<-666981 664372 [bsub-term-20+100.fasta] FW 34-81 17.43
AAGAAACGAAAGGCCAACTgcgacttc----------------------AGTTGGCC-TTTCCTATTTATA
>676159->677580 677560 [bsub-term-20+100.fasta] FW 22-63 16.69
TTAAAAAAGAATCCGCACCcg----------------------------AGTGCGGA-TTCTTTTTGGTTT
>677628<-678668 677508 [bsub-term-20+100.fasta] FW 74-115 16.69
TTAAAAAAGAATCCGCACCcg----------------------------AGTGCGGA-TTCTTTTTGGTTT
>679107->679478 679458 [bsub-term-20+100.fasta] FW 30-75 15.43
ATAATCTGGAAAGCAAGAAgtcaca------------------------TTCTTGCT-TTTTCTATTGGTG
>679544->680590 680570 [bsub-term-20+100.fasta] FW 16-62 17.32
ACTAATAAAAACCACTCGGcatgag------------------------CTGAGTGGCTTTTTTAATGGAT
>686679->687551 687531 [bsub-term-20+100.fasta] FW 13-57 14.33
AAAAATAATGGAGCTTCCTgtcac-------------------------AGGAAGCT-TTTTTGTGTGTTT
>694180->695502 695482 [bsub-term-20+100.fasta] FW 22-65 18.60
GAATGAAAAAACCAGCTGCactg--------------------------GCAGCTGG-TTTTTTTTGTTGC
>695704->696507 696487 [bsub-term-20+100.fasta] FW 17-60 21.00
TTAATGAAAAAACCGGCTAatcc--------------------------TAGCCGGT-TTTTTTATGTCAC
>704946->706376 706356 [bsub-term-20+100.fasta] FW 30-73 15.53
AATGACATAAAGGCAGCGCagtt--------------------------CGGCTGCC-TTTCTCTTTCTGC
>709653->710921 710901 [bsub-term-20+100.fasta] FW 19-63 17.72
AAGTGAGGAAAACCCGCAGaatag-------------------------CTGCGGGT-TTTTTGTTATCAA
>715937->716251 716231 [bsub-term-20+100.fasta] FW 13-63 20.77
CCAAATAAAAAACCGCCCTgccgtctggca-------------------AGGGCGGT-TTCTTTATGAAAC
>723141->724331 724311 [bsub-term-20+100.fasta] FW 16-66 15.84

```
ACTGATAGAAAACCCTTGTgccatattggc-------------------ACAAGGGT-TTTTATGTTACAA
>724493->725503 725483  [bsub-term-20+100.fasta] FW 19-68 16.72
AGATAGAAAAAGCCAATGGgattccagtc-------------------CCATTGGC-TTTTATAATGTAT
>730192->731445 731425  [bsub-term-20+100.fasta] FW 16-59 19.01
GCTAATAAAAAAGCAGCCCttag-------------------------AGGCTGCT-TTTTTTATGGTCA
>732422->735619 735599  [bsub-term-20+100.fasta] FW 20-63 17.68
AAAAACAAAAAGCCTCAGCtct--------------------------GCTGAGGCTTTCAGCTTGTTGA
>751916->752638 752618  [bsub-term-20+100.fasta] FW 16-61 16.63
AGTAAGGAGTGAGCAGGCTgttat------------------------GGCCTGCTTTTTTTTGTCCCGG
>754428->755288 755268  [bsub-term-20+100.fasta] FW 18-64 14.29
TAGTCTAGAAAGACCTTCGgcgcttc----------------------CGAAGGTC-TTCTTATTGAAAA
>783889->784584 784564  [bsub-term-20+100.fasta] FW 21-69 15.38
CTCTTAGAAAAAAGCTTGCagcaaggc---------------------GCAAGCTTTTTATTGATTGTAT
>787500->788144 788124  [bsub-term-20+100.fasta] FW 16-63 15.08
ATTAATGAAAAAGCGTCTGtcattgga---------------------TAGGCGCT-TTTTGCCATACAT
>788144->789136 789116  [bsub-term-20+100.fasta] FW 10-52 16.32
AGACGCTATAGCCTGTCCGgat--------------------------CGGACGGG-TTTTTTAGTCTTT
>789826->790935 790915  [bsub-term-20+100.fasta] FW 18-66 14.72
TAAATAGGAAAAGTCCAGAaagtgatt---------------------TCTGGACTTTTTAGTCTTGCTC
>795780->797741 797721  [bsub-term-20+100.fasta] FW 20-79 16.70
AGATGAAAAAGAGCCTTGAgcgggcgcattgccttcgc----------TCAAGGCTCTTTTTTTGGTTAT
>801859->802794 802774  [bsub-term-20+100.fasta] FW 15-58 19.56
GATTAAAAAACCCCCTGCCgcct-------------------------GGCAGGGG-TTTTTTCAGCTAT
>808070->808930 808910  [bsub-term-20+100.fasta] FW 13-59 18.09
AAGAGTAAGAGACCGGGGAcaaaca-----------------------TCCCCGGTCTTTTTCTTATCCT
>809065->810954 810934  [bsub-term-20+100.fasta] FW 13-58 19.80
AAAGCTAAAAAGCGTGGCCgcagca-----------------------GGCCGCGC-TTTTTTTCACATA
>812136->813326 813306  [bsub-term-20+100.fasta] FW 18-63 17.62
TAATAACGAAAGACACTGCgcatat-----------------------GCAGTGTC-TTTTTATTCGTTA
>813892<-815448 813772  [bsub-term-20+100.fasta] FW 77-120 16.92
GCAGTAAAAAAACCCGCCCTttag------------------------CAGGCGGG-TTTTTTTATAAAT
>816819->817265 817245  [bsub-term-20+100.fasta] FW 27-72 14.12
CGAAACACAAAAGGAGGGCaaaaa------------------------GCCCTCCCGTTTTCATCTTCAT
>817318<-818337 817198  [bsub-term-20+100.fasta] FW 74-119 14.12
CGAAACACAAAAGGAGGGCaaaaa------------------------GCCCTCCCGTTTTCATCTTCAT
>826963->827310 827290  [bsub-term-20+100.fasta] FW 23-72 14.02
CAAGGAAAAAACCAAAAGGccaatgtcgg-------------------CCTTTTGG-TTTTTTTGCGGTC
>827501->828763 828743  [bsub-term-20+100.fasta] FW 22-68 15.11
AAAGTGAAAAACACAAAGGgtgctaa----------------------CCTTTGTG-TTTTTTAATTAAT
>828890->830326 830306  [bsub-term-20+100.fasta] FW 18-60 18.58
TAGAAAGAAAAAGGCAGACgcg--------------------------GTCTGCCT-TTTTTTATTTTCA
>832916->833695 833675  [bsub-term-20+100.fasta] FW 12-51 14.18
GGAAATTAAAATCCCGACA-----------------------------TCCCGGGA-TTTTTTTCATGCC
>835248->836042 836022  [bsub-term-20+100.fasta] FW 10-52 18.26
CGTCTATTTGACACCCGCAcca--------------------------CGCGGGTG-TTTTTTATTGTTT
>837585->838250 838230  [bsub-term-20+100.fasta] FW 16-64 18.57
AATAGAAAAAAGCCGCGCAtatcaacg---------------------TGCGCGGCTTTGCCATATTTAA
>840164->841522 841502  [bsub-term-20+100.fasta] FW 14-59 19.26
CAAATAAAAAAGCGGAGAGggcaac-----------------------CTCTCCGC-TTTTTCTTATTTA
>843892->844152 844132  [bsub-term-20+100.fasta] FW 24-74 14.52
GTCTTGTCTGCAGGATGGCgattgataaaa------------------GCCATCCT-TTTTATGTTCCAA
>853919->854584 854564  [bsub-term-20+100.fasta] FW 18-65 18.70
TAAAAGAAGAAAGCTGCTCgcatagc----------------------GAGCAGCTCTTTTTTATGCCTG
>859252->859770 859750  [bsub-term-20+100.fasta] FW 16-62 19.31
AATAAGAAAAAAACGGACGCTcttgg----------------------GCGTCCGTTTTTTTGTTGCTTA
>859810<-861000 859690  [bsub-term-20+100.fasta] FW 77-120 17.99
ATAAGAAAAAAACGGACGCTcttg------------------------GGCGTCCG-TTTTTTTGTTGCT
>865838->866632 866612  [bsub-term-20+100.fasta] FW 17-63 16.11
ATAAATAAAAAACTGATTCcaactcg----------------------GAATCAGT-TTTTTTGTTTATT
>868779->868964 868944  [bsub-term-20+100.fasta] FW 11-53 16.96
TTGGGAATAAAACCGCGTGccc--------------------------GCCGCGGT-TTTTTTATTGGCA
>872908->874308 874288  [bsub-term-20+100.fasta] FW 49-114 17.08
TTTCCTAATAAGACCTGGAtttcggtaaaataaacaattccgatt----TCCGGGTC-TTTTTCGTGCGCA
>877587->878276 878256  [bsub-term-20+100.fasta] FW 49-93 16.18
ATCATTGTCAAAGGCCGGGtgata------------------------TCCGGTCT-TTTTTTTGCATGC
>881772->883148 883128  [bsub-term-20+100.fasta] FW 21-71 22.35
TAAAGGAAAAAGCAGGCGCatggatataag------------------GCGCCTGC-TTTTTTATTGTTG
>885135->885314 885294  [bsub-term-20+100.fasta] FW 17-63 18.14
TTAAACAAAAATGGCCCGCttcataa----------------------GCAGGCCA-TTTTGTTATCCGC
>891721->893304 893284  [bsub-term-20+100.fasta] FW 16-57 15.05
AATAAAGACTGCCCTCCTTtt---------------------------CGGGAGGG-TTTTCGTTTGCCG
>897516->898373 898353  [bsub-term-20+100.fasta] FW 16-67 17.68
TTTAACTGCAATCCCCTTGccgaaataacgg-----------------CAGGGGGA-TTTTTTATTTTTG
>904516->905178 905158  [bsub-term-20+100.fasta] FW 43-84 15.31
CTTCTCTGAAAAGGCCTTTt----------------------------ACAGGCCTTTTTTTTCATGCCCT
>907474->908631 908611  [bsub-term-20+100.fasta] FW 14-58 17.60
AGCCTAAAAAACATCTGCCgttta------------------------GGCAGATG-TTTTTTTGCATGT
```

```
>908704<-909252 908584  [bsub-term-20+100.fasta] FW 41-85 17.60
AGCCTAAAAAACATCTGCCgttta-------------------------GGCAGATG-TTTTTTTGCATGT
>910346->911434 911414  [bsub-term-20+100.fasta] FW 17-63 17.48
GTGAAAAACAAGCGGCAGGagggct------------------------CCTGCCGCGTTTCTTTACTTCT
>920979->922010 921990  [bsub-term-20+100.fasta] FW 17-68 14.08
TTAATCGTTACACCCATTTtcttaaaaaga-------------------AAATGGGT-TTTTTTGATAATG
>923094->923678 923658  [bsub-term-20+100.fasta] FW 14-64 18.73
AATGTAAAAAACCCCTTGTctcaaacggag-------------------ACAAGGGG-TTTTTCATTAACG
>925936->926250 926230  [bsub-term-20+100.fasta] FW 20-62 16.22
AAGCGTAACAGGAGGCTGAtga---------------------------TCAGCCTC-TTTTTGTTTGCAG
>926393->927586 927566  [bsub-term-20+100.fasta] FW 14-58 17.03
AGGGTAAAAAACAGCTGCAgtgta-------------------------TGCAGCTG-TTCTTCTTTACCG
>927896->928165 928145  [bsub-term-20+100.fasta] FW 12-59 18.07
TTGCATTAAAAACCTGCCGttaacgac----------------------CGGCAGGT-TTTTTCATTTTCT
>937406->937660 937640  [bsub-term-20+100.fasta] FW 27-77 14.87
AAACAGAAAAAAGCACTTCatcttcgggtg-------------------GAAGTGCT-TTTTTCTGTTTGA
>938827->940596 940576  [bsub-term-20+100.fasta] FW 68-109 15.09
ATATAAAAAAACCCGGCTGtt----------------------------TAACCGGG-TTACTCTTCTGTT
>941954<-943132 941834  [bsub-term-20+100.fasta] FW 74-116 15.18
CCATAAAAAAACAGGAGCTcaa---------------------------AGCTCCTG-TTCAAGGTATTTG
>943993->944430 944410  [bsub-term-20+100.fasta] FW 16-62 17.03
ATTAAAAATAAGCTGACCGcacgaaa------------------------CGGTCAGC-TTATTTTATTTCG
>952879<-953655 952759  [bsub-term-20+100.fasta] FW 41-86 17.23
ATACTGGAAAACCCGTTTCttaaca------------------------GAAACGGG-TTTTTATTTTTTA
>953797->954000 953980  [bsub-term-20+100.fasta] FW 13-70 16.32
AGCAATAAAAAAGAGACGGcatttcatttttgagatc------------CCGTCTCT-TTTTGTGGTTTTT
>958798->959016 958996  [bsub-term-20+100.fasta] FW 1-44 17.45
AATATAAAAAAACCCGCTGacta--------------------------CAACGGGT-TTTTGCATTTCTC
>959043<-960494 958923  [bsub-term-20+100.fasta] FW 74-117 17.45
AATATAAAAAAACCCGCTGacta--------------------------CAACGGGT-TTTTGCATTTCTC
>963530->964666 964646  [bsub-term-20+100.fasta] FW 25-88 17.17
AACTAAAATACAGCCCCCCgaacatggtatgatattcaaaaaa------AGGGGGCT-TTTTTTCTGAAAA
>965418->965687 965667  [bsub-term-20+100.fasta] FW 16-68 18.95
GGTAAGAAAAAACTCCGGCcgattacagcgc-------------------GCCGGAGTTTTTTTCGTTTTTC
>969644->970126 970106  [bsub-term-20+100.fasta] FW 16-64 20.49
AATAGTGAAAAACCCGCTCatcgatgat---------------------GGGCGGGT-TTTTTTGCGGATG
>971608->972315 972295  [bsub-term-20+100.fasta] FW 33-78 20.64
TTATACAAAAAGGGTGCTCtttcag------------------------GAGCACCC-TTTTTTATTCTTT
>972665->974560 974540  [bsub-term-20+100.fasta] FW 20-61 14.67
AGCAACGTGACCCGTCCGAa-----------------------------TAGGGCGGGTTATTTTGTCCCA
>974740->975918 975898  [bsub-term-20+100.fasta] FW 14-55 15.64
TTCGTAATGAAAAGCCCATtt----------------------------CAGGGCTT-TTTTTTATTTAAT
>979448->979822 979802  [bsub-term-20+100.fasta] FW 17-70 15.67
GTAATGAAAAAGGCAAATCcgtttactcatgcg-----------------GGTTTGCC-TTTTTTGCTGTTT
>982738->983679 983659  [bsub-term-20+100.fasta] FW 62-104 15.24
AAAATAAAAACTGCAGGTTtg----------------------------CACCTGCAGTTTTTCATATCAA
>983771<-983974 983651  [bsub-term-20+100.fasta] FW 70-112 15.24
AAAATAAAAACTGCAGGTTtg----------------------------CACCTGCAGTTTTTCATATCAA
>986494->987885 987865  [bsub-term-20+100.fasta] FW 20-68 14.51
ACATATGAAAACGTGTAATccaagagg----------------------ATTACACGTTTTTTTAACGGCC
>988530->989099 989079  [bsub-term-20+100.fasta] FW 19-61 20.07
AAATGAAAGAAGCCGCACAatt---------------------------TGTGCGGC-TTTTTCTTTGCGT
>996681->997103 997083  [bsub-term-20+100.fasta] FW 18-61 14.49
TAATAAGAAAGAGCTTGCAgtg---------------------------TGCAGGCTCTTTCTTCACATAA
>1004480->1006147 1006127  [bsub-term-20+100.fasta] FW 17-58 18.50
ATAAATCATAACGGGCTGTct----------------------------GCAGCCCG-TTATTTCTTTTTA
>1015099->1016505 1016485  [bsub-term-20+100.fasta] FW 15-60 16.31
TTATAAAAAAGAGGATGCAgcgttc------------------------TGCATCCT-TTTTATTTTCCAG
>1018452->1019483 1019463  [bsub-term-20+100.fasta] FW 22-66 16.25
TTTTAGAGAAAACCCGTTCattg--------------------------GAACGGGTTTTTTTCATTAGAC
>1021711->1022580 1022560  [bsub-term-20+100.fasta] FW 20-61 18.27
ATCAACGAAAAACCAGCTCaa----------------------------GAGCTGGT-TTTTTGTGTGGTG
>1024345->1025700 1025680  [bsub-term-20+100.fasta] FW 14-59 18.66
ATTTTAAAAAACAGGCTCCgtttcc------------------------GGGGCCTG-TTTTTTATTTAAT
>1027254->1027682 1027662  [bsub-term-20+100.fasta] FW 17-60 15.83
ATAACGAAAAAAGACGCCTgaaa--------------------------AGGCGTCT-TTTTTAAAAGCCA
>1029745->1030740 1030720  [bsub-term-20+100.fasta] FW 15-58 15.79
ATATAACAAAAGCTGAGGGcgaa--------------------------TCCTCAGC-TTTTTGGCGTTTA
>1038133->1038240 1038220  [bsub-term-20+100.fasta] FW 52-94 17.90
TTGAGAAATGACCCCGGCGttt---------------------------CGCCGGGG-TTTTGTGCTGCAT
>1038389->1039504 1039484  [bsub-term-20+100.fasta] FW 17-64 14.42
TTAAAAGAGAGACTTCGTCtgacaga-----------------------GGCGGAGTCTTTTTCATGGTAA
>1041474->1042322 1042302  [bsub-term-20+100.fasta] FW 22-74 16.38
TGAACGAAGAAAAGAGCCCgcttttacagcg------------------GGGCTCTT-TTTATTTTTTAAC
>1046552->1048573 1048553  [bsub-term-20+100.fasta] FW 20-65 16.11
ACGCTCAAAAACCCAAAACaatcgt------------------------GTTTTGGG-TTTTTGGTTTATG
>1048622<-1049242 1048502  [bsub-term-20+100.fasta] FW 71-116 16.11
```

```
ACGCTCAAAAACCCAAAACaatcgt------------------------GTTTTGGG-TTTTTGGTTTATG
>1054226->1054579 1054559  [bsub-term-20+100.fasta] FW 19-64 18.00
AATAAGCAAAATCCCTTCTtggata------------------------AGAAGGGA-TTTTTTATTCACT
>1054623<-1055696 1054503  [bsub-term-20+100.fasta] FW 75-120 18.00
AATAAGCAAAATCCCTTCTtggata------------------------AGAAGGGA-TTTTTTATTCACT
>1057792->1058664 1058644  [bsub-term-20+100.fasta] FW 1-45 16.18
AAACGACAAAGCAGCACTGatta--------------------------CAGTGCTGCTTTTTTTATCCCT
>1062956->1064215 1064195  [bsub-term-20+100.fasta] FW 17-64 16.74
CTGATTGGAAAACAGCCTGgggattct----------------------CAGGCTGT-TTCTTTATTGACA
>1068518->1069462 1069442  [bsub-term-20+100.fasta] FW 18-71 15.65
TAACTTAAAAAGGACTGATgctgacatattcag----------------CTCAGTCC-TTTTTGATGCGTC
>1069587->1069799 1069779  [bsub-term-20+100.fasta] FW 14-66 19.11
CGCATAAAAAAAGCTGTGCggctcaatgagcc-----------------GCACAGCT-TTTTTTATTATTT
>1086723->1087655 1087635  [bsub-term-20+100.fasta] FW 57-110 17.38
TAGTCATCATCGGCGGCGGcattaccggtttag----------------CCGCCGCC-TTCTATATGGAAA
>1087670->1089082 1089062  [bsub-term-20+100.fasta] FW 12-55 18.08
TTCAGCTAAAACCTCCGCTttat--------------------------CGCGGAGG-TTTTTTTGATGTG
>1089874->1092201 1092181  [bsub-term-20+100.fasta] FW 16-67 14.42
CATAAAGAAAAAGAACTTGtttccttggaaa------------------CAAGTTCT-TTTTTTGTTACAT
>1095588->1095995 1095975  [bsub-term-20+100.fasta] FW 13-65 17.85
AAGATTAAAAAAGCCTGCGgggcaagaaccc------------------CGCAGGCTTTTCATTAACCAGA
>1100452->1101993 1101973  [bsub-term-20+100.fasta] FW 18-62 17.26
TAAAATGAAAAAGCGAAGCggtta-------------------------GCTTCGCT-TTTTCATTTTCAA
>1102576->1103856 1103836  [bsub-term-20+100.fasta] FW 13-62 17.59
ACAAATAAAAAGAAGCAGGtatggaggaa--------------------CCTGCTTC-TTTTTACTATTAT
>1103895<-1105040 1103775  [bsub-term-20+100.fasta] FW 77-120 14.39
AATAAAAAGAAGCAGGTATggag--------------------------GAACCTGC-TTCTTTTTACTAT
>1107130->1108176 1108156  [bsub-term-20+100.fasta] FW 17-58 16.83
ATAAAAGAAAAGACAGGCAaa----------------------------CGCCTGTC-TTTTTCTTATTTG
>1115170->1116027 1116007  [bsub-term-20+100.fasta] FW 16-60 16.90
GATAATAAAAAGCCAATCCttgaa-------------------------GGATTGGC-TTATTCGCTCTGC
>1118322->1118591 1118571  [bsub-term-20+100.fasta] FW 19-65 17.10
AAATAAGAAAAAGCAATCTggtgtcc-----------------------AGATTGCT-TTTTTTATGCTTA
>1121648->1122154 1122134  [bsub-term-20+100.fasta] FW 12-58 15.69
GCCGAATAAAAAGACGCTAattgaag-----------------------AGGCGTCT-TTTTGTATGCCGT
>1123910->1124437 1124417  [bsub-term-20+100.fasta] FW 13-56 17.95
TTAAATAAAAAACACGCACtgcg--------------------------GTGCGTGT-TTTTCGCTTTGTT
>1125872<-1126924 1125752  [bsub-term-20+100.fasta] FW 60-106 16.70
CTATATGAAAAAGCAGGAAagtaaa------------------------TTCCTGCTTTTTTAGTAACTTC
>1132971->1134698 1134678  [bsub-term-20+100.fasta] FW 13-58 19.16
CCAAATAAAAAGAGGGTTCtttttt------------------------GAACCCTC-TTTTTTACGCTGC
>1147627->1147929 1147909  [bsub-term-20+100.fasta] FW 15-69 17.30
AAATGACTAAAAAGCAGCCctctctttgcagagc---------------GGCTGCTT-TTATCCGTTTCCA
>1151716->1152621 1152601  [bsub-term-20+100.fasta] FW 21-65 14.85
ATAAAACTGGAGGGCGGACccgg--------------------------ACCCGCCCGTTTTTTCTGATAA
>1152737->1153093 1153073  [bsub-term-20+100.fasta] FW 27-71 14.35
AACCTATGAACCCGGCTCTttgat-------------------------AGAGCTGG-TTTTTTTTATATT
>1153261->1155945 1155925  [bsub-term-20+100.fasta] FW 16-60 20.84
AGTAACCAAAAAGCGGTGCtcgat-------------------------GCACCGCT-TTTTTATTTGCGC
>1159395->1160219 1160199  [bsub-term-20+100.fasta] FW 14-62 19.64
TATCTAAAAAACACCCGGCcttgattga---------------------GCCGGGTG-TTACGCCATACCT
>1162621->1163634 1163614  [bsub-term-20+100.fasta] FW 10-51 15.42
TCAACACATGAGCCCGCTAat----------------------------GAGCGGGT-TTTTTCATTATGA
>1163843->1164871 1164851  [bsub-term-20+100.fasta] FW 49-94 16.84
CCGCAAAAAAAGCAAGGGCatttta------------------------GCCCTTGC-TTTCTCTTTTCTC
>1168517->1169323 1169303  [bsub-term-20+100.fasta] FW 15-58 15.80
TCATAAGAAAAAACCCGACattg--------------------------ATCGGGTT-TTTTATATAATTC
>1180973->1181869 1181849  [bsub-term-20+100.fasta] FW 18-75 21.07
TAGCATGAAAACGGCTCGCctgcagcagactgcgga-------------GCGAGCCGTTTTTTTATGTCTA
>1185081->1185374 1185354  [bsub-term-20+100.fasta] FW 18-65 17.83
TAGAAAAAGAAGGGCAGAGcgaaatat----------------------CTCTGCCC-TTCTTTTTTGGGA
>1185510->1187126 1187106  [bsub-term-20+100.fasta] FW 20-69 15.96
AGCAAACAAAAACAGTCAGgacacagagt--------------------CTGACTGT-TTTTGTTTGCTCA
>1189119->1189478 1189458  [bsub-term-20+100.fasta] FW 11-59 16.35
AGTCAAATAAGAGGCTATGgcgagtcgc---------------------CATAGCCT-TTTTTTATTGCAG
>1191727->1192035 1192015  [bsub-term-20+100.fasta] FW 19-68 19.95
AAAACAAAAAAGGACAGCCtgacatgtga--------------------GGCTGTCC-TTTTTTACAGCTG
>1193805->1194299 1194279  [bsub-term-20+100.fasta] FW 2-47 18.44
GATGATGAAAACAGGCTGAcgcgg-------------------------TCAGCCTGTTTTTTTATGCGGC
>1202927->1203886 1203866  [bsub-term-20+100.fasta] FW 2-48 15.97
ATCATCAAAAAACTGCTGAgccaaac-----------------------TCAGCAGT-TTTTTTGATGGCA
>1203972->1204151 1204131  [bsub-term-20+100.fasta] FW 20-68 14.83
ACCACTAAAAAACCCAAAAcaataaatg---------------------TTTTGGGT-TTTTGGCTTTTTA
>1204631->1205365 1205345  [bsub-term-20+100.fasta] FW 25-72 18.25
TATAATGACAACAGCCTTCtcttttgc----------------------GGAGGCTG-TTTTGTTTTGGGA
>1208650->1209891 1209871  [bsub-term-20+100.fasta] FW 20-68 17.49
ATCAAGCCAAACCGGCTGCccttaaagg---------------------GTAGCCGG-TTTTTTTTGTGTG
```

```
>1215681->1216592 1216572  [bsub-term-20+100.fasta] FW 55-120 14.37
AATCAGAAAAAAGCAGGTCtcctttagtcagaaaatatacaaaga----GATCTGCT-TTTTATGATTCTT
>1216798->1217550 1217530  [bsub-term-20+100.fasta] FW 16-59 18.05
ACTGAAAAAAACCGCTCTTtgca--------------------------AAGAGCGG-TTTTTTGATTAGC
>1227169->1227564 1227544  [bsub-term-20+100.fasta] FW 28-74 16.44
TATCATCAAAAGAAGGCTGagtcat------------------------CAGCCTTCTTTTATTTTTCAAC
>1228540->1229196 1229176  [bsub-term-20+100.fasta] FW 13-61 15.08
CATCATAGCAAACCGATTTccttccgg----------------------AAATCGGTTTTTTTTATGCACG
>1230738->1232567 1232547  [bsub-term-20+100.fasta] FW 19-66 15.21
AAAAAAGTAAGCCTGTGCGgaaatga-----------------------CGCACAGGCTTTTTTAAAACCC
>1233085<-1233912 1232965  [bsub-term-20+100.fasta] FW 12-57 18.42
AGACATATGAAACCGCGCTtatccc------------------------GGCGCGGT-TTCTTTAGTGCTA
>1247226->1248035 1248015  [bsub-term-20+100.fasta] FW 26-70 18.01
ATCTATATCAAGCAATCCCgaata-------------------------GGGATTGC-TTTTTTATTTCTT
>1248136->1248819 1248799  [bsub-term-20+100.fasta] FW 23-75 15.45
AAAAAGAAAAAAGCTGCCCggctgatgttgcc-----------------GGACAGCT-TTTTTTACATAGA
>1248913<-1249359 1248793  [bsub-term-20+100.fasta] FW 29-81 15.45
AAAAAGAAAAAAGCTGCCCggctgatgttgcc-----------------GGACAGCT-TTTTTTACATAGA
>1255907<-1256329 1255787  [bsub-term-20+100.fasta] FW 39-87 17.87
TATGTATAAAAAACCGTTCataccaga----------------------GAACGGTTTTTTTATATGCAGT
>1259077->1260249 1260229  [bsub-term-20+100.fasta] FW 14-61 18.72
TCGTTAAAAAAGAAGCCGGggatagt-----------------------CCGGCTTCTTTTATTATTGTTC
>1263173<-1264402 1263053  [bsub-term-20+100.fasta] FW 21-71 17.15
AATTACAAAAATCCCCTGAaccactctaga-------------------TCAGGGGA-TTGCTTTCCCAAC
>1266600->1266884 1266864  [bsub-term-20+100.fasta] FW 14-55 17.21
TAAATAATAAGAGCATCCTgc----------------------------GGGGTGCT-TTTTTTGTTCCCT
>1267045->1267572 1267552  [bsub-term-20+100.fasta] FW 61-107 14.03
TATAACTTCTAAACCTTCCtataaaa-----------------------TGAAGGTT-TTTTATTTGATTT
>1269204<-1269551 1269084  [bsub-term-20+100.fasta] FW 33-83 17.29
AGCGAAAATAAGGTCCCTTctctttttaga-------------------GGGGGACC-TTATTTTATTCAT
>1275281->1275763 1275743  [bsub-term-20+100.fasta] FW 21-61 17.27
TCCAAACAAAAGCAGGCGCa-----------------------------GTGCCTGC-TTTTGTAATTAAG
>1275809<-1276315 1275689  [bsub-term-20+100.fasta] FW 75-115 17.27
TCCAAACAAAAGCAGGCGCa-----------------------------GTGCCTGC-TTTTGTAATTAAG
>1276547<-1276951 1276427  [bsub-term-20+100.fasta] FW 16-77 17.57
CAGACCAAAAACGGGTAGGaaaatgctgatgttaatgtgtc--------CCTACCCG-TTTTCTGTTCCTG
>1280598->1282001 1281981  [bsub-term-20+100.fasta] FW 19-62 18.85
AAATTAGAAAAGGCTGTCCgtac--------------------------GGACAGCC-TTTTTTCTATTTA
>1288767->1289408 1289388  [bsub-term-20+100.fasta] FW 25-78 14.94
TAATTTACAGTTGCACGTTtttcatctgtggaa----------------AACGTGCA-TTTTTTATAGCGA
>1289601->1290113 1290093  [bsub-term-20+100.fasta] FW 17-56 15.63
CTAAAAAAGAAAGAGCCTG------------------------------TGGGCTCT-TTCTTTTTTATAA
>1292026->1293204 1293184  [bsub-term-20+100.fasta] FW 21-65 14.81
AAACATAAAAACCGAAATCaattt-------------------------GATTTCGG-TTTTCTGTTTAAA
>1293607->1294419 1294399  [bsub-term-20+100.fasta] FW 22-69 18.12
AAGTACAAAAAGCCGCTTTtccctcgg----------------------AAAGCGGC-TTTTGTCTGTCAT
>1310788->1312281 1312261  [bsub-term-20+100.fasta] FW 18-64 17.03
TAATAAGAAAAGGCTATCCttcaagc-----------------------GGATAGCC-TTCTTCATTAAAT
>1313922->1315193 1315173  [bsub-term-20+100.fasta] FW 16-64 19.02
ACTAAAAGAAAGCACGGGTgtttgaaaa---------------------ACCCGTGC-TTTTTTGTTGCGG
>1316464->1316598 1316578  [bsub-term-20+100.fasta] FW 21-60 17.16
TGCATAAAAAAAGACCCTT------------------------------AGGGGTCT-TTTTTATTTCTTC
>1323616->1323822 1323802  [bsub-term-20+100.fasta] FW 45-114 15.62
CGGAAAGCCTGCGGACACTgatcaactgcacagcatttgtgcgttgattGGTGTCCG-TTTTTTATTTGCC
>1323938->1324447 1324427  [bsub-term-20+100.fasta] FW 59-102 16.37
CATGACACTAAGCGGCTGGctga--------------------------TCAGCCGC-TTTTATGAATAAA
>1346760->1347653 1347633  [bsub-term-20+100.fasta] FW 26-69 19.66
GACCATAAAAATCCCGGAGccg---------------------------CTCCGGGATTTATTTTTTCTTC
>1355913->1356884 1356864  [bsub-term-20+100.fasta] FW 17-65 19.01
ATAACCTCAAACCCCCTGTccgtaatgg---------------------ACAGGGGG-TTTTTATGCTCTG
>1371606->1372016 1371996  [bsub-term-20+100.fasta] FW 17-65 16.73
GTGAACGTTAAACCCAAAAcataagttg---------------------TTTTGGGT-TTTTTTGTACTTT
>1372255->1373454 1373434  [bsub-term-20+100.fasta] FW 23-70 14.08
ACATACATGAAACTCTTCTcctttcag----------------------AGAAGAGT-TTTTTATTGAGAT
>1373531<-1373755 1373411  [bsub-term-20+100.fasta] FW 46-93 14.08
ACATACATGAAACTCTTCTcctttcag----------------------AGAAGAGT-TTTTTATTGAGAT
>1376707->1377609 1377589  [bsub-term-20+100.fasta] FW 19-62 15.41
AGACTGCAAGAGGCCCGCGcaa---------------------------TGCGGGCTATTTTTGATGACAA
>1379069->1380316 1380296  [bsub-term-20+100.fasta] FW 31-79 14.64
TTCAATGAAAAAGCTTATCtcattacga---------------------GATAAGCT-TTTTTATACGCGA
>1381484->1381894 1381874  [bsub-term-20+100.fasta] FW 17-63 14.34
ATAATGACCGGCATCCTGAggattc------------------------TCAGGATGTTTTTTGGTGTCGG
>1386670->1387503 1387483  [bsub-term-20+100.fasta] FW 15-59 18.45
CAATAAAAAAATCCGCTATctgtg-------------------------ATAGCGGA-TTTTTTATGATGC
>1394241->1394753 1394733  [bsub-term-20+100.fasta] FW 16-57 21.80
ACTAATCAAAACCCCCGCTgc----------------------------AGCGGGGG-TTTTTCATGATAA
>1395479->1396834 1396814  [bsub-term-20+100.fasta] FW 18-67 17.84
```

```
TAAACGTAAAAAGTCCGTCtcaactgcga--------------------GCCGGACT-TTTATTATTTAAC
>1397962->1398426 1398406  [bsub-term-20+100.fasta] FW 18-64 14.51
TAACTATTCTACACTCTCTatttca------------------------GGAGAGTGTTTTTTTGTTTCCA
>1402945->1403958 1403938  [bsub-term-20+100.fasta] FW 19-65 15.90
AAACACGGAAAGAGCTGACttcatta-----------------------GTCAGCTC-TTTTTCTTCAACT
>1410120->1411094 1411074  [bsub-term-20+100.fasta] FW 17-63 16.02
GTAATCTGAAAGACTCTGCttaaaa------------------------GCAGAGTCTTTTTGTGTTTGAA
>1414463->1415359 1415339  [bsub-term-20+100.fasta] FW 25-70 16.07
AACACATTGTTCCTCTGAGagaatt------------------------CTCAGAGG-TTTTTTATTTTAT
>1415533->1416882 1416862  [bsub-term-20+100.fasta] FW 16-58 17.42
GATAGGCAAAACACCGCATatt---------------------------TTGCGGTG-TTTTTTGATTACC
>1429050->1429586 1429566  [bsub-term-20+100.fasta] FW 19-65 18.04
AAGCGTGAGATAGCCCCGTtcatcga-----------------------ACGGGGCT-TTTTATATTTATT
>1432665->1433102 1433082  [bsub-term-20+100.fasta] FW 14-67 17.52
TAAGTAAAAAAAGGAAGCCtgatatgtcacaa-----------------GGCTTCCTTTTTTGCTATTTTT
>1450104->1450601 1450581  [bsub-term-20+100.fasta] FW 9-55 14.35
ACGGAAGAATAGCTGAGAGcataga------------------------CTCTCAGCTTTTTTCATATAGA
>1450837->1452750 1452730  [bsub-term-20+100.fasta] FW 12-60 16.59
CTAAAATAAAACCAGCAGCctaatcag----------------------GCTGCTGGCTTTTTATGTATCA
>1459117->1460769 1460749  [bsub-term-20+100.fasta] FW 18-69 17.63
TAATGTACAAAAACCAGACggcctccggcct------------------GTCTGGTT-TTTTTCATAAGTA
>1461177->1462205 1462185  [bsub-term-20+100.fasta] FW 11-59 14.85
TTTCACTTAAACGGGCTGTtgtgatca----------------------ACAGCTCGCTTTTTTATAAAAA
>1463034->1464998 1464978  [bsub-term-20+100.fasta] FW 21-68 16.54
TCCAATTACATCCCCAAACatcattt-----------------------GTTTGGGGATTTTTTATTTTAT
>1465135->1466001 1465981  [bsub-term-20+100.fasta] FW 14-64 16.68
GAAATAAAAAAAGATTGCCtgtacgaaaca-------------------GGCAATCT-TTTTTTATTATTT
>1467207->1469264 1469244  [bsub-term-20+100.fasta] FW 15-68 15.27
AAATAAAAAACAGGGTGCAcaactaaaagattg----------------TGTGCCCT-TTCTTTTATTCAA
>1469428->1471248 1471228  [bsub-term-20+100.fasta] FW 13-56 16.38
AAAAATAAAAACAACGGCTtaaa--------------------------CGCCGTTG-TTTATCGTCTGCA
>1473004->1473915 1473895  [bsub-term-20+100.fasta] FW 17-68 17.89
ATAAATAAAAACAGCCGTTgccagaaagagg------------------CACGGCTG-TTTTTATTTTAAA
>1474549<-1475841 1474429  [bsub-term-20+100.fasta] FW 68-119 15.23
GAATGTAAAAATGCGCTTTtttctaagaaaa------------------AAAGCGCA-TTTTTAACCTGCA
>1478539->1480821 1480801  [bsub-term-20+100.fasta] FW 53-107 16.25
AAACTGCAAAACAAGCGAAcgttaaaactggtcg---------------TTCGCTTG-TTTTATTTTCTAT
>1481652->1482875 1482855  [bsub-term-20+100.fasta] FW 8-49 14.16
CGGACTGATTTAACGGCTGaa----------------------------AGGCCGTT-TTTTTTATGAAAT
>1485449->1486330 1486310  [bsub-term-20+100.fasta] FW 10-53 15.80
ATCCATTTTAAAGACAGCGagg---------------------------TGCTGTCTTTTTTTTATTTATC
>1488378->1489088 1489068  [bsub-term-20+100.fasta] FW 29-75 19.62
TTGATAAAAAAGGCGTGGAtaatata-----------------------TCCACGCC-TTATTCGTATAGG
>1490344->1490589 1490569  [bsub-term-20+100.fasta] FW 16-63 16.62
AGTAATAAAAAAACCGAAGcaaaaag-----------------------CTTCGGTTCTTTTATTTATTCT
>1493192->1493767 1493747  [bsub-term-20+100.fasta] FW 22-69 17.47
TATAAAGAAAAACTGCTTGgcatcgct----------------------CAAGCAGT-TTTTTTATTGAGG
>1498837->1499070 1499050  [bsub-term-20+100.fasta] FW 13-60 17.30
GGGGATGAAAACCGGACGGcatttcag----------------------CCGTCCGG-TTTTTCTGTATTT
>1505510->1506703 1506683  [bsub-term-20+100.fasta] FW 9-55 14.87
CGTTATGAGTAGCCTCATGcaacatg-----------------------CATGAGGT-TTTTTTATGTTTA
>1508661->1510568 1510548  [bsub-term-20+100.fasta] FW 17-65 18.62
ATAAGAAAAAAAGCGTCCTtggttcgaa---------------------AGGACGCT-TTTTCTTATCCGG
>1510713->1511294 1511274  [bsub-term-20+100.fasta] FW 11-65 15.16
AACAAAATAAAAACGCCTTgctggcctaggacag---------------CAGGCGTT-TTTATTTTGAAAA
>1511778->1513400 1513380  [bsub-term-20+100.fasta] FW 13-55 17.31
CCGAATAAAAAAGCAGAGAtt----------------------------TCTCTGCTTTTTTTGATACCTA
>1513457->1514368 1514348  [bsub-term-20+100.fasta] FW 13-58 17.44
AAAAATAAAAAAAGGAGGCggaaaa------------------------GCCTCCTT-TTATTTACTTAAA
>1517270->1517548 1517528  [bsub-term-20+100.fasta] FW 24-73 20.00
TATGCTAAAAAAGGCGGAGtgatatcat---------------------CTCCGCCTTTTTTGCGTGCCAA
>1519936->1520601 1520581  [bsub-term-20+100.fasta] FW 22-62 15.55
AGCCAAATAAGCCGTCCTCa-----------------------------AAGGGCGG-TTTTTCATATTAT
>1520756->1522489 1522469  [bsub-term-20+100.fasta] FW 13-62 17.26
TGCAATAAAAAGAGGCACTcccttaggg---------------------AGTGCCTCTTTTTTTAAACCTC
>1524791->1525564 1525544  [bsub-term-20+100.fasta] FW 15-59 18.41
AAATAAAGAAACGGCTGGCattgt-------------------------GCCAGCCG-TTTTCTCTTTTAG
>1526690->1527307 1527287  [bsub-term-20+100.fasta] FW 2-46 15.71
TCGTGTGAAAAAGAGCTGActcga-------------------------TCAGCTCT-TTTTTTGAATGAA
>1531275->1532687 1532667  [bsub-term-20+100.fasta] FW 24-73 18.60
TCATATCAAAAACAGCCCCgctttgagcg--------------------AGGGCTGT-TTTTTTATTTTGA
>1535341->1535607 1535587  [bsub-term-20+100.fasta] FW 16-61 21.25
TGTAAGAAAAAGAGCCGGGggatat------------------------CCCGGCTC-TTTTTTATGCTTG
>1536518->1536706 1536686  [bsub-term-20+100.fasta] FW 25-72 14.56
GCGATAGTGAAAACGAACCgcatgaat----------------------GGTTCGTT-TTTACGTGAGAAG
>1537669->1538097 1538077  [bsub-term-20+100.fasta] FW 49-93 15.83
TTCAATAAAAAGCCTCTCAaacat-------------------------TGAGAGGC-TTCTGATGATTTG
```

```
>1538175<-1539089 1538055  [bsub-term-20+100.fasta] FW 71-115 15.83
TTCAATAAAAAGCCTCTCAaacat-------------------------TGAGAGGC-TTCTGATGATTTG
>1544008->1544301 1544281  [bsub-term-20+100.fasta] FW 15-63 18.48
ATATAAAAAAAGCGCTTGTccgattccg--------------------GCAAGCGC-TTTTTTACATTAA
>1545225<-1545413 1545105  [bsub-term-20+100.fasta] FW 53-97 17.30
AGAAATGAAAAACACCGCTttctt-------------------------TGCGGTGT-TTTTTGATTACAA
>1547421->1547738 1547718  [bsub-term-20+100.fasta] FW 12-56 16.34
AAATCATAAAAGACTCAGCtgaaa------------------------GCTGAGTC-TTTTTTGTGTTCT
>1547794<-1548003 1547674  [bsub-term-20+100.fasta] FW 56-100 16.34
AAATCATAAAAGACTCAGCtgaaa------------------------GCTGAGTC-TTTTTTGTGTTCT
>1551817->1552098 1552078  [bsub-term-20+100.fasta] FW 18-66 15.68
TAAATGATAAAACTCAAACttattaaca--------------------GTTTGGGT-TTTTTTATAACCG
>1563827->1564720 1564700  [bsub-term-20+100.fasta] FW 12-58 15.95
ATATCATAAAAGGTACAGCgaaatat----------------------GCTGTACC-TTTTCGTTACATT
>1565783->1566823 1566803  [bsub-term-20+100.fasta] FW 13-63 14.05
GATGGTAATGAAAGCCGCGgacgaatggtt------------------CCCGGCTT-TTTCTATTTCCAT
>1575206->1575385 1575365  [bsub-term-20+100.fasta] FW 18-63 19.89
TAATGAGAAAAGCGCAGGGtgaaag-----------------------CCCTGCGC-TTTTTCTTTTTCT
>1575531->1576112 1576092  [bsub-term-20+100.fasta] FW 26-80 17.31
CGCCTGAAATGAACCGGCCctatagtaagaata---------------GGCCGGTTGTTTTGATTTCTAT
>1576169<-1576651 1576049  [bsub-term-20+100.fasta] FW 71-120 14.36
CCTGAAATGAACCGGCCCTatagtaagaa-------------------TAGGCCGG-TTGTTTTGATTTC
>1577778->1579397 1579377  [bsub-term-20+100.fasta] FW 26-68 15.23
AGTTTTAAAGAACCCTGACtag--------------------------TTCAGGGT-TTCTTTTTATGGG
>1581349->1583499 1583479  [bsub-term-20+100.fasta] FW 12-62 17.18
CCTGATTAAAAAGAAAGCCgtcgttatgca------------------GGCTTTCT-TTTTTTATGCCTT
>1588301->1589656 1589636  [bsub-term-20+100.fasta] FW 29-88 14.23
CTTGAAGTAAATCGGCAGCcctaatgactttaagcttgg---------GGTGTCGA-TTGCTTTGACTAC
>1592058->1592969 1592949  [bsub-term-20+100.fasta] FW 62-105 14.42
CAAACGAACAAACGGCATCatag-------------------------TATGCCGT-TTGTTTTGGAATA
>1598678->1602979 1602959  [bsub-term-20+100.fasta] FW 20-63 18.95
AGTGGAAAAAAAAGCTGCCGtcat------------------------TGGCAGCT-TTTTTTATATCCT
>1605025->1605807 1605787  [bsub-term-20+100.fasta] FW 16-67 15.63
AATAATGAAAAGCCTTTAAaacgatgttgtt-----------------TTAAAGGC-TTTTCTATTGATT
>1612758->1615523 1615503  [bsub-term-20+100.fasta] FW 18-63 21.81
TAATGCAAAAGAGCCCGGCgtaaaa-----------------------GCCGGGCT-TTTTTTGTGCCAA
>1628719->1629369 1629349  [bsub-term-20+100.fasta] FW 34-102 16.13
ATGATGTAAAGAGGCTTCAaagccttgctgtacttgaaaacaggctg--TGAGGCCTGTTTTTTTATTAAT
>1635002->1635490 1635470  [bsub-term-20+100.fasta] FW 10-57 16.93
GAAAGAAATAAGCCAGAGCatacaaat---------------------GCTCTGGC-TTTTCTTTATGCG
>1635530<-1637248 1635410  [bsub-term-20+100.fasta] FW 70-117 16.93
GAAAGAAATAAGCCAGAGCatacaaat---------------------GCTCTGGC-TTTTCTTTATGCG
>1654845->1654925 1654905  [bsub-term-20+100.fasta] FW 48-96 19.73
CCACATAAAAACGCCTGCCacatatcgg--------------------GGCAGGCG-TTCATTTTGTTAT
>1665109->1665858 1665838  [bsub-term-20+100.fasta] FW 12-58 16.42
AAACAATAAAATCCCCCTTatgact-----------------------CAGGGGGATTTCAGTATGTATG
>1669539->1670528 1670508  [bsub-term-20+100.fasta] FW 16-59 19.98
ATTAAGAAAAAGGCCCCAAcatc-------------------------TTGGGGCC-TTTTTCTTTTTTA
>1673291->1673536 1673516  [bsub-term-20+100.fasta] FW 12-57 15.09
TTTGACTAAAAGGGAGAGGgccgg------------------------CACCTCCCCTTTTTTTACACGC
>1675432->1675788 1675768  [bsub-term-20+100.fasta] FW 26-74 17.26
ACGAACGAAAAGAGCTTGTtacctgta---------------------ACAAGCTCTTTTTTTATACAGA
>1689518->1690297 1690277  [bsub-term-20+100.fasta] FW 18-62 17.94
TAATCACAAAAAGAACCCTttttg------------------------AGGGTTCT-TTTTTTATTTCAA
>1704938->1705207 1705187  [bsub-term-20+100.fasta] FW 1-69 14.72
TAAATCGTTTTGCAGGGTAatgtagagatgaattcaattattgactta-TTTCCTGC-TTTTTTATTGGTT
>1719918->1720475 1720455  [bsub-term-20+100.fasta] FW 44-90 15.91
ATAGTGAAAAGACCCTCTCatgttta----------------------CAGGGGGT-TTTTTTGTTAATA
>1724721->1726415 1726395  [bsub-term-20+100.fasta] FW 58-104 15.14
AAATAATAGAAGGTACCTCattgcct----------------------GAGGTACC-TTCACTTATGATG
>1735842->1736195 1736175  [bsub-term-20+100.fasta] FW 16-68 15.57
AATAAAAAAAGAGAGGATAggcgtatatcgtc----------------TGTCCTCT-TTCTTCGTTTATA
>1738332->1738601 1738581  [bsub-term-20+100.fasta] FW 19-58 18.56
AATCGTAAAAAGCGGGAGG-----------------------------ATTCCCGC-TTTTTTATCGTAT
>1742008->1743237 1743217  [bsub-term-20+100.fasta] FW 18-65 16.05
TAAAAAGGAAAGCCTGCCCcataatg----------------------GAGCAGGCATTTTTTAATCCCT
>1743315->1743572 1743552  [bsub-term-20+100.fasta] FW 56-101 17.27
AACATCTGAAACATCGGCTgccgg------------------------AGCCGATGTTTTTTTATATGGA
>1748929->1750476 1750456  [bsub-term-20+100.fasta] FW 23-64 19.45
ACTGACTAAAGACCGGAGCt----------------------------GCTCCGGTCTTTATTTTTTGTC
>1754174->1754899 1754879  [bsub-term-20+100.fasta] FW 12-57 15.90
CGATTCTAAAAACGGACGCctgtat-----------------------GTGTCTGT-TTTTTTATTTGCA
>1757725->1758972 1758952  [bsub-term-20+100.fasta] FW 16-61 16.50
CATAAACAAAACATCCCTCcagtgt-----------------------GAGGGGTG-TTTTTCTGCGGAA
>1764015->1765058 1765038  [bsub-term-20+100.fasta] FW 39-84 15.76
ATGATACAAAAGGCTGAGTgaaaa------------------------ACTCAGCTTTTTTGTATTTTGC
>1766678->1768240 1768220  [bsub-term-20+100.fasta] FW 10-54 15.72
```

```
ATGCAAAATAAAGTGATGCgctaa-------------------------GCATCACT-TTATTTTTTTGAC
>1770885->1772063 1772043  [bsub-term-20+100.fasta] FW 13-58 15.56
TATTGTAACAAGAGGAAGAgtttac------------------------TCTTCCTC-TTTTGATTCCTTA
>1773742->1774173 1774153  [bsub-term-20+100.fasta] FW 10-58 14.43
GTTCTCTCTAAACACGGTGcctttacag----------------------GCCCGTGT-TTTTTTATCATTT
>1781275->1781892 1781872  [bsub-term-20+100.fasta] FW 26-84 16.84
TAGTGTGAAAAGCGATTCTtgttgggcatgcgtccaaa-----------AGAGTCGC-TTTTTTATTTTCA
>1850132->1857763 1857743  [bsub-term-20+100.fasta] FW 21-68 19.66
GAAAACACAAACGCCCCCTcttttaaa----------------------AGGGGGCG-TTTTGAATGTTAT
>1860626<-1861954 1860506  [bsub-term-20+100.fasta] FW 66-120 14.88
AAACGAAAAAACCGGCAGTtctgctgagagaga----------------ATTGCCGGCTTTTGATGATGTT
>1863467->1863919 1863899  [bsub-term-20+100.fasta] FW 16-64 17.05
AATAAAGAAAACAGACGGTcactcacag---------------------GCCGTCTG-TTTTTTATCTTCA
>1871369->1871989 1871969  [bsub-term-20+100.fasta] FW 14-66 20.27
TGAATAAAAAAACCCGGTTtctcgcgatgagg-----------------AGCCGGGT-TTTTTTATGAGAC
>1872053<-1872820 1871933  [bsub-term-20+100.fasta] FW 50-102 20.27
TGAATAAAAAAACCCGGTTtctcgcgatgagg-----------------AGCCGGGT-TTTTTTATGAGAC
>1877669->1879003 1878983  [bsub-term-20+100.fasta] FW 18-68 17.06
TAATATCTCAATCCCTTGGcactaaaagtg-------------------TCAGGGGA-TTTTTTATGTTAA
>1881294->1882085 1882065  [bsub-term-20+100.fasta] FW 29-81 14.20
GTTGGTAACAAACATCCTCttacttattgtaa-----------------GAGGATGT-TTGTTTAATTCTT
>1897195<-1898379 1897075  [bsub-term-20+100.fasta] FW 20-64 15.09
TATGTTGAAAATCTCTCTTcttat-------------------------AGGAGGGA-TTTTTTTAGTTTC
>1897195<-1898379 1897075  [bsub-term-20+100.fasta] FW 67-107 14.62
AAAAAACAAAATCCCTCTGg-----------------------------TAGAGGGA-TTTTGATTCAGCA
>1902766<-1903518 1902646  [bsub-term-20+100.fasta] FW 73-117 15.96
TCCAAGGAAAACCGCCAGAcaat--------------------------GCTGGCGGCTTTTTGCTGCTTC
>1905064->1905462 1905442  [bsub-term-20+100.fasta] FW 53-93 16.30
AAAAACAAAAAAGAGAGAT------------------------------ACCTCTCTCTTTTTTATTCTTC
>1914476->1915234 1915214  [bsub-term-20+100.fasta] FW 14-62 14.91
CTACTGAAAAATAGGAGGCgataagaca---------------------GCCTCCTA-TTCCTTATGCTTC
>1916894<-1917511 1916774  [bsub-term-20+100.fasta] FW 54-103 18.86
TGAACAACAAACCCCCTCTccgtaaagga--------------------AGAGGGGG-TTATTTTATGCAT
>1918714->1918947 1918927  [bsub-term-20+100.fasta] FW 16-65 14.56
ACTAATATAAGAGGAATACggcaatatc---------------------GTATTCCTCTTTTGCATATACT
>1919116->1921119 1921099  [bsub-term-20+100.fasta] FW 20-65 14.70
AGCTTTTGAAAGAGGATGAgtcaaa------------------------TCATCCTC-TTTTTCTTGTTTA
>1923726->1924217 1924197  [bsub-term-20+100.fasta] FW 13-60 16.07
TGAATTAAAAAACCTTTCCtcttgtca----------------------GGAAAGGT-TTTTTATTTGAGA
>1929702->1929953 1929933  [bsub-term-20+100.fasta] FW 12-53 15.95
CGTTCTTAAAAAACCAGGGaa----------------------------ACCTGGTT-TTTTTCTTTCTGA
>1930470->1930769 1930749  [bsub-term-20+100.fasta] FW 15-59 19.81
TTGTAAAAAAAGAACGCACatgaa-------------------------GTGCGTTC-TTTTTTATTGGTA
>1934703->1937123 1937103  [bsub-term-20+100.fasta] FW 17-63 19.10
ATAATAAAAAACCCGCTCAtactata-----------------------TGAGCGGG-TTTCGTTTGTTTT
>1938180->1939577 1939557  [bsub-term-20+100.fasta] FW 27-75 16.87
TATAAAACAAAGCTGCATTcaatagttg---------------------AATGCAGC-TTTTTCATTATTG
>2001892->2002437 2002417  [bsub-term-20+100.fasta] FW 16-58 21.04
TATAAGAAAAACGCCCGGTata---------------------------AGCGGGCG-TTTTTTATTTGGT
>2003933->2005696 2005676  [bsub-term-20+100.fasta] FW 17-67 16.25
ATAAATAAAAAACTGTACTcgcttcaaatg-------------------AGTACAGT-TTTTTCATGCAGA
>2005797<-2006654 2005677  [bsub-term-20+100.fasta] FW 16-66 16.25
ATAAATAAAAAACTGTACTcgcttcaaatg-------------------AGTACAGT-TTTTTCATGCAGA
>2014035->2014937 2014917  [bsub-term-20+100.fasta] FW 18-72 15.25
TAAAAAAAATGAACCCGAGcttctatatagaagc---------------TTCGGGTT-TTTTTCTGCAAAT
>2014989<-2016104 2014869  [bsub-term-20+100.fasta] FW 66-120 15.25
TAAAAAAAATGAACCCGAGcttctatatagaagc---------------TTCGGGTT-TTTTTCTGCAAAT
>2018675->2018980 2018960  [bsub-term-20+100.fasta] FW 17-83 14.62
ATAAGGATGAATGAGATCCcgttgttacgatcagaatggacaaatg---GGATCTCA-TTTTTCGAGGAGG
>2028684<-2030288 2028564  [bsub-term-20+100.fasta] FW 68-113 15.00
AAAGTAGCCAACCCCCACCgaataa------------------------GATGGGGG-TTGCACGTAAGTA
>2031016->2032143 2032123  [bsub-term-20+100.fasta] FW 15-58 14.37
CATTGAAAAAACAGCGGGAaaag--------------------------ATCCGCTG-TTTCGTATTTTTT
>2049943->2050206 2050186  [bsub-term-20+100.fasta] FW 18-59 14.51
TAAACTTATAAAGGAGCTTtt----------------------------TAGCTCCT-TTTTCTCTTGTTT
>2056469->2056834 2056814  [bsub-term-20+100.fasta] FW 20-60 17.07
AGCAGATCCAAGCCCCCAT------------------------------ATGGGGGCGTTCTTATTATTGT
>2057056->2057715 2057695  [bsub-term-20+100.fasta] FW 35-100 14.14
TCGCTTTATATACCACCTCctcttagctaatatgttctaagtag-----GAGGTGGTATTTTGTTTGTATC
>2070541<-2070999 2070421  [bsub-term-20+100.fasta] FW 28-68 14.80
CAATAAAATAAGCTCTCATt-----------------------------GCGAGAGC-TTATTTTATTTAG
>2084558->2085235 2085215  [bsub-term-20+100.fasta] FW 34-77 14.57
TACTGACAAAAGCCCAAAAatga--------------------------ATTTGGGC-TTTTGCCATGTTT
>2085325<-2085861 2085205  [bsub-term-20+100.fasta] FW 44-87 14.57
TACTGACAAAAGCCCAAAAatga--------------------------ATTTGGGC-TTTTGCCATGTTT
>2090960->2091559 2091539  [bsub-term-20+100.fasta] FW 13-62 15.30
TTAAATAAAAAAGGATCTTggcatctgcc--------------------AGGATCCT-TTTTGTTAACCTG
```

```
>2098383->2098616 2098596  [bsub-term-20+100.fasta] FW 14-60 16.50
GGTGTAATAAGAACAGGGCgcacat------------------------GCCCTGTTCTTGTTTTTATATA
>2098702<-2099046 2098582  [bsub-term-20+100.fasta] FW 28-74 16.50
GGTGTAATAAGAACAGGGCgcacat------------------------GCCCTGTTCTTGTTTTTATATA
>2099403<-2099606 2099283  [bsub-term-20+100.fasta] FW 39-81 19.78
ACCACAAAAAAACCGCAGCtga---------------------------GCTGCGGT-TTTATGTATCGAC
>2099836->2101323 2101303  [bsub-term-20+100.fasta] FW 25-69 16.87
TATGTTTAAAAAACTGCTGcctgc-------------------------CAGCAGTT-TTTTTCTTTCTGT
>2105745->2106710 2106690  [bsub-term-20+100.fasta] FW 23-76 16.46
ATGCATCAAAAAGGGTACAtcactttatcgtga----------------TGTACCCT-TTTTTATTATCCT
>2125793->2126182 2126162  [bsub-term-20+100.fasta] FW 37-86 15.83
AAAAAAAAGAAGAGAGAGCtccaaagaa---------------------GCTCTCTCTTTTTTCATAAATG
>2127057->2127665 2127645  [bsub-term-20+100.fasta] FW 21-71 16.40
GGATATGAAAAACCTTAGTccgaaatccgg-------------------ACTAAGGT-TTTTTTATACAAT
>2129621->2131111 2131091  [bsub-term-20+100.fasta] FW 14-62 17.16
CGCATAAAAAAACCATACGcggcagccg---------------------CGTATGGT-TTTTTTTACATTT
>2133488->2133739 2133719  [bsub-term-20+100.fasta] FW 47-95 17.04
GACACAATGAAGCCCGCTTatctagtgt---------------------AAGCGGGC-TTTTCATTTTAAA
>2148327->2149280 2149260  [bsub-term-20+100.fasta] FW 14-58 15.06
TTCTTGAAAAAATCGTTCATtgct-------------------------ATGAACGATTTTTTTATTCATA
>2149351<-2150499 2149231  [bsub-term-20+100.fasta] FW 43-87 15.06
TTCTTGAAAAAATCGTTCATtgct-------------------------ATGAACGATTTTTTTATTCATA
>2169050->2169169 2169149  [bsub-term-20+100.fasta] FW 56-103 17.27
CTGTTCGATAGACGCCATAaaacttct----------------------TATGGCGT-TTTTTATTTTTAC
>2169273<-2169485 2169153  [bsub-term-20+100.fasta] FW 52-99 17.27
CTGTTCGATAGACGCCATAaaacttct----------------------TATGGCGT-TTTTTATTTTTAC
>2195227->2195505 2195485  [bsub-term-20+100.fasta] FW 16-59 14.37
ATTGATTTATCGAGGGGGTgtt---------------------------CCCCCCTCTTTCATTTTTAGGA
>2226746->2227963 2227943  [bsub-term-20+100.fasta] FW 10-56 14.14
TATTAAATTAAAGAAGGCTcctatta-----------------------GGCCTTCT-TTAATTAGGTTTA
>2245897<-2246583 2245777  [bsub-term-20+100.fasta] FW 4-71 14.27
ATTCAGCATAAAATCCCTCttttattcagaatcaagattcctcgttt--GAGGGATT-TTGCTTATGTATA
>2258716->2259537 2259517  [bsub-term-20+100.fasta] FW 10-49 14.20
AAACTGACTAAGATCCCCT------------------------------CCGGGATC-TTTTTTATTGGAG
>2271397->2271732 2271712  [bsub-term-20+100.fasta] FW 12-62 17.59
AATAAGTAAAATGCCCAGCtctttatttaa-------------------GCTGGGCA-TTGTTTTCATTAT
>2271775<-2272131 2271655  [bsub-term-20+100.fasta] FW 69-119 17.59
AATAAGTAAAATGCCCAGCtctttatttaa-------------------GCTGGGCA-TTGTTTTCATTAT
>2280122->2280604 2280584  [bsub-term-20+100.fasta] FW 11-52 15.67
AACAAAATAAAAAGCCCGTtt----------------------------TTGGGCTT-TTCTTTTTTTATA
>2287435->2287860 2287840  [bsub-term-20+100.fasta] FW 16-65 14.23
GGTGAAAAAAAAGCACATACcgatatatag-------------------GTATGTGC-TTTTCGCAGTGCT
>2287910<-2289247 2287790  [bsub-term-20+100.fasta] FW 66-115 14.23
GGTGAAAAAAAAGCACATACcgatatatag-------------------GTATGTGC-TTTTCGCAGTGCT
>2294543->2295184 2295164  [bsub-term-20+100.fasta] FW 23-67 20.74
GTAACAGAAAAGCGCAGTCttcac-------------------------GACTGCGC-TTTTTTATGCACG
>2305756->2306904 2306884  [bsub-term-20+100.fasta] FW 23-68 14.69
GCGTACTTGAGAGCATACGaaaat-------------------------CGTGTGCTCTTTTTATTTATAT
>2307737->2308006 2307986  [bsub-term-20+100.fasta] FW 11-54 17.31
CAAAGAATAAAAACCGCAGctt---------------------------CTGCGGTTTTTATTTTTAGTGA
>2325925->2326689 2326669  [bsub-term-20+100.fasta] FW 21-64 18.02
CAAATAAAAAAGCTGACGAacga--------------------------TCGTCAGC-TTTTTTCCATTAC
>2332026<-2332253 2331906  [bsub-term-20+100.fasta] FW 42-86 16.82
GCTTTAAAAAATGGGGCTCccgt--------------------------GGGCCCCATTTGTTGTGCTGCA
>2337824->2338012 2337992  [bsub-term-20+100.fasta] FW 39-83 14.16
AACGAAAAGAAGACAGCCTtttac-------------------------CGGCTGTC-TTCTCTTGCAATT
>2340686->2343430 2343410  [bsub-term-20+100.fasta] FW 18-64 18.01
TAAACAAAAAAGCCGTCACctttggg-----------------------GTGATGGC-TTTTTTGGTACAC
>2343506<-2344000 2343386  [bsub-term-20+100.fasta] FW 42-88 18.01
TAAACAAAAAAGCCGTCACctttggg-----------------------GTGATGGC-TTTTTTGGTACAC
>2345466<-2346758 2345346  [bsub-term-20+100.fasta] FW 66-110 15.21
TTACTGTAAAAAGTCTCCCttgc--------------------------AGGAGACTCTTTTTTGTTTCAT
>2362352<-2363119 2362232  [bsub-term-20+100.fasta] FW 46-86 14.39
CATATAAAAAAGCTGACGCc-----------------------------GGGTCAGC-TTTTGATAATAAA
>2384780<-2385058 2384660  [bsub-term-20+100.fasta] FW 33-73 16.45
AAATATGAAAAGCCCCTTAg-----------------------------AAAGGGGC-TTTTCTGTGAAGA
>2401304<-2402584 2401184  [bsub-term-20+100.fasta] FW 60-105 16.53
GAATAAAAAAATCTCCTATtttatc------------------------ATAGGAGA-TTTTTTATTTTTA
>2402740<-2403324 2402620  [bsub-term-20+100.fasta] FW 77-118 15.60
AACGAAAAAAAAACTGCTGAaa---------------------------TCAGCAGT-TTTCCGTTGCATT
>2408963->2409211 2409191  [bsub-term-20+100.fasta] FW 24-65 19.05
TAACTAAAAAAAGCTCCCGaa----------------------------CGGGAGCT-TTTTTATTTAATG
>2410320->2411897 2411877  [bsub-term-20+100.fasta] FW 18-63 18.50
TAATTAAAAAAAACTCAAGCtatata-----------------------GCTTGAGT-TTTTTTATTGTTC
>2411940<-2412707 2411820  [bsub-term-20+100.fasta] FW 75-120 18.50
TAATTAAAAAAAACTCAAGCtatata-----------------------GCTTGAGT-TTTTTTATTGTTC
>2414649<-2416418 2414529  [bsub-term-20+100.fasta] FW 46-90 19.76
```

```
TATTAAAAAAACACCCGCTcagtc-------------------------AGCGGGTG-TTTCTTATGGAAC
>2434245->2434457 2434437  [bsub-term-20+100.fasta] FW 57-100 15.49
GGTTTTGCCAACCGCCATCagca--------------------------TTCGGCGG-TTTTTTATTGAAC
>2434593<-2435024 2434473  [bsub-term-20+100.fasta] FW 21-64 15.49
GGTTTTGCCAACCGCCATCagca--------------------------TTCGGCGG-TTTTTTATTGAAC
>2434593<-2435024 2434473  [bsub-term-20+100.fasta] FW 74-119 16.49
CAATAAAAAAAGCTTCCTCtctaa-------------------------GAGGAAGCCTTTTGCCTGCCGA
>2435278->2436150 2436130  [bsub-term-20+100.fasta] FW 15-56 15.51
AAATAAGAAAAGCGCCGAAaa----------------------------ATCGGCGT-TTCTTTTATTGCT
>2458555->2459475 2459455  [bsub-term-20+100.fasta] FW 17-64 17.98
ATAGCAGAAAAACCAGCACctgtacgg----------------------GTGCTGGT-TTATTTATATTGA
>2459893->2460810 2460790  [bsub-term-20+100.fasta] FW 14-63 17.65
AGAATAAAAAAGCGAAAGGcctcttcggc--------------------TCTTTCGC-TTTTTTATGTTAC
>2472380->2473216 2473196  [bsub-term-20+100.fasta] FW 25-69 17.06
AAGGACAAAAACAGAAGGCacagt-------------------------GCCTTCTG-TTTTTTATTTTTC
>2475072->2476088 2476068  [bsub-term-20+100.fasta] FW 19-67 14.14
AATGCCCATAAGAGATATCctgtagag----------------------GATATCTCTTTTTTTATTTTTA
>2476198->2476959 2476939  [bsub-term-20+100.fasta] FW 20-69 19.55
ACGTAAAAAAAGACCGGGCcgtaagggat--------------------TCCCGGTC-TTTTATATTATTT
>2478385->2479854 2479834  [bsub-term-20+100.fasta] FW 23-74 18.33
AGAAGAAAAAAGCCGAAAAtgtttacaagca------------------TTTTCGGC-TTTTTTACGCTGA
>2483133->2483960 2483940  [bsub-term-20+100.fasta] FW 15-65 18.41
AAATAAAAAAGCAACCCCGtgcaaaaagc--------------------CGGGGTTGTTTTTTGTTACTTG
>2493888->2495057 2495037  [bsub-term-20+100.fasta] FW 16-70 17.93
CGTGATAAGAAGCGCATTCtttgtgtactgcaaa---------------GAATGCGC-TTCTTCTTTATAC
>2495130->2495969 2495949  [bsub-term-20+100.fasta] FW 24-84 15.22
AGCATAGAAAAAGAGCATTttttgaaacaaaacttcaaaa---------AATGCTCT-TTTTGCTTATTTA
>2506256->2506492 2506472  [bsub-term-20+100.fasta] FW 19-62 19.98
AGCTGAAAAAAAGCCGGAGaatg--------------------------CTCCGGCT-TTTTTTGTTGCAT
>2506534<-2507439 2506414  [bsub-term-20+100.fasta] FW 77-120 19.98
AGCTGAAAAAAAGCCGGAGaatg--------------------------CTCCGGCT-TTTTTTGTTGCAT
>2515667->2516785 2516765  [bsub-term-20+100.fasta] FW 18-65 17.19
TAATGTGCAAAGACTGCCGaaacgatt----------------------CGGCAGTC-TTTTTTCCCTTTA
>2531628<-2532284 2531508  [bsub-term-20+100.fasta] FW 74-118 18.91
GCAACGCAAAAAGCCCGCTtgttt-------------------------AGCGGGCT-TTTTTCCCTCATT
>2544271->2544651 2544631  [bsub-term-20+100.fasta] FW 16-61 15.57
AATAAAGCAAACAGCTGTCtggta-------------------------GACAGCTGTTTTTATTTATCTT
>2551929->2552264 2552244  [bsub-term-20+100.fasta] FW 53-110 16.40
CAATAACCATCGAGACGGCccagtatatgaatactgg------------GCCGTCTC-TTTTTTTTGCTGT
>2552357<-2553142 2552237  [bsub-term-20+100.fasta] FW 60-117 16.40
CAATAACCATCGAGACGGCccagtatatgaatactgg------------GCCGTCTC-TTTTTTTTGCTGT
>2554795->2555121 2555101  [bsub-term-20+100.fasta] FW 10-56 15.26
AATATCAATAAGGTCCTCCatttttt-----------------------GAAGGACC-TTATTCGTTTATT
>2563915->2564160 2564140  [bsub-term-20+100.fasta] FW 18-68 14.91
TAAATGCAGAAAGAGACAAaccttatcggt-------------------TTGTCTCT-TTTTTTACAATGA
>2564993->2565166 2565146  [bsub-term-20+100.fasta] FW 11-58 14.99
CGCACATTAAAACCTGTATccgatcgg----------------------ATACAGGT-TTATTTTTATGGC
>2565227<-2565511 2565107  [bsub-term-20+100.fasta] FW 50-97 14.99
CGCACATTAAAACCTGTATccgatcgg----------------------ATACAGGT-TTATTTTTATGGC
>2575993->2576457 2576437  [bsub-term-20+100.fasta] FW 15-55 18.35
CGATAAAAAAACAGGCTGAc-----------------------------TCAGCCTG-TTTTTTTCATCCA
>2588399->2589532 2589512  [bsub-term-20+100.fasta] FW 4-49 15.83
AAACACAAAAAGCTTGACGggaacc------------------------CGTCAAGC-TTTTTTGTGTTAG
>2592276->2592530 2592510  [bsub-term-20+100.fasta] FW 14-55 16.95
AAGCTAAAAAATGGGGGTGtt----------------------------ATCCCCCA-TTTTTTATTGCTG
>2595811->2596755 2596735  [bsub-term-20+100.fasta] FW 5-51 17.64
AAAAGCAAAAAACTGAGGCtgtaaaa-----------------------GCCTCAGT-TTTTTTATAGAAA
>2619983->2620915 2620895  [bsub-term-20+100.fasta] FW 15-60 15.97
AAATAAAAAAATCGGTACAttttaa------------------------TGCACCGA-TTTTTTAGGTTAA
>2635081->2635347 2635327  [bsub-term-20+100.fasta] FW 16-65 18.05
CATAATATAAAAACGATCCtcaataatgg--------------------GGATCGTT-TTTTTCATTTCTT
>2640480->2641301 2641281  [bsub-term-20+100.fasta] FW 66-113 15.37
CATTTTAAAAAACCATCGTtttaggaa----------------------ACGATGGT-TTTTGATTTCTGC
>2700607->2701023 2701003  [bsub-term-20+100.fasta] FW 19-64 16.05
AATGCAGAAGAAGCCGGATctcata------------------------CTCCGGCT-TTCTCTATTTGAA
>2718228<-2719532 2718108  [bsub-term-20+100.fasta] FW 37-88 14.71
TTGTTTAAAAAAGCCTGGAtgaaaatttcat------------------CTCAGGCT-TTAAGCATCTTAT
>2736184->2737227 2737207  [bsub-term-20+100.fasta] FW 34-82 14.72
ATAGAGCAAAAAGCAAACTttgacaggc---------------------AGTTTGCT-TTTTTTTTCGTAA
>2737577->2738374 2738354  [bsub-term-20+100.fasta] FW 57-100 14.52
TGAACTAAGAAGCGGGCTTaaa---------------------------AAGCCCACTTTTTTTCTAACAA
>2738755->2739462 2739442  [bsub-term-20+100.fasta] FW 65-107 16.46
ATAGTCTTAAAGGGGGCTTaac---------------------------ATGCCCCT-TTTTTCATTTCAA
>2748529->2748792 2748772  [bsub-term-20+100.fasta] FW 21-69 15.36
ACAAAACGGAAGCACTGATaaaaaata----------------------ATCAGTGCTTTTTATATGGCGA
>2756759->2757223 2757203  [bsub-term-20+100.fasta] FW 62-110 16.30
AATAACAAGAAGGCACAGActgttcggg---------------------TCTGTGCC-TTTTTTAAAATTA
```

```
>2765826->2767205 2767185  [bsub-term-20+100.fasta] FW 27-75 17.33
AATAAAAAAAAGACAAGGGatactcact---------------------CCCTTGTC-TTTTTATATGTGT
>2767312<-2767944 2767192  [bsub-term-20+100.fasta] FW 20-68 17.33
AATAAAAAAAAGACAAGGGatactcact---------------------CCCTTGTC-TTTTTATATGTGT
>2768097->2768924 2768904  [bsub-term-20+100.fasta] FW 35-84 17.20
TACCCCAAAAACAAGGCTTgtacaaaac---------------------AAGCCTTGTTTTTTTATGCATA
>2783440->2783922 2783902  [bsub-term-20+100.fasta] FW 17-64 18.24
ATAACAGAAAAACAGCCATctctgatg----------------------ATGGCTGT-TTTTTTATTCGTC
>2787950->2788153 2788133  [bsub-term-20+100.fasta] FW 17-63 19.33
CTAAAACAAAAGCAGCCGGttttaac-----------------------CCGGCTGC-TTTTGTTTATTTT
>2803927->2804583 2804563  [bsub-term-20+100.fasta] FW 14-60 14.27
AACATAGAAAAGGATACTCtttgga------------------------GAGTATCCTTTTTGCATTAAAA
>2811602->2812867 2812847  [bsub-term-20+100.fasta] FW 18-67 16.53
TAAAACAAAAAAACAGCCTtgatcatca---------------------AGGCTGTTTTTATGCATTATTT
>2817759->2819315 2819295  [bsub-term-20+100.fasta] FW 14-55 18.89
CCAATAAAAAAAGCTGCCTtt----------------------------TGGCAGCT-TTTTTTATTTTGA
>2822166<-2822678 2822046  [bsub-term-20+100.fasta] FW 68-112 14.22
AATAGGAAAAAAGCACCCTgacat-------------------------GGAGTGCT-TTTTCGTTATGCG
>2831176->2831628 2831608  [bsub-term-20+100.fasta] FW 14-59 21.05
CACATAAAAAAGAGCGGGCtcactt------------------------GCCCGCTC-TTTTTTGTTTGCT
>2831685<-2831954 2831565  [bsub-term-20+100.fasta] FW 57-102 21.05
CACATAAAAAAGAGCGGGCtcactt------------------------GCCCGCTC-TTTTTTGTTTGCT
>2839796->2840017 2839997  [bsub-term-20+100.fasta] FW 18-63 19.88
TAAATCAAAAAGGCAGCCGcacaag------------------------CGGCTGCC-TTTATCATGTATT
>2840871->2842325 2842305  [bsub-term-20+100.fasta] FW 19-71 15.37
GAACATACTAAAACCGGCCcgatatgacctcg-----------------TGCCGGTT-TTTTATGAACGAT
>2849976->2850512 2850492  [bsub-term-20+100.fasta] FW 21-60 17.88
AGACTAAAAAAAGCCCTCT------------------------------AGTGGGCT-TTTTTTATAATTG
>2878397->2878960 2878940  [bsub-term-20+100.fasta] FW 16-61 16.59
GATAAATAAAACCGGTAGAggatcc------------------------TCTACCGG-TTTATCATTTTTT
>2883881<-2885143 2883761  [bsub-term-20+100.fasta] FW 69-120 15.87
CATGCAAAAAAACCTCCTGagtgttaccact------------------CAGGAGGT-TTGTGCTTATCTT
>2904674<-2905435 2904554  [bsub-term-20+100.fasta] FW 77-120 14.57
GTTTAAAAAAACCTCTCCCatgc--------------------------GGAAGAGG-TTTTTTCTTACAT
>2908133->2908579 2908559  [bsub-term-20+100.fasta] FW 16-64 15.73
TGTAATCAAAAAGGCGGTGactgcatag---------------------TCCCGCCT-TTTTGATTGTCAT
>2908623<-2909849 2908503  [bsub-term-20+100.fasta] FW 72-120 15.73
TGTAATCAAAAAGGCGGTGactgcatag---------------------TCCCGCCT-TTTTGATTGTCAT
>2925133->2926074 2926054  [bsub-term-20+100.fasta] FW 16-61 16.34
CATAGAAAAAAGCTTGCAGatttct------------------------CTGCAAGC-TTTTTTATCAGCC
>2930794->2931009 2930989  [bsub-term-20+100.fasta] FW 18-61 19.53
TAAGACATGAAACCGGGTGacag--------------------------CGCCCGGT-TTTTTTCTTATAT
>2933696->2935030 2935010  [bsub-term-20+100.fasta] FW 13-64 15.05
CATCATGAGAAAGCCCAAAacagacattgt-------------------TTTGGGCTTTTGTGCGTTATTC
>2948191<-2949132 2948071  [bsub-term-20+100.fasta] FW 52-99 17.14
TCTTTTAAAAAAAGCCGGGggcagag-----------------------CCCGGCTTTTTATTCAATAGGA
>2952903->2953535 2953515  [bsub-term-20+100.fasta] FW 20-64 17.17
AAATAAAAAAAGCCAAGGCattca-------------------------GCCTTGGC-TTATCCTCCGATC
>2957509->2958291 2958271  [bsub-term-20+100.fasta] FW 15-64 17.35
AAATAAAAAAAGCATGATCtcttcaatga--------------------GATCATGC-TTTTTTATTTTAT
>2969112->2969954 2969934  [bsub-term-20+100.fasta] FW 21-65 17.18
TGGCCAAAAAACACCGTTCaatat-------------------------GAACGGTG-TTTTTTGTATTAT
>2982237->2983352 2983332  [bsub-term-20+100.fasta] FW 12-61 21.40
CAAAAATAAAACGGCAGCCgttttttcat--------------------GGCTGCCG-TTTTTTATTTGAT
>3016768<-3017271 3016648  [bsub-term-20+100.fasta] FW 76-120 14.47
CCATGAAAAAAGCTCCGGCggcag-------------------------CCTGGAGC-TTTTTTCCCTGCT
>3020305->3021108 3021088  [bsub-term-20+100.fasta] FW 19-63 17.21
AGAAAATAAAAGGACAGGCtgct--------------------------GCCTGTCCTTTTATGATTTCTC
>3027373->3028710 3028690  [bsub-term-20+100.fasta] FW 58-107 19.41
TAATAAAAAAAGAGCCTGCtcattacact--------------------GCGGGCTC-TTTTTCATGGTCG
>3028805<-3030493 3028685  [bsub-term-20+100.fasta] FW 63-112 19.41
TAATAAAAAAAGAGCCTGCtcattacact--------------------GCGGGCTC-TTTTTCATGGTCG
>3032497->3032733 3032713  [bsub-term-20+100.fasta] FW 16-66 20.43
AGTAATATAAAAGCCCAAAactgatatcgt-------------------TTTGGGCT-TTTTTTATTTTAT
>3034805->3035407 3035387  [bsub-term-20+100.fasta] FW 20-70 14.14
ATCGTTTTAAAAACCCCTGccgctatgcgg-------------------TCGGGGTT-TTTTTATCGGCTT
>3040467->3041630 3041610  [bsub-term-20+100.fasta] FW 14-57 15.87
AAAGTAAAAAACGACCTTTacga--------------------------AGAGGTCG-TTTTTGATTTTTT
>3051825<-3052430 3051705  [bsub-term-20+100.fasta] FW 45-92 15.65
GCAGAAACCAAGCCGCTAAacaagggc----------------------TTAGCGGC-TTTTCACATGATT
>3055561->3055878 3055858  [bsub-term-20+100.fasta] FW 32-78 19.01
CAAAAAATAGAAGCCGCTGcctatg------------------------CAGCGGCTCTTTTTTATTATAT
>3065533->3066468 3066448  [bsub-term-20+100.fasta] FW 15-64 17.15
ATTTAAAAAAAGGACTGGCttcagcagaa--------------------GCCGGTCC-TTTTATTTTGCAA
>3067990->3069288 3069268  [bsub-term-20+100.fasta] FW 24-67 17.26
TAGACCAAAAACCCAAACGtcgc--------------------------CGTTTGGG-TTTTTTATGTAAA
>3075898->3077436 3077416  [bsub-term-20+100.fasta] FW 17-66 14.66
```

```
TTAACAAAAAAGATCTTTCcgcgcctgcg--------------------GAAAGATC-TTTTTATTTGCGA
>3082522->3084840 3084820  [bsub-term-20+100.fasta] FW 13-63 16.25
GCATGTAAAAAACCCAAAAccgcttgcgcg-------------------TTTTGGGT-TTTTCCAATGTTA
>3092424<-3093770 3092304  [bsub-term-20+100.fasta] FW 29-95 14.46
CCTCTCTGGAACCGGCGCTccatccattgaccgcaggttacgatg----TACGCCGGCTTCTTTCATTCTG
>3094746<-3095645 3094626  [bsub-term-20+100.fasta] FW 65-112 16.45
GGAAACAAAAAGACCGTTTtcacaca-----------------------AAACGGTCTTTTTCGATTTCTT
>3099962->3101260 3101240  [bsub-term-20+100.fasta] FW 12-59 16.34
TACAAATAAAAACTAGGGGaccgctct----------------------CCCCTAGT-TTTTTGGTTTTGT
>3101710<-3104124 3101590  [bsub-term-20+100.fasta] FW 77-120 17.12
ATGAAAAAAAATCCCCTTTtggc--------------------------AAAGGGGA-TTTTTTCTAGGCT
>3107701->3108441 3108421  [bsub-term-20+100.fasta] FW 17-60 18.51
ATAAACAAAAAGCTCCAGAatg---------------------------TCTGGAGCTTTTTCTGTTTCAC
>3128611->3130194 3130174  [bsub-term-20+100.fasta] FW 19-66 15.16
AAAAAACAAAAGCCAAGAGcaattatg----------------------CTCTTGGC-TTGTTTTAATTGA
>3133224->3134036 3134016  [bsub-term-20+100.fasta] FW 14-55 22.53
GACATAAAAAACCCGGCACat----------------------------GTGCCGGG-TTTTTTATTCAAT
>3140975->3141142 3141122  [bsub-term-20+100.fasta] FW 12-63 15.28
TATAATTAAAAGAAAGAGCtgcatcagcca-------------------GCTCTTTCTTTTTGCATGTTGA
>3157039->3158262 3158242  [bsub-term-20+100.fasta] FW 68-113 14.24
ACTTAAAAAAAGGCATAAAtgtgac------------------------TTTATGCC-TTGCGCTTTACAG
>3162315->3162782 3162762  [bsub-term-20+100.fasta] FW 17-61 16.88
GTGAACAAAAAAAGCCGCTtatac-------------------------AGCGGCTT-TTTCACATCATCG
>3178376->3178954 3178934  [bsub-term-20+100.fasta] FW 19-68 19.29
AAAAGTAACAATCCCCCAGcgaattccg---------------------CTGGGGGATTTTTCGTTATCTG
>3185833->3186375 3186355  [bsub-term-20+100.fasta] FW 18-61 16.33
TAAAGCAGAAAACGCCTGGgaaa--------------------------CTAGGCGT-TTTTTTGATGTAA
>3186573->3187118 3187098  [bsub-term-20+100.fasta] FW 16-60 19.29
ACTAATGTAAAAGACCGGTtaac--------------------------GCCGGTCTTTTTTGCGTTTTAC
>3188159->3189496 3189476  [bsub-term-20+100.fasta] FW 21-64 19.94
TATCAAAAAAATCCGGCGTgcag--------------------------TCGCCGGA-TTTTTTATGATTC
>3195976->3196962 3196942  [bsub-term-20+100.fasta] FW 15-66 17.40
GCTTAAAAAAATCCGCCCGcgtgcaaatgcc------------------GCGGCGGA-TTTTTTATTAGAC
>3211661->3212398 3212378  [bsub-term-20+100.fasta] FW 14-66 14.47
CCGCTAAAAAGCCCCATCGcctattttccgga-----------------CGATGGGG-TTTCAAATGCCTT
>3218905<-3219309 3218785  [bsub-term-20+100.fasta] FW 42-92 16.37
CATACAAAAAAAGGAGGGGccgatgcttt--------------------CCCCTCCTCTTACATACAAGGT
>3223935->3224171 3224151  [bsub-term-20+100.fasta] FW 52-100 17.80
TGAAATCAAAAGCACCGAAcattcctg----------------------TCCGGTGCTTTTTTCATGCTTA
>3226658->3227479 3227459  [bsub-term-20+100.fasta] FW 17-57 18.54
ATAACAAAAAAACCGGTGCt-----------------------------GAACCGGT-TTTTTTAAGGCGT
>3227855->3229018 3228998  [bsub-term-20+100.fasta] FW 23-74 14.03
ATAGCTGTAAACGCCTTTAcgtcttcatttg------------------TAAAGGCG-TTCTTAATAAAGG
>3235501<-3236115 3235381  [bsub-term-20+100.fasta] FW 21-74 15.13
GAAGTTGTGAAACGGCGGAaacctgagaatcta----------------GCCGCCGT-TTCATTGCGTACA
>3237830->3238537 3238517  [bsub-term-20+100.fasta] FW 15-63 19.11
CTATAAGAAAACGCACTGCttgctttga---------------------GCAGTGCG-TTTTTCTGTCATA
>3243849->3245195 3245175  [bsub-term-20+100.fasta] FW 16-64 17.05
CATAAACAGAAAGAGTTCCgttttatgc---------------------GGAACTCT-TTTTTTATTTATT
>3251068->3251442 3251422  [bsub-term-20+100.fasta] FW 17-67 19.91
ATAAAAATAAGCAGCCGGAcaggcagagt--------------------TCCGGCTGTTTTTTTATTTCTT
>3252605<-3254914 3252485  [bsub-term-20+100.fasta] FW 71-115 15.26
TTTATAAAAAAGACTTGGCctgt--------------------------GCCAAGTCGTTTCCGTTATAAA
>3278870->3279307 3279287  [bsub-term-20+100.fasta] FW 20-64 15.37
AGATAGAAAAAGAGACTGCggcaa-------------------------GCAGTCTC-TTTTTGATAAGAT
>3293327->3293929 3293909  [bsub-term-20+100.fasta] FW 51-96 19.21
TAACGAGAAAAGGCTCCCCagtaaa------------------------GGGGAGCC-TTTTTCCTATAAA
>3293999<-3295327 3293879  [bsub-term-20+100.fasta] FW 41-84 17.02
ATCTACGTAAAAACCGTTCtatt--------------------------GGACGGTT-TTTTTATCATAAC
>3297134->3297610 3297590  [bsub-term-20+100.fasta] FW 14-63 19.23
CATGTAAAAAAAACGCTCCtgctttcgct--------------------GGAGCGTT-TTATTCTTTCACC
>3300643->3301641 3301621  [bsub-term-20+100.fasta] FW 16-69 16.37
AATAATAAAAAAAGGAGCTtgtgtctgcgacac----------------AGCTCCTT-TTTTATTTGAAGA
>3302099->3303079 3303059  [bsub-term-20+100.fasta] FW 19-79 16.14
AATCATAAAAAAACGCCAAgtcagcggttctccgcttgag---------TTGGCGTT-TTCTGCTACTTCT
>3303153<-3303776 3303033  [bsub-term-20+100.fasta] FW 45-105 16.14
AATCATAAAAAAACGCCAAgtcagcggttctccgcttgag---------TTGGCGTT-TTCTGCTACTTCT
>3309444->3309779 3309759  [bsub-term-20+100.fasta] FW 16-68 17.72
TTTAATAAAGCAGCCAGGCtgatatttgatca-----------------GCCTGGCT-TTTTTATTAGAGA
>3316560->3317060 3317040  [bsub-term-20+100.fasta] FW 9-55 15.49
CATACAGAATAAGGGCACCcttttag-----------------------GGCGCCCT-TTTTTCAAATGTA
>3320444->3321493 3321473  [bsub-term-20+100.fasta] FW 16-60 17.96
AGTAACAAAAAGCCGCCTCttcaa-------------------------CAGGCGGC-TTATTTGCTGCTT
>3333683->3334048 3334028  [bsub-term-20+100.fasta] FW 17-59 17.78
TTAAGAAGGAAGCCCGCCTcac---------------------------CGGCGGGC-TTCTTTTTGCACT
>3343172->3344038 3344018  [bsub-term-20+100.fasta] FW 16-62 17.98
CGTAAGAAAAAGGTGCTCCttttga------------------------GGAGCACCTTTTTCTAATACAC
```

```
>3353271->3353546 3353526  [bsub-term-20+100.fasta] FW 12-61 14.96
GAAGAGTAAAAAACCGCGGcccctcggc---------------------CAGCGGTTTTTCTTCTGCATAC
>3353610<-3353993 3353490  [bsub-term-20+100.fasta] FW 48-97 14.96
GAAGAGTAAAAAACCGCGGcccctcggc---------------------CAGCGGTTTTTCTTCTGCATAC
>3354652<-3356049 3354532  [bsub-term-20+100.fasta] FW 4-48 14.21
AAATAAAAGAAGACGAACTtcaaa-------------------------AGTTCGTC-TTCTACAATCAAA
>3374015->3375640 3375620  [bsub-term-20+100.fasta] FW 18-64 18.74
TAATAGAGCACCCCGCGGGtatcaa------------------------TCCGCGGGGTTTTTTATTCAAT
>3382624->3383085 3383065  [bsub-term-20+100.fasta] FW 17-63 17.88
GTAACAAAAAAGCTGAACCttaatcg-----------------------GGTTCAGC-TTTTTGTTTTTTC
>3383129<-3383803 3383009  [bsub-term-20+100.fasta] FW 73-119 17.88
GTAACAAAAAAGCTGAACCttaatcg-----------------------GGTTCAGC-TTTTTGTTTTTTC
>3385457->3386812 3386792  [bsub-term-20+100.fasta] FW 7-55 14.41
CAGTGCCAAAATAGACTGTagatgtttt----------------------GCAGTCTA-TTTTTTTATGTGA
>3387172->3388047 3388027  [bsub-term-20+100.fasta] FW 15-59 16.24
ATTTAGAAGAACGGCTGCTtaaaa-------------------------AGCAGCCG-TTCTTCCTATTAC
>3398150<-3399418 3398030  [bsub-term-20+100.fasta] FW 76-120 14.94
GCACAAAAAAACGCCATGCacgag-------------------------GCATGGCG-TTTTAAAACCCTT
>3399595<-3400176 3399475  [bsub-term-20+100.fasta] FW 68-117 17.36
AAACAAAAAAAGCCGGCATgtatttgaac--------------------ACGCCGGC-TTTCCTTTTCACT
>3403893->3404684 3404664  [bsub-term-20+100.fasta] FW 19-66 20.29
AGTATAAAAAAACGCATCCgtgtttc------------------------GGATGCGTTTTTTTTATACGTC
>3410746->3411156 3411136  [bsub-term-20+100.fasta] FW 15-63 15.64
AGCTAAAAAAAGAACGCATtccaattgg----------------------ATGCGTTC-TTTTTATTCATAG
>3417536->3418483 3418463  [bsub-term-20+100.fasta] FW 15-61 19.00
AAATAAACAAAAGAGCCGCctgccc------------------------GCGGCTCTTTTGCTTATTTATA
>3421416->3423269 3423249  [bsub-term-20+100.fasta] FW 13-58 16.41
CCGAATGAAAAAATCCCTCtatcaa------------------------GAGGGATT-TTTTACGTTTCCA
>3425220->3425780 3425760  [bsub-term-20+100.fasta] FW 13-57 16.59
CGCACTAAAAAAACTCCCCataac-------------------------GGGGAGTT-TTTTGATTAAAAC
>3426864->3427349 3427329  [bsub-term-20+100.fasta] FW 19-64 19.61
AATATAAAAAAAGCTGGCGctctaa------------------------CGCCAGCT-TTTTTTCTGCATT
>3427393<-3429405 3427273  [bsub-term-20+100.fasta] FW 75-120 19.61
AATATAAAAAAAGCTGGCGctctaa------------------------CGCCAGCT-TTTTTTCTGCATT
>3443391->3444467 3444447  [bsub-term-20+100.fasta] FW 17-62 20.77
CTAAAACAAAAGCCTCCCCaaataa------------------------GGGGAGGC-TTTTTCATTAGAA
>3447357<-3447758 3447237  [bsub-term-20+100.fasta] FW 52-97 14.48
CGATAAAAAAACAGAGACTtatac-------------------------CGTCTCTGCTTCATTACATCAG
>3457129->3458829 3458809  [bsub-term-20+100.fasta] FW 21-62 17.91
AGAGAAAAAAGAGGCTGGAc-----------------------------TCCAGCCTCTTTTTCTATTCTA
>3471689->3472303 3472283  [bsub-term-20+100.fasta] FW 16-71 14.47
ACTAGATAAAAAAAGACACagctgctatcgtgctc--------------GTGTCTTT-TTTTGTATGAATA
>3472435->3473133 3473113  [bsub-term-20+100.fasta] FW 16-63 16.57
TTTAAAAACAACCCCATCAcctgctgg----------------------TGATGGGG-TTGTTTTATTGAA
>3475106->3475573 3475553  [bsub-term-20+100.fasta] FW 22-71 18.58
AGCATAAAAAAGCCGCCGAgagaaaatc---------------------CCGGCGGTCTTTTTATTACTTG
>3483135<-3484529 3483015  [bsub-term-20+100.fasta] FW 69-117 15.63
CCCAATATAAACAGCCCTTcccgtaga----------------------AAGGGCTGTTTCATTAAAGCGT
>3484667->3485821 3485801  [bsub-term-20+100.fasta] FW 14-68 16.44
TGAATAAAAAAAGCAATGTatgggtctccccgct---------------ACATTGCT-TTTTTTATAGCTG
>3485870<-3486880 3485750  [bsub-term-20+100.fasta] FW 65-119 16.44
TGAATAAAAAAAGCAATGTatgggtctccccgct---------------ACATTGCT-TTTTTTATAGCTG
>3488015->3488932 3488912  [bsub-term-20+100.fasta] FW 16-65 17.34
AATAATAAAAACCCTCTTGccggatgcgg---------------------CAAGAGGG-TTTCTTATTTACT
>3499449->3500660 3500640  [bsub-term-20+100.fasta] FW 15-77 15.22
AAATAAAATGATCCATGAGacataacaatgatttgttacat--------CTCATGGATTTTTTTGTTTCAG
>3509843->3511534 3511514  [bsub-term-20+100.fasta] FW 13-59 16.91
TTCCTTAAAAAAATCCTCCcgtctag-----------------------GGAGGATT-TTATTTATATCGC
>3531385<-3531870 3531265  [bsub-term-20+100.fasta] FW 65-120 17.54
CAAAAAGAAAACCCGGAAAagagaagtaattctct--------------TTTCCGGG-TTTTTATGTTTAT
>3536567->3538117 3538097  [bsub-term-20+100.fasta] FW 13-61 19.82
ATGAATAAAAACAGGGGCGgcgcaggc----------------------TGCCCCTGTTTTTTTATTAGGA
>3538225->3539787 3539767  [bsub-term-20+100.fasta] FW 11-57 14.44
TGATAAATAAAGAAGCAAGaggtttt-----------------------CTTGCTTC-TTTATTCTTTACA
>3545294->3545887 3545867  [bsub-term-20+100.fasta] FW 16-69 17.61
AGTAATAACACAACCTGCAagagctgcgtctct----------------TGCAGGTT-TTTTTCATTTCAA
>3558692->3559012 3558992  [bsub-term-20+100.fasta] FW 13-55 20.77
ATGCATAAAAAAGCGGCCGcga---------------------------TGGCCGCT-TTTTTTAACCTGT
>3561626->3562603 3562583  [bsub-term-20+100.fasta] FW 18-63 17.75
TAAATCAAAAATCCGGTCTgataac------------------------AGACCGGA-TTTCATATTACAT
>3589663->3590328 3590308  [bsub-term-20+100.fasta] FW 19-65 17.40
AACAAAAAAAGTCCGCTGAtgttat------------------------TCAGCGGACTTTTTCAATCTTT
>3604583->3605794 3605774  [bsub-term-20+100.fasta] FW 13-56 19.60
ACATGTAAAAAAGCTGCCTttgc--------------------------GGGCAGCT-TTTTTTATTTTTT
>3614176<-3614406 3614056  [bsub-term-20+100.fasta] FW 53-104 15.79
GTGTATAAAAAACCTGCTTttaaaacgtaa-------------------AAGCAGGTATTCAGTCTTTATA
>3618024->3619358 3619338  [bsub-term-20+100.fasta] FW 16-78 16.84
```

```
CGTAACACAAAACAGCCCGgcatcgcatcatcctgcggtgc--------CGGGCTGCCTTTTATGATCCCG
>3646466->3647641 3647621  [bsub-term-20+100.fasta] FW 14-65 16.88
CTATTAAAAAATGCCCGGTccttttagagg------------------ATCGGGCATTTTTGCGCAGAAA
>3647714<-3648790 3647594  [bsub-term-20+100.fasta] FW 41-92 16.88
CTATTAAAAAATGCCCGGTccttttagagg------------------ATCGGGCATTTTTGCGCAGAAA
>3662341->3663261 3663241  [bsub-term-20+100.fasta] FW 18-68 16.09
TAAAACAAAAAGAAGCTTCgcacaatgtgc------------------AAAGCTTC-TTTTTTATTTGCC
>3664689->3665567 3665547  [bsub-term-20+100.fasta] FW 17-67 17.38
CTAAACAAAAAGGCTATTGgacattcatc-------------------CAATAGCCTTTTTTTATTTCAA
>3690429->3691553 3691533  [bsub-term-20+100.fasta] FW 20-65 20.51
AGCAATCAAAAGGGTGCGCatgatc-----------------------GCGCACCC-TTTTTTATGTTCC
>3693296->3694264 3694244  [bsub-term-20+100.fasta] FW 14-61 17.58
TGTATAATGAACGGGATGGcgattacg---------------------CCATCCCG-TTTTACATATTTC
>3694420->3695280 3695260  [bsub-term-20+100.fasta] FW 16-58 15.75
CATAAAGAAAAGACTCCAGaga--------------------------TTGGAGTC-TTTTTCTTTTATT
>3706201->3706737 3706717  [bsub-term-20+100.fasta] FW 16-65 15.17
AATAACTGAGAGGGATTCCgcataaatgc-------------------GGAATCCC-TTTTATTATGAAT
>3707856<-3708623 3707736  [bsub-term-20+100.fasta] FW 77-119 14.29
AATAAAAAGAAAGCCCTGCtaa--------------------------AAGGGGCT-TTCTTTTTTTCTT
>3711674->3713002 3712982  [bsub-term-20+100.fasta] FW 30-78 15.27
AAACGAAAAACTGCCTTCTgcttttca---------------------AGAGGGCAGTTTTGTATACACT
>3716295->3716882 3716862  [bsub-term-20+100.fasta] FW 31-84 15.39
CATTGTTTCAATAGGCTGCcggcggaggtacg----------------GAAGCCTATTTTTTTATTTGCC
>3721062->3721598 3721578  [bsub-term-20+100.fasta] FW 16-55 18.00
TATAGAAAAAAAGCACCTGG----------------------------ACAGGTGC-TTTTTTATTTTAG
>3734506->3737274 3737254  [bsub-term-20+100.fasta] FW 22-70 18.68
CATGACTAAAAAAGCTGGCtctaaaaga--------------------GCCAGCTT-TTTTCCGTTCATA
>3740788<-3741096 3740668  [bsub-term-20+100.fasta] FW 59-103 17.64
GCATGAAAAAAGCTGCCGTtttga------------------------ACGGCAGC-TTTTCTCTTCGTG
>3741440->3742276 3742256  [bsub-term-20+100.fasta] FW 32-75 17.14
GATAAAAAAAGACGCCGTTtct--------------------------AACGGCATCTTTTTTATTCCGG
>3748541->3749731 3749711  [bsub-term-20+100.fasta] FW 18-64 16.47
TAACTTGTCAGACTGCCGGgaaatc-----------------------CCGGCAGTCTTTTTTCCATTAA
>3749822<-3751228 3749702  [bsub-term-20+100.fasta] FW 27-73 16.47
TAACTTGTCAGACTGCCGGgaaatc-----------------------CCGGCAGTCTTTTTTCCATTAA
>3757601->3758182 3758162  [bsub-term-20+100.fasta] FW 21-72 17.28
GAAAGACAAAAGCCGGCGTctgaaatcaaga-----------------CAGCCGGC-TTTAATATTTCTC
>3761718->3763166 3763146  [bsub-term-20+100.fasta] FW 20-66 21.90
AGATGCGTAAACCCCCGGCctttacg----------------------GCCGGGGG-TTTTCCTGATGGT
>3764105->3764488 3764468  [bsub-term-20+100.fasta] FW 19-60 21.03
AATGAGAAAAACCCGGAGCtg---------------------------GCTCCGGG-TTTTTTATTATTC
>3779617->3780090 3780070  [bsub-term-20+100.fasta] FW 15-59 16.00
TCATAAAAAAATCCTTCTCataaa------------------------GAGAAGGA-TTTTTTCTTATTT
>3785675<-3785887 3785555  [bsub-term-20+100.fasta] FW 50-106 15.70
ACATAAAGCAATGGCGAAGgttctcttggaatgat-------------CTTCGCCATTTTTAGTTCATAT
>3797844->3798398 3798378  [bsub-term-20+100.fasta] FW 17-59 18.75
CTAAAGCAAAAAGCTCCCTtaa--------------------------AGGGAGCT-TTTTTTGTTACGC
>3801460<-3802047 3801340  [bsub-term-20+100.fasta] FW 59-106 14.98
ATATAAAAAAAACACCGGTgttttta----------------------GCCGGTGCATTCTATCTTTATA
>3809143->3809664 3809644  [bsub-term-20+100.fasta] FW 54-107 18.57
CAAATAAAAAAGACTTGCCcgcttttgacaaac---------------GGCAAGTC-TTTTTTATTACTT
>3817959->3818231 3818211  [bsub-term-20+100.fasta] FW 25-68 15.46
CATACAAAAAAGGATAGACatct-------------------------GTCTATCC-TTTTTCTTATGCT
>3829814->3830533 3830513  [bsub-term-20+100.fasta] FW 15-58 15.99
AGGTAAAAAAACAAGGACAccgc-------------------------TGTCCTTG-TTTTTTTCAGTCA
>3832703<-3834373 3832583  [bsub-term-20+100.fasta] FW 73-120 14.78
TCATAAAAAAAGGACAAAGcccgaaga---------------------CTTTGTCC-TTTTGATCGTGAT
>3846183->3846302 3846282  [bsub-term-20+100.fasta] FW 9-51 17.79
GGAATGATTTAACCGCCGTcca--------------------------TCGGCGGT-TTTTTCGTCCCCT
>3846401->3846874 3846854  [bsub-term-20+100.fasta] FW 15-60 16.87
GAATAAAAAAACATCCAGAcatcg------------------------TCTGGATGTTTACTTATTTCAC
>3852538->3852726 3852706  [bsub-term-20+100.fasta] FW 20-65 18.39
ATCTCTATAAAGCCGGCGCttcgc------------------------GCACCGGCTTTTATTATCCCTA
>3860236->3861012 3860992  [bsub-term-20+100.fasta] FW 20-69 15.01
ACATAGAAAAATCCCAAAAcgggcagctg-------------------TTTTGGGA-TTTTCGCCATGTG
>3865395->3866159 3866139  [bsub-term-20+100.fasta] FW 13-61 16.85
ATGTATAATATCCCCGCCCgccctatc---------------------CGGCGGGAGTTTTTCAATTCTC
>3866292->3867071 3867051  [bsub-term-20+100.fasta] FW 13-59 16.71
GCATATAAAAACATCCCGCtttttta----------------------GCGGGATGTTTCTTGTAATGAC
>3873131<-3874369 3873011  [bsub-term-20+100.fasta] FW 63-105 15.52
ACAAACAAAAAAACCCTTTaa---------------------------AAAGGGTTTTTAATTATAGGAA
>3877765<-3879312 3877645  [bsub-term-20+100.fasta] FW 65-116 15.61
AAAAGTTGAAATCGTGCGAtccgctaaagaa-----------------TCGCACGA-TTTTATTTCTCTC
>3892234->3892779 3892759  [bsub-term-20+100.fasta] FW 16-69 16.46
GATAAGAAAAAAGCCAATCactcatatgatgag---------------GATTGGCT-TTTTTGTTTATAG
>3892852<-3893193 3892732  [bsub-term-20+100.fasta] FW 43-96 16.46
GATAAGAAAAAAGCCAATCactcatatgatgag---------------GATTGGCT-TTTTTGTTTATAG
```

```
>3895081->3896454 3896434  [bsub-term-20+100.fasta] FW 13-55 16.20
TCAAATAAAAAAGCGCAGAtca---------------------------CCTGCGCT-TTTTACAAATCCT
>3899759->3900574 3900554  [bsub-term-20+100.fasta] FW 16-64 19.16
CATAAAAAAAGGGGCTGCCttacagaga---------------------GGCAGCCC-TTTTTAAATCACC
>3900664<-3900912 3900544  [bsub-term-20+100.fasta] FW 26-74 19.16
CATAAAAAAAGGGGCTGCCttacagaga---------------------GGCAGCCC-TTTTTAAATCACC
>3906640->3909060 3909040  [bsub-term-20+100.fasta] FW 16-60 18.34
AATAAGAAAAAGCCCTGCCgattc-------------------------GGCAGGGC-TTTTTAAAGATCA
>3912805->3913068 3913048  [bsub-term-20+100.fasta] FW 23-66 17.25
ACAGCAAAAAACCCTCTTCcact--------------------------GAAGAGGG-TTTTTTGTATTAT
>3917668->3917817 3917797  [bsub-term-20+100.fasta] FW 72-118 17.12
AATCGAAAAAAGCCTCAACcctagcg-----------------------GTTGAGGC-TTTTGACTGGTTT
>3923011->3924561 3924541  [bsub-term-20+100.fasta] FW 14-57 16.49
GGAATAAAAAAAGCTCTCTttat--------------------------CGAGAGCT-TTTTCCTTACTTT
>3934386->3934583 3934563  [bsub-term-20+100.fasta] FW 14-63 18.56
TACATAAAAAAAAGCTGCCcctgaacag---------------------GGCAGCTTTTTCATTATTTTTT
>3935929->3936309 3936289  [bsub-term-20+100.fasta] FW 17-66 17.52
GTAAAAATAAAGAACGTACattttgtgat--------------------GTACGTTC-TTTTTTATCTATA
>3938663->3940600 3940580  [bsub-term-20+100.fasta] FW 16-73 15.85
AGTAACCAAAAACCTTTAAgatttgcattccaagtc-------------TTAAAGGTTTTTTTCATTCTAA
>3942461->3943303 3943283  [bsub-term-20+100.fasta] FW 22-70 16.92
ACAAACAAAAAACGCTTTTgatcatctc---------------------AAAAGCGT-TTTTTTATCTGAT
>3943354<-3944214 3943234  [bsub-term-20+100.fasta] FW 71-119 16.92
ACAAACAAAAAACGCTTTTgatcatctc---------------------AAAAGCGT-TTTTTTATCTGAT
>3945952->3947193 3947173  [bsub-term-20+100.fasta] FW 25-74 15.28
ATATTCCGAAACGCTTATGacccttcatt--------------------CATAAGCG-TTTTTTTGCAGGT
>3947349->3948716 3948696  [bsub-term-20+100.fasta] FW 15-58 20.87
AAATAAAAAAAGACGGCACttgg--------------------------GTGCCGTC-TTTTTTAATCCAC
>3956185->3957276 3957256  [bsub-term-20+100.fasta] FW 16-62 22.82
AGTAAGAAAAAAGCCGGCCcattaca-----------------------GGCCGGCT-TTTTTTACGCTTC
>3963790->3965433 3965413  [bsub-term-20+100.fasta] FW 19-66 17.42
AACAACAGCGAACAGGGCTtttttaga----------------------AGCCCTGT-TTTTTTATTTTTC
>3985220->3986677 3986657  [bsub-term-20+100.fasta] FW 19-67 17.54
AAATGAAAAAATCCCTCTGtttcaagta---------------------CAGAGGGA-TTTTTCTTTATTT
>3988024->3988665 3988645  [bsub-term-20+100.fasta] FW 20-63 15.42
ACAGCTAACAAGGGTGCCTgttt--------------------------AGGCACCC-TTGTTCTTTAAAA
>3988740<-3989759 3988620  [bsub-term-20+100.fasta] FW 45-88 15.42
ACAGCTAACAAGGGTGCCTgttt--------------------------AGGCACCC-TTGTTCTTTAAAA
>3993867->3995099 3995079  [bsub-term-20+100.fasta] FW 18-63 19.39
TAACGCCAAAAGCCAGTCCaaaaaa------------------------GGACTGGC-TTTTTTGTGTGAA
>4010631<-4011359 4010511  [bsub-term-20+100.fasta] FW 70-118 17.89
TACAATCATAAGAGCCTGCtatattgca---------------------GCAGGCTC-TTTCACATTTGGC
>4030586<-4031032 4030466  [bsub-term-20+100.fasta] FW 81-120 18.61
AAATAAAAAAAAGCGCCCA------------------------------AGGGCGCT-TTTTTTACAAATC
>4040272->4040727 4040707  [bsub-term-20+100.fasta] FW 36-98 14.00
TTAGACGATAGGGGGCTATgcgtgaaaacagaagttcacagc-------ATAGCTCC-TTTTTGTATGGGC
>4046327->4047754 4047734  [bsub-term-20+100.fasta] FW 15-72 16.65
CTATAATTAAAAAAGCACAccccgctcagcatggga-------------TGTGCTTTATTTTTTATTCTGT
>4064999->4065352 4065332  [bsub-term-20+100.fasta] FW 18-70 17.65
TAACATATAAAAGGCTCAAcacaatgcgagtg-----------------TTGAGCCT-TTTTCCTTACTGT
>4065972->4066931 4066911  [bsub-term-20+100.fasta] FW 21-68 19.92
AAATAAAAAAACGGCACAGtcatgacg----------------------CTGTGCCG-TTTTTTATGATTC
>4084401->4085333 4085313  [bsub-term-20+100.fasta] FW 18-67 17.16
TAAGAAGAAAACAGCCTTCtccaatgga---------------------GAAGGCTGTTTTTTTGTGCGAT
>4086249->4086557 4086537  [bsub-term-20+100.fasta] FW 25-71 14.50
GTCTTAATCAAACCTTACTccgcgcg-----------------------GGTAAGGT-TTTTTTAATGGTT
>4086796->4088181 4088161  [bsub-term-20+100.fasta] FW 15-65 15.89
TTATAAAAAAAGGAATCGTctccttatgag-------------------ACGATTCC-TTTTTCTGTTTAC
>4090639->4091427 4091407  [bsub-term-20+100.fasta] FW 41-84 14.75
GAAACGAAATAAGAGAGGGagcc--------------------------CCCTCTCT-TTTTGTCTTTTAA
>4092773->4094110 4094090  [bsub-term-20+100.fasta] FW 24-70 18.07
CATGATCAAAAGGCGCTTCcttaga------------------------GGAGCGCCTTTTATTGTAACTC
>4099959->4101158 4101138  [bsub-term-20+100.fasta] FW 32-78 15.76
CACAGTAAGAAGACCTTCTtattaaa-----------------------AGAAGGTC-TTCTGCTATTCTA
>4101222<-4102274 4101102  [bsub-term-20+100.fasta] FW 68-114 15.76
CACAGTAAGAAGACCTTCTtattaaa-----------------------AGAAGGTC-TTCTGCTATTCTA
>4116288->4117694 4117674  [bsub-term-20+100.fasta] FW 22-72 17.29
CTGTATTAAAAACACGGTCagtttcaactg-------------------AACCGTGT-TTTTTTCTTCTAT
>4118735->4120264 4120244  [bsub-term-20+100.fasta] FW 19-76 16.25
AATATAAGAAATCCGCTATattgccagattggcagg-------------ATAGCGGATTTTTCTTTTTCTA
>4120374<-4121813 4120254  [bsub-term-20+100.fasta] FW 9-66 16.25
AATATAAGAAATCCGCTATattgccagattggcagg-------------ATAGCGGATTTTTCTTTTTCTA
>4134560->4134973 4134953  [bsub-term-20+100.fasta] FW 9-60 14.76
TACCAGCAATAAAGCCGGCgttctgagaacg------------------TCCGGCTT-TTTCTTTTACTTC
>4140567->4140683 4140663  [bsub-term-20+100.fasta] FW 18-60 19.09
TAAATGAAAAACCCCCGCGgga---------------------------TGCGGGGG-TTCAATTTAACGA
>4158213->4158413 4158393  [bsub-term-20+100.fasta] FW 20-61 15.90
```

```
ATGAATGAAAGCCTTCGCTgc----------------------------AGCGAGGG-TTTTTTAGCGGTT
>4158461<-4158664 4158341  [bsub-term-20+100.fasta] FW 72-113 15.90
ATGAATGAAAGCCTTCGCTgc----------------------------AGCGAGGG-TTTTTTAGCGGTT
>4158998->4160206 4160186  [bsub-term-20+100.fasta] FW 10-54 16.74
ATTCAGCATAAAGCGGGGAagata-------------------------TCTCCGCT-TTTTTCTTTGAAT
>4160311->4162368 4162348  [bsub-term-20+100.fasta] FW 15-62 17.07
GAATAAAAAAAGAGGCTTGgatgaatc----------------------CAAGCCTC-TTTTCTATTAAGC
>4167412->4168341 4168321  [bsub-term-20+100.fasta] FW 16-60 17.89
AATAAGCAAAAAGCATCCCgcgtc-------------------------GGGATGCT-TTTTCTTATTCAC
>4176108<-4176896 4175988  [bsub-term-20+100.fasta] FW 65-110 14.84
ACTGGAATAAAGAGTGCTTgctgaa------------------------AAGCACTC-TTTTTCTGCATCG
>4177499->4178338 4178318  [bsub-term-20+100.fasta] FW 11-57 14.57
TTGGAAATAAAAAAGGACCcgctaac-----------------------GGTCCTTT-TTTACTGATCAAT
>4181190->4181669 4181649  [bsub-term-20+100.fasta] FW 18-64 16.45
TAAATAGAAAAGGAGTGAGcctgaca-----------------------CTCACTCC-TTTTGCTCATGAA
>4185816->4186382 4186362  [bsub-term-20+100.fasta] FW 55-105 15.93
ATTGAACTAAAGGGGAATCcataaataatg-------------------GGTTCCCC-TTAATATACTATG
>4197811<-4198056 4197691  [bsub-term-20+100.fasta] FW 69-120 15.07
CCCTATAAAAAAGAGCAAGaacccgaaggtc------------------CTTGCTCT-TTACATATCAGCT
>4204108->4204725 4204705  [bsub-term-20+100.fasta] FW 17-63 16.22
ATAAAATAAAAACCATCTTcgtttga-----------------------AAGATGGT-TTTTTCATTTATG
>4206614->4207054 4207034  [bsub-term-20+100.fasta] FW 35-81 17.49
AAATATAAAAAGCTCTCCTgctttc------------------------AGGAGAGC-TTCTATTTTGGTA
```

File term_RNAmotif.txt (RNAMOTIF input file).

```
#Location: ~elesnik/E_coli_terminators/New_terminators/new_descr_results/descr9a_20final.descr
#Date: Jun 12, 2001
#Author: Elena Lesnik
#Manuscript:
http://nar.oxfordjournals.org/cgi/content/full/29/17/3583?ijkey=501381900c00a040709bce65d8ba536404d81cb1
#*********************************************************************************************************

parms
        wc+=gu;

descr
        ss(len=11)                                      #1 A-string
        h5(tag='1', minlen=4, maxlen=8) #2 5'-stem
        ss(minlen=3, maxlen=10)                 #3 loop
        h3 (tag='1')                            #4 3'-stem
        ss(minlen=0, maxlen=2, seq="^V*$")      #5 spacer
        ss(len=5, seq="^T")                     #6 T(1-5)-proximal part
        ss(len=3)                                       #7 T(6-9)-distal part
        ss(len=4)                                       #8 extra T's

        ### First bp is only GC,CG,or TA ####
sites
        h5 (tag='1', pos=1):h3 (tag='1', pos=$) in {"G:C","C:G","T:A","T:G"}

score
        {
                        a=0;
                        b=0;
                        c=0;
                        d=0;
                        e=0;
                        f=0;
                        g=0;

### Sugimoto parameters for hybrid duplexes (Biochemistry 1995,34,11211) #
                        p11=-1.0;      # rAA  /dTT (dG(kcal/mol)
                        p12=-2.1;      # rAC  /dTG
                        p13=-1.8;      # rAG  /dTC
                        p14=-0.9;      # rAU  /dTA
                        p21=-0.9;      # rCA  /dGT
                        p22=-2.1;      # rCC  /dGG
                        p23=-1.7;      # rCG  /dGC
                        p24=-0.9;      # rCU  /dGA
                        p31=-1.3;      # rGA  /dCT
                        p32=-2.7;      # rGC  /dCG
                        p33=-2.9;      # rGG  /dCC
                        p34=-1.1;      # rGU  /dCA
                        p41=-0.6;      # rUA  /dAT
                        p42=-1.5;      # rUC  /dAG
                        p43=-1.6;      # rUG  /dAC
                        p44=-0.2;      # rUU  /dAA


### At least four (GC/CG/GT/TG) bp should be in a stem ###
                    len2 = length (h5[2]);
                    len4 = length (h3[4]);
                for (i=1; i<=len2; i++){
                   if (h5[2,i,1]:h3[4,len2-i+1,1] in {"A:T","T:A"}){
                                a++ ;
                   }
                }
                        if (a > (len2-4))

                           REJECT;

                ### Restrictions for T stretch ###

        ## No less than three "T" in proximal part of T-stretch ##
                   len6 = length(ss[6]);
                  for (i=1; i <= len6; i++) {
                        y=ss[6,i,1];
                        if(y=~"T") {
                           b++;
                        }
                  }
```

```
                        if (b < 3)

                          REJECT;

        ## Calculate T's in distal part of T-region ##

                 len7 = length(ss[7]);
                for (j=1; j <= len7; j++) {
                        y=ss[7,j,1];
                        if (y=~"T") {
                         c++;
                        }
                 }
        ## If only 3"T"s in proxy-T additional 2"T"s should be in distal-T part ##
                        if ((b ==3) && (c < 2))
                                REJECT;

##If those strings in proxy-T part then five T"s are necessary ##
if (((ss[6]=~"^TVTTT$") || (ss[6]=~"^TTVTT$") || (ss[6]=~"^TTTVT$")) && (c < 2))

                                    REJECT;

        ## No more than 1 "G" in proximal T str ##
                   len6 = length(ss[6]);
                 for (i=2; i <= len6; i++) {
                        y=ss[6,i,1];
                        if (y=~"G") {
                            d++;
                        }
                  }
                        if (d >= 2)
                          REJECT;

        ## No more than 1 "C" in proximal T str ##
                   len6 = length(ss[6]);
                 for (i=2; i <= len6; i++) {
                        y=ss[6,i,1];
                        if (y=~"C") {
                            g++;
                        }
                  }
                        if (g >= 2)
                          REJECT;

        ## No V-strings in proximal T-str if it starts from "TVV" ##
                        if (ss[6]=~"^TVV")
                          REJECT;

        ## No four Pu or four "C" in distal T's ##

                if ((ss[7] =~"^RRRR$") || (ss[7]=~"^CCCC$"))
                        REJECT;

        {
            ### Scores for A-strings ###
                        A_score =0.0;

                 len1=length(ss[1]);
                for (i=1; i<=len1; i++){
                     y=ss[1,i,1];
                      if (y=~"A"){
                          e++;
                      }
                }
                        A_score = e;
        }

        {
          #  Score for a stem == number of b.p. in a stem ##
                        stem_score =0.0;
                for (i=1; i<=len2; i++) {
  if (h5[2,i,1]:h3[4,len2-i+1,1] in {"A:T","G:C","C:G","T:A","T:G","G:T"}) {
                          f++;
                     }
                }
                        stem_score = f;
        }
```

```
{
           ### Score for loops ###
               loop_score = 0.0;

             len3=length(ss[3]);
             loop_score = len3;
}

{
   ### Free energy and score=dG for a hairpin ###
                hairpin_score = 0.0;

                x=efn(h5[2], h3[4]);
                   hairpin_score = x;

                   if ( x > 0.0)
                        REJECT;
}

{
   ### Free energy and score=dG for a spacer ###
                spacer_score = 0.0;

                len5=length(ss[5]);
            if ((len5==1) && (ss[5] =~"^A$")){
                dG=p14;
                spacer_score =dG;
            }
            else if ((len5==1) && (ss[5] =~"^C$")){
                        dG=p24;
                        spacer_score =dG;
            }
            else if ((len5==1) && (ss[5] =~"^G$")){
                dG=p34;
                spacer_score =dG;
            }


            if ((len5==2) && (ss[5] =~"^AA$")){
                dG=p11+p14;
                spacer_score =dG;
            }
            else if ((len5==2) && (ss[5] =~"^AC$")){
                dG=p12+p24;
                spacer_score =dG;
            }
            else if ((len5==2) && (ss[5] =~"^AG$")){
                dG=p13+p34;
                spacer_score =dG;
            }


            else if ((len5==2) && (ss[5] =~"^CA$")){
                dG=p21+p14;
                spacer_score =dG;
            }
            else if ((len5==2) && (ss[5] =~"^CC$")){
                dG=p22+p24;
                spacer_score =dG;
            }
            else if ((len5==2) && (ss[5] =~"^CG$")){
                dG=p23+p34;
                spacer_score =dG;
            }


            else if ((len5==2) && (ss[5] =~"^GA$")){
                dG=p31+p14;
                spacer_score =dG;
            }
            else if ((len5==2) && (ss[5] =~"^GC$")){
                dG=p32+p24;
                spacer_score =dG;
            }
            else if ((len5==2) && (ss[5] =~"^GG$")){
                dG=p33+p34;
```

```
                spacer_score =dG;
            }
    }

    ### Free energy and score=dG for a proximal part of T-region ###
                proxy_T_score = 0.0;

                len6=length(ss[6]);
         for (i=1; i<=(len6-1); i++)    {
                y=ss[6,i,1];
                z = ss[6,i+1,1];
        if (y=~"T"){
              if(z=~"A") {
                    dG=p41;
                    proxy_T_score += dG;
               }
               else if(z=~"C") {
                    dG=p42;
                    proxy_T_score += dG;
               }
               else if(z=~"G") {
                    dG=p43;
                    proxy_T_score += dG;
               }
               else if(z=~"T") {
                    dG=p44;
                    proxy_T_score += dG;
               }
          }
          else if (y =~"A"){
               if(z =~"A") {
                    dG=p11;
                    proxy_T_score += dG;
               }
               else if(z =~"C") {
                    dG=p12;
                    proxy_T_score += dG;
               }
               else if(z =~"G") {
                    dG=p13;
                    proxy_T_score += dG;
               }
               else if(z=~"T") {
                    dG=p14;
                    proxy_T_score += dG;
               }
            }
            else if (y=~"C")  {
               if(z=~"A")   {
                    dG=p21;
                    proxy_T_score += dG;
               }
               else if(z=~"C") {
                    dG=p22;
                    proxy_T_score += dG;
               }
               else if(z=~"G") {
                    dG=p23;
                    proxy_T_score += dG;
               }
               else if(z=~"T") {
                    dG=p24;
                    proxy_T_score += dG;
               }
            }
            else if (y=~"G"){
               if(z=~"A") {
                    dG=p31;
                    proxy_T_score += dG;
               }
               else if(z=~"C") {
                    dG=p32;
                    proxy_T_score += dG;
               }
               else if(z=~"G") {
                    dG=p33;
                    proxy_T_score += dG;
```

```
                }
                else if(z=~"T") {
                     dG=p34;
                     proxy_T_score += dG;
                }
          }
       }
     }

     {

    ### Free energy and score=dG for distal part of T-region ###
                     dist_T_score = 0.0;

             len7=length(ss[7]);

      for (i=1; i<=(len7-1); i++)    {
              y=ss[7,i,1];
              z = ss[7,i+1,1];
           if (y=~"T")   {
                   if(z=~"A") {
                     dG=p41;
                     dist_T_score += dG;
                   }
                   else if(z=~"C") {
                     dG=p42;
                     dist_T_score += dG;
                   }
                   else if(z=~"G") {
                     dG=p43;
                     dist_T_score += dG;
                   }
                   if(z=~"T") {
                     dG=p44;
                     dist_T_score += dG;
                   }
            }
              else if (y=~"A"){
                   if(z=~"A") {
                     dG=p11;
                     dist_T_score += dG;
                   }
                   else if(z=~"C") {
                     dG=p12;
                     dist_T_score += dG;
                   }
                   else if(z=~"G") {
                     dG=p13;
                     dist_T_score += dG;
                   }
                   else if(z=~"T") {
                     dG=p14;
                     dist_T_score += dG;
                   }
            }
              else if (y=~"C"){
                   if(z=~"A") {
                     dG=p21;
                     dist_T_score += dG;
                   }
                   else if(z=~"C") {
                     dG=p22;
                     dist_T_score += dG;
                   }
                   else if(z=~"G") {
                     dG=p23;
                     dist_T_score += dG;
                   }
                   else if(z=~"T") {
                     dG=p24;
                     dist_T_score += dG;
                   }
             }
             else if (y=~"G"){
                    if(z=~"A") {
                      dG=p31;
                     dist_T_score += dG;
```

```
                  }
                  else if(z=~"C") {
                    dG=p32;
                   dist_T_score += dG;
                  }
                  else if(z=~"G") {
                    dG=p33;
                   dist_T_score += dG;
                  }
                  else if(z=~"T") {
                    dG=p34;
                   dist_T_score += dG;
                  }
           }
         }
   }

   {
     ### Free energy and score=dG for a extra T's ###
                   extra_T_score = 0.0;

                   len8=length(ss[8]);
    for (i=1; i<=(len8-1); i++)    {
            y=ss[8,i,1];
            z = ss[8,i+1,1];
         if (y=~"T")   {
                 if(z=~"A") {
                   dG=p41;
                   extra_T_score += dG;
                 }
                 else if(z=~"C") {
                   dG=p42;
                   extra_T_score += dG;
                  }
                  else if(z=~"G") {
                   dG=p43;
                   extra_T_score += dG;
                  }
                  else if(z=~"T") {
                   dG=p44;
                   extra_T_score += dG;
                  }
          }
            else if (y=~"A"){
                 if(z=~"A") {
                   dG=p11;
                   extra_T_score += dG;
                 }
                 else if(z=~"C") {
                   dG=p12;
                   extra_T_score += dG;
                 }
                 else if(z=~"G") {
                   dG=p13;
                   extra_T_score += dG;
                 }
                 else if(z=~"T") {
                   dG=p14;
                   extra_T_score += dG;
                 }
            }
                   else if (y=~"C")   {
                 if(z=~"A") {
                   dG=p21;
                   extra_T_score += dG;
                 }
                 else if(z=~"C") {
                   dG=p22;
                   extra_T_score += dG;
                 }
                 else if(z=~"G") {
                   dG=p23;
                   extra_T_score += dG;
                 }
                 else if(z=~"T") {
                   dG=p24;
                   extra_T_score += dG;
```

```
                    }
                }
                    else if (y=~"G"){
                  if(z=~"A") {
                    dG=p31;
                    extra_T_score += dG;
                  }
                  else if(z=~"C") {
                    dG=p32;
                    extra_T_score += dG;
                  }
                  else if(z=~"G") {
                    dG=p33;
                    extra_T_score += dG;
                  }
                  else if(z=~"T") {
                    dG=p34;
                    extra_T_score += dG;
                  }
                }
            }



### Combined dG_scores for 5 parts:dG_hairpin-[(dG_spacer)+(dG_proxy_T)+(dG_distal_T)+(dG_extra_T)]#

         dG_score = hairpin_score - spacer_score - proxy_T_score - (0.5 * dist_T_score) - (0.05 * extra_T_score);

                    if (dG_score >= -1.0)
                            REJECT;

## Combined struct_scores for 3 parts considering structural parameters: how far stem lengths are from max in experimental stem distribution (7bp)+ how far loop lengths are from experimental optimum (4 nt) and number of A's in A-string ##
                {
                        struct_score=0.0;
                 if (stem_score > 11.0)
                stem_dev =(11.0 - stem_score);
                   else if (stem_score <=11.0)
                        stem_dev = 0.0;
                }
                {
                if (loop_score > 6.0)
                loop_dev =(6.0 - loop_score);
                   else if (loop_score <=6.0)
                        loop_dev = 0.0;
                }
                {
               A_dev = 0.2 * (A_score);
                }

         struct_score = stem_dev + loop_dev + A_dev;

spacer = " |  ";

       {
    SCORE = sprintf('%s %3.2f %s %3.2f %s %3.2f %s %3.2f %s %3.2f %s %3.2f %s %3.1f %s %3.1f %s %3.1f %s %3.1f %s', spacer, hairpin_score, spacer, spacer_score, spacer, proxy_T_score, spacer, dist_T_score, spacer, extra_T_score, spacer, dG_score, spacer, stem_score, spacer, loop_score, spacer, A_score, spacer, struct_score, spacer);

        }
   }
```
