# Supplementary material for: Premature terminator analysis sheds light on a hidden world of bacterial transcriptional attenuation
Source: Genome Biol. 2010 Sep 29;11(9):R97. doi: 10.1186/gb-2010-11-9-r97 (PMC2965389; doi:10.1186/gb-2010-11-9-r97)
Supplement: Additional file 1 — Supplementary tables and figures. Table S0: gene families showing the highest absolute numbers of attenuator candidates. Table S1: genes most frequently regulated by attenuation in bacteria (normalized by family size). Table S2: list of sequence clusters observed in the 30 gene families most often regulated by attenuation (tabulation-separated). Table S3: sequence clusters obtained among candidates upstream of ABC-transporter genes. Table S4: complete list of clusters obtained by analyzing all candidates from enterobacterial species listed in Table S6. Cluster classes: 'a', clusters including only orthologous genes. 'b', clusters including only non-orthologous genes, sometimes from a single species; 'c', 'super-clusters' containing several sets of orthologous genes. Table S5: complete list of clusters obtained by analyzing all the candidates of Bacillus species listed in Table S6. 'a', clusters including only orthologous genes; 'b', clusters including only non-orthologous genes, sometimes from a single species; 'c', 'super-clusters' containing several sets of orthologous genes. Table S6: list of species analyzed for the identification of attenuators 'regulons'. Table S7: complete list of analyzed species, along with GenBank identifiers of corresponding DNA molecules and clade. Table S8: complete list of attenuators predicted in 5' UTR of genes, using the protocol described in [31] (tab-delimited table). Supplementary data 1: list of rimP-leaders from Gammaproteobacteria; list of rimP-leaders from other species; list of intergenic regions where no terminator could be detected, but showing sequence similarity to putative attenuators. Supplementary data 2: Stockholm alignments of the five ABC-leaders shown in Figure 4. Supplementary data 3: lists and Stockholm alignments of attenuator 'regulons' (candidates present upstream of several non-homologous genes) in Firmicutes. Supplementary data 4: parameters, commands and descriptor files used for terminator prediction. [file gb-2010-11-9-r97-S1.ZIP › Suppl_data/TableS0.pdf]

TableS0. Gene families showing the highest numbers of putative attenuators (in absolute values). Colored lines indicate families which do not appear in the normalized top list (see Supp. Table S1).

Hogenom family[1]	Candidates number[1]	Regulated gene (or one member of the regulated family)	Known attenuation system[2]
HBG543560	205	ssuB (ABC transporter ATP binding protein)	SAM riboswitch (25/205) / T-box (4/205)
HBG297802	131	rimP (first gene of the rimP-nusA-infB operon, encoding an hypothetical protein)	nusA attenuator (*E.coli*[3]) (1/131)
HBG369991	71	rplJ (50S ribosomal protein L10)	Ribosomal protein L10 leader (50/71)
HBG181500	69	thrS (threonyl-tRNA synthetase)	T-box (25/69) / Peptide leader (15/69)
HBG533752	66	fabI (short chain dehydrogenase reductase)	TPP riboswitch (1/66) / hdhA and ygcW attenuators (*E.coli*[3]) (2/66)
HBG529797	64	ompR (two component transcriptional regulator)	? (64)
HBG000748	59	pheS (phenylalanyl-tRNA synthetase)	T-box (27/59) / Peptide leader (10/59)
HBG379584	58	ileS (isoleucyl-tRNA synthetase)	T-box (40/58)
HBG453340	56	metR (transcriptional regulator LysR family)	? (56)
HBG343859	55	greA (transcription elongation factor GreA)	greA attenuator (*E.coli*[3]) (1/55)
HBG530048	50	rimL (ribosomal protein acetyltransferase)	T-box (1/50)
HBG142539	48	infC (translation initiation factor IF3)	Ribosomal protein L20 leader (3/48)
HBG409440	47	csp (cold shock protein)	RNA thermometer (?/47)
HBG507301	45	pyrR (pyrimidine regulatory protein PyrR)	PyrR binding site (24/45)
HBG439642	45	mdtA (multidrug resistance efflux pump)	? (45)
HBG529959	44	mcp (methyl-accepting chemotaxis sensory transducer)	? (44)
HBG398889	44	hisS (histidyl-tRNA synthetase)	T-box (11/44)
HBG491141	43	trxA (thioredoxin)	? (43)
HBG394055	42	gltJ (amino acid ABC transporter permease protein)	T-box (4/42) / TPP riboswitch (1/42) / gltJ attenuator (*E.coli*[3]) (1/42)
HBG461111	40	marR (MarR family transcriptional regulator)	? (40)
HBG379487	39	lepA (GTP binding protein LepA)	lepA attenuator (*E.coli*[3]) (1/39)
HBG460149	39	ilvB (acetolactate synthase large subunit)	Peptide leader (3/39)
HBG258034	39	pyrB (aspartate carbamoyltransferase)	PyrR binding site (7/39)
HBG445710	38	rocR (two component sigma54 specific transcriptional regulator Fis)	Lysine riboswitch (1/38)
HBG522524	38	mmuP (amino acid permease)	Lysine riboswitch (8/38) / T-box (1/38) / mmuP attenuator (*E.coli*[3]) (1/38)
HBG380413	38	metZ (O-succinylhomoserine sulfhydrylase)	SAM riboswitch (15/38)
HBG502043	38	aspC (aminotransferase)	? (38)
HBG536747	37	gloA (lactoglutathione lyase)	? (37)
HBG517301	37	rhlE (ATP-dependent RNA helicase)	? (37)
HBG519036	36	acsA (acetyl coA synthetase)	SAM riboswitch (1/36)
HBG507213	36	ykoY (integral membrane protein TerC)	yybP-ykoY leader (28/36)
HBG448785	34	trpE (anthranilate synthase)	T-box (4/34) / Peptide leader (22/34)
HBG046904	33	rpsL (30S ribosomal protein S12)	Ribosomal protein S12 leader (4/33)

HBG249606	33	tyrS (tyrosyl tRNA synthetase)	T-box (25/32)
HBG502884	32	transposase (IS1469, IS200, IS605, IS1541)	? (32)
HBG408484	31	transposase (IS660, ISBma2, ISPsy22)	? (31)
HBG528138	28	oppA (oligopeptide ABC transporter binding protein)	SAM riboswitch (6/28)
HBG401792	28	cysE (serine acetyltransferase)	T-box (16/28) / SAM riboswitch (1/28)
HBG408326	28	argD (aminotransferase)	? (28)
HBG473106	28	ycbK (putative membrane protein)	Glycine riboswitch (1/28)
HBG534647	28	proP (major facilitator family transporter)	? (28)
HBG465090	28	sigW (RNA polymerase sigma 24 factor)	? (28)
HBG460347	27	arsR (transcriptional regulator ArsR)	? (27)
HBG535696	27	transposase (IS3/IS911, IS407A, IS600, IS2, ISEhe3a, IS629, ISSdy1, IS1329)	? (27)
HBG032742	26	bcr (major facilitator family transporter)	? (26)
HBG456717	26	artJ (amino acid ABC transporter binding protein)	artJ attenuator (*E.coli*[3]) (1/26)
HBG426375	25	himD (DNA binding protein HU like)	ihfB attenuator (*E.coli*[3]) (1/25)
HBG114333	25	rpsP (30S ribosomal protein S16)	Ribosomal protein S16 leader (?/25)
HBG001285	25	rpoB (DNA directed RNA polymerase beta chain)	rplL attenuator (*E.coli*[3]) (1/25)
HBG444146	24	metK (S adenosylmethionine synthetase)	SAM riboswitch (15/24)
HBG519279	24	pyrP (xanthine/uracil permease)	PyrR binding site (14/24) / Purine riboswitch (2/24)
HBG439831	23	aldA (aldehyde dehydrogenase)	? (23)
HBG523338	23	pyrG (CTP synthase precursor)	Pyr regulatory elements (?/23)
HBG454398	23	adhA (alcohol dehydrogenase zinc binding protein)	? (23)
HBG416344	23	gst (glutathione S transferase)	ykoK leader (1/23)
HBG417191	23	fruR (LacI family transcriptional regulator)	? (23)
HBG000409	22	aroG (phospho-2-dehydro-3-deoxyheptonate aldolase)	? (21)
HBG053760	22	serS (seryl-tRNA synthetase)	T-box (18/22)
HBG491941	22	mgtA (cation transporting ATPase)	yybP-ykoY leader (10/22) / ykoK leader(8/22)
HBG457480	22	transposase (IS1181, ISSha1, IS1193)	? (22)
HBG521010	21	leuS (leucyl-tRNA synthetase)	T-box (9/21)
HBG533331	21	dnaX (DNA polymerase III subunit)	bacterial SRP (12/21)
HBG255791	21	pbuG (hypoxanthine/guanine permease)	Purine riboswitch (11/21) / ykkC-yxkD leader (1/21)
HBG411300	21	pheA (prephenate dehydratase)	Peptide leader (4/21)
HBG543577	20	ykkC (small multidrug resistance protein)	ykkC-yxkD leader (4/20)

[1] For each Hogenom gene family [4], the total number of detected attenuators is indicated, without any consideration on the size of this family. Families are sorted by decreasing scores.

[2] Numbers of known / predicted attenuators are indicated in parentheses. Predicted attenuators are considered as “known” if they match an entry of the RFAM database by a similarity search as described in the supplemental data.

[3] Attenuation systems found in the literature to be described at least in *E.coli*. The number '1' of known attenuators indicated in parentheses refers to the *E.coli* instance, but the exact number of described elements was not further evaluated.
