# Supplementary material for: Premature terminator analysis sheds light on a hidden world of bacterial transcriptional attenuation
Source: Genome Biol. 2010 Sep 29;11(9):R97. doi: 10.1186/gb-2010-11-9-r97 (PMC2965389; doi:10.1186/gb-2010-11-9-r97)
Supplement: Additional file 1 — Supplementary tables and figures. Table S0: gene families showing the highest absolute numbers of attenuator candidates. Table S1: genes most frequently regulated by attenuation in bacteria (normalized by family size). Table S2: list of sequence clusters observed in the 30 gene families most often regulated by attenuation (tabulation-separated). Table S3: sequence clusters obtained among candidates upstream of ABC-transporter genes. Table S4: complete list of clusters obtained by analyzing all candidates from enterobacterial species listed in Table S6. Cluster classes: 'a', clusters including only orthologous genes. 'b', clusters including only non-orthologous genes, sometimes from a single species; 'c', 'super-clusters' containing several sets of orthologous genes. Table S5: complete list of clusters obtained by analyzing all the candidates of Bacillus species listed in Table S6. 'a', clusters including only orthologous genes; 'b', clusters including only non-orthologous genes, sometimes from a single species; 'c', 'super-clusters' containing several sets of orthologous genes. Table S6: list of species analyzed for the identification of attenuators 'regulons'. Table S7: complete list of analyzed species, along with GenBank identifiers of corresponding DNA molecules and clade. Table S8: complete list of attenuators predicted in 5' UTR of genes, using the protocol described in [31] (tab-delimited table). Supplementary data 1: list of rimP-leaders from Gammaproteobacteria; list of rimP-leaders from other species; list of intergenic regions where no terminator could be detected, but showing sequence similarity to putative attenuators. Supplementary data 2: Stockholm alignments of the five ABC-leaders shown in Figure 4. Supplementary data 3: lists and Stockholm alignments of attenuator 'regulons' (candidates present upstream of several non-homologous genes) in Firmicutes. Supplementary data 4: parameters, commands and descriptor files used for terminator prediction. [file gb-2010-11-9-r97-S1.ZIP › Suppl_data/TableS1.pdf]

TableS1. Genes most frequently regulated by attenuation in bacteria.

**Hogenom family** [1]	**Score** [1]	**p-value**[5]	**Regulated gene**	**Known attenuation systems** [2]
HBG297802	131.6	< 2.2e-16	rimP (first gene of the rimP-nusA-infB operon, encoding an hypothetical protein)	nusA attenuator (*E.coli*[3]) (1/131)
HBG369991	72.3	< 2.2e-16	rplJ (50S ribosomal protein L10)	Ribosomal protein L10 leader (50/71)
HBG000748	66.9	< 2.2e-16	pheS (phenylalanyl-tRNA synthetase)	T-box (27/59) / Peptide leader (10/59)
HBG181500	65.6	< 2.2e-16	thrS (threonyl-tRNA synthetase)	T-box (25/69) / Peptide leader (15/69)
HBG142539	52.5	< 2.2e-16	infC (translation initiation factor IF3)	Ribosomal protein L20 leader (34/48)
HBG046904	35.3	< 2.2e-16	rpsL (30S ribosomal protein S12)	Ribosomal protein S12 leader (4/33)
HBG343859	35.1	< 2.2e-16	greA (transcription elongation factor GreA)	greA attenuator (*E.coli*[3]) (1/55)
HBG398889	31.0	< 2.2e-16	hisS (histidyl-tRNA synthetase)	T-box (11/44)
HBG448785	29.6	7.3e-16	trpE (anthranilate synthase)	T-box (4/34) / Peptide leader (22/34)
HBG249606	29.3	< 2.2e-16	tyrS (tyrosyl-tRNA synthetase)	T-box (25/32)
HBG444146	29.1	2.5e-11	metK (S adenosylmethionine synthetase)	SAM riboswitch (15/24)
HBG507301	28.3	< 2.2e-16	pyrR (pyrimidine regulatory protein PyrR)	PyrR binding site (24/45)
HBG379584	28.2	< 2.2e-16	ileS (isoleucyl-tRNA synthetase)	T-box (40/58)
HBG523338	28.1	9.3e-10	pyrG (CTP synthase precursor)	Pyr regulatory elements (?/23)
HBG114333	27.3	9.0e-11	rpsP (30S ribosomal protein S16)	Ribosomal protein S16 leader (?/25)
HBG001285	27.3	9.0e-11	rpoB (DNA directed RNA polymerase beta chain)	rplL attenuator (*E.coli*[3]) (1/25)
HBG401792	26.9	2.0e-13	cysE (serine acetyltransferase)	T-box (16/28) / SAM riboswitch (1/28)
HBG053760	26.5	4.8e-09	serS (seryl-tRNA synthetase)	T-box (18/22)
HBG262099	25.9	3.7e-08	leuA (2-isopropylmalate synthase)	Peptide leader (1/20)
HBG258034	25.2	3.1e-14	pyrB (aspartate carbamoyltransferase)	PyrR binding site (7/39)
HBG380413	24.7	1.1e-05	metZ (O-succinylhomoserine sulfhydrylase)	SAM riboswitch (15/38)
HBG507213	24.5	< 2.2e-16	ykoY (integral membrane protein TerC)	yybP-ykoY leader (28/36)
HBG000638	24.4	1.7e-07	carA (carbamoyl-phosphate synthase small subunit)	? (18)
HBG460149	23.5	3.5e-11	ilvB (acetolactate synthase large subunit)	Peptide leader (3/39)
HBG411300	23.1	2.5e-10	pheA (prephenate dehydratase)	Peptide leader (4/21)
HBG011746	22.5	9.2e-06	tsf (elongation factor Ts)	tsf attenuator (*E.coli*[3]) (1/17)
HBG098705	22.3	1.6e-15	ppiD (peptidylprolyl cis-trans isomérase D)	? (20)
HBG302075	21.2	6.7e-07	rbfA (ribosome-binding factor A)	rbfA attenuator (*E.coli*[3]) (1/19)
HBG372223	20.8	1.5e-06	uppS (undecaprenyl pyrophosphate synthetase)	? (19)

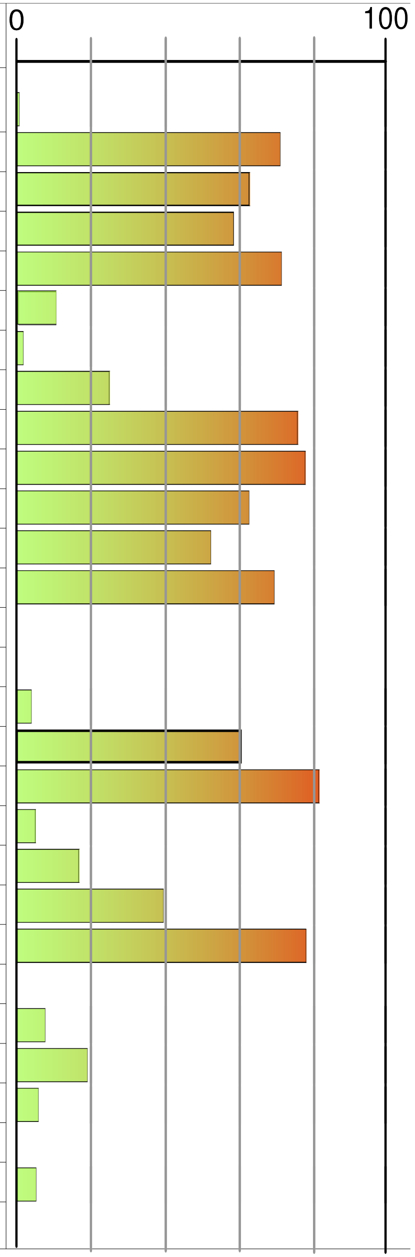

HBG409440	20.8	3.1e-13	csp (cold shock protein)	RNA thermometer (?/47)
HBG379487	20.8	2.5e-14	lepA (GTP binding protein LepA)	lepA attenuator (*E.coli*[3]) (1/39)
HBG009227	20.6	6.5e-08	hisG (ATP phosphoribosyltransferase)	Peptide leader (16/18)
HBG364928	20.3	1.1e-05	purA (adenylosuccinate synthetase)	? (14)
HBG146359	19.6	3.1e-04	rpsA (30S ribosomal protein S1)	? (14)
HBG489518	19.5	3.3e-04	ychF (GTP binfing protein YchF)	? (14)
HBG521010	19.4	2.9e-07	leuS (leucyl-tRNA synthetase)	T-box (9/21)
HBG413098	19.2	2.1e-05	ftsK (cell division protein FtsK)	? (28)
HBG519279	19.1	5.4e-10	pyrP (xanthine/uracil permease)	PyrR binding site (14/24) / Purine riboswitch (2/24)
HBG000124	19.0	1.0e-05	engA (GTP binding protein EngA)	der/engA attenuator (*E.coli*[3]) (1/16)
HBG001166	18.8	1.3e-06	glyQ (glycyl-tRNA synthetase α subunit)	? (15)
HBG450640	18.8	4.5e-06	argS (arginyl-tRNA synthetase)	T-box (8/18)
HBG517301	18.4	5.5e-07	rhlE (ATP-dependent RNA helicase)	? (37)
HBG386551	18.2	6.9e-09	kup (potassium transporter)	ydaO-yuaA leader (2/16)
HBG000409	18.1	2.5e-10	aroG (phospho-2-dehydro-3-deoxyheptonate aldolase)	? (21)
HBG416656	17.8	1.7e-03	mraZ (protein MraZ)	? (10)
HBG001137	17.7	1.5e-03	prfC (peptide chain release factor 3)	? (10)
HBG001503	17.6	1.3e-05	thiC (thiamine biosynthesis protein)	TPP riboswitch (14/14)
HBG288505	17.2	2.9e-03	gcvT (aminomethyltransferase)	Glycine riboswitch (8/10)
HBG529959	16.6	4.1e-04	mcp (methyl-accepting chemotaxis sensory transducer)	? (44)
HBG392570	16.6	1.9e-04	rplY (50S ribosomal protein L25)	? (12)
HBG249765	16.5	3.6e-03	rplU (50S ribosomal protein L21)	Ribosomal protein L21 leader (1/12)
HBG439642	16.5	5.4e-04	mdtA (multidrug resistance efflux pump)	? (45)
HBG536747	16.4	1.1e-08	gloA (lactoglutathione lyase)	? (37)
HBG000997	16.4	1.7e-04	aspS (aspartyl-tRNA synthetase)	? (15)
HBG347903	16.4	7.3e-05	alaS (aspartyl-tRNA synthetase)	T-box (8/16)
HBG278851	16.2	1.8e-04	glnB (nitrogen regulatory protein)	? (16)
HBG100060	16.1	1.1e-03	infA (translation initiation factor IF-1)	? (14)
HBG255791	15.9	9.9e-09	pbuG (hypoxanthine/guanine permease)	Purine riboswitch (11/21) / ykkC-yxkD leader (1/21)
HBG436112	15.8	1.4e-01	dnaJ (molecular chaperone DnaJ)	? (12)
HBG320298	15.8	6.7e-07	xpt (xanthine phosphoribosyltransferase)	Purine riboswitch (9/18)

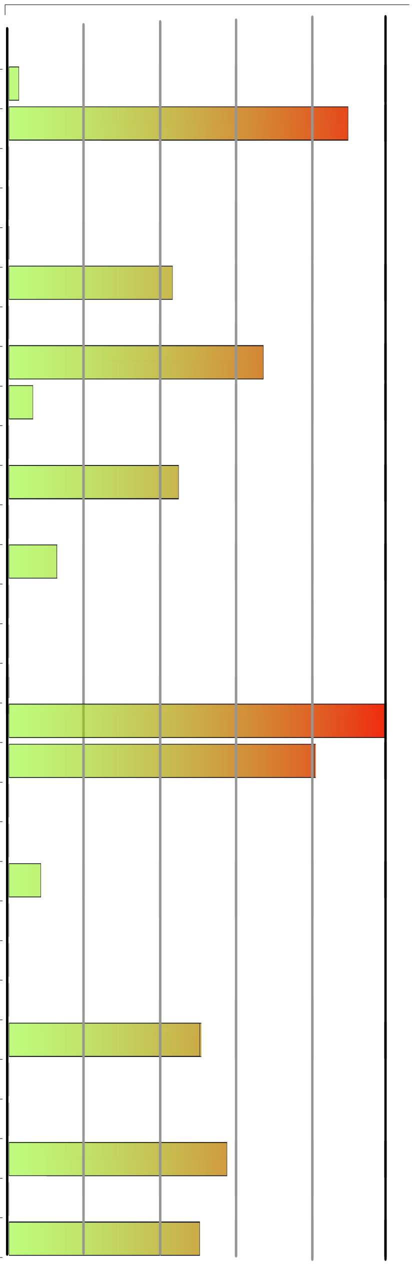

HBG221212	15.7	6.9e-02	dnaB (replicative DNA helicase)	dnaB attenuator (*E.coli*[3]) (0[4]/8)	
HBG003090	15.5	7.1e-04	trpS (tryptophanyl-tRNA synthetase)	T-box (4/14)	
HBG534647	15.4	4.7e-02	proP (major facilitator family transporter)	? (28)	
HBG458321	15.3	2.9e-01	mutT (mutator MutT)	mutT attenuator (*E.coli*[3]) (0[4]/15)	
HBG076716	15.2	2.8e-15	lp_1887 (probable transporter)	FMN riboswitch (16/16)	

1 For each Hogenom gene family [4] and species, the number of detected attenuators was normalized by the total number of genes in this family in the considered species. Final scores correspond to the sum of normalized scores from each species. Families are sorted by decreasing scores.

2 Numbers of known / predicted attenuators are indicated in parentheses. Predicted attenuators are considered as “known” if they match an entry of the RFAM database by a similarity search as described in the supplemental data.

3 Attenuation systems found in the literature to be described at least in *E.coli*. The number '1' of known attenuators indicated in parentheses refers to the *E.coli* instance, but the exact number of described elements was not further evaluated.

4 The *E.coli* attenuator indicated in RegulonDB lies within the preceding gene, and was thus missed by our screen.

5 p-value resulting from a Fisher's exact test for the considered Hogenom family, in consideration of the whole family size.
