# Supplementary material for: Premature terminator analysis sheds light on a hidden world of bacterial transcriptional attenuation
Source: Genome Biol. 2010 Sep 29;11(9):R97. doi: 10.1186/gb-2010-11-9-r97 (PMC2965389; doi:10.1186/gb-2010-11-9-r97)
Supplement: Additional file 1 — Supplementary tables and figures. Table S0: gene families showing the highest absolute numbers of attenuator candidates. Table S1: genes most frequently regulated by attenuation in bacteria (normalized by family size). Table S2: list of sequence clusters observed in the 30 gene families most often regulated by attenuation (tabulation-separated). Table S3: sequence clusters obtained among candidates upstream of ABC-transporter genes. Table S4: complete list of clusters obtained by analyzing all candidates from enterobacterial species listed in Table S6. Cluster classes: 'a', clusters including only orthologous genes. 'b', clusters including only non-orthologous genes, sometimes from a single species; 'c', 'super-clusters' containing several sets of orthologous genes. Table S5: complete list of clusters obtained by analyzing all the candidates of Bacillus species listed in Table S6. 'a', clusters including only orthologous genes; 'b', clusters including only non-orthologous genes, sometimes from a single species; 'c', 'super-clusters' containing several sets of orthologous genes. Table S6: list of species analyzed for the identification of attenuators 'regulons'. Table S7: complete list of analyzed species, along with GenBank identifiers of corresponding DNA molecules and clade. Table S8: complete list of attenuators predicted in 5' UTR of genes, using the protocol described in [31] (tab-delimited table). Supplementary data 1: list of rimP-leaders from Gammaproteobacteria; list of rimP-leaders from other species; list of intergenic regions where no terminator could be detected, but showing sequence similarity to putative attenuators. Supplementary data 2: Stockholm alignments of the five ABC-leaders shown in Figure 4. Supplementary data 3: lists and Stockholm alignments of attenuator 'regulons' (candidates present upstream of several non-homologous genes) in Firmicutes. Supplementary data 4: parameters, commands and descriptor files used for terminator prediction. [file gb-2010-11-9-r97-S1.ZIP › Suppl_data/TableS6.pdf]

TableS6. Species analysed for the identification of attenuators 'regulons'.

**Proteobacteria Gammaproteobacteria Enterobacteria**	
Photorhabdus luminescens subsp. laumondii TTO1	NC_005126
Erwinia carotovora subsp. atroseptica SCRI1043	NC_004547
Salmonella typhimurium LT2	NC_003197
Salmonella enterica subsp. enterica serovar Choleraesuis str. SC-B67	NC_006855
Shigella boydii Sb227	NC_007608
Shigella flexneri 2a str. 2457T	NC_004741
Shigella dysenteriae Sd197	NC_007606
Escherichia coli K12	NC_000913
Shigella sonnei Ss046	NC_007384
Sodalis glossinidius str. 'morsitans'	NC_007712
Yersinia enterocolitica subsp. enterocolitica 8081	NC_008791
Yersinia pestis CO92	NC_003134
Yersinia pseudotuberculosis IP 32953	NC_006153
**Firmicutes Bacilli**	
Bacillus clausii KSM-K16	NC_006582
Oceanobacillus iheyensis HTE831	NC_004193
Bacillus thuringiensis serovar konkukian str. 97-27	NC_005957
Bacillus anthracis str. Ames	NC_003997
Bacillus cereus ATCC 10987	NC_003909
Bacillus anthracis str. Sterne	NC_005945
Bacillus halodurans C-125	NC_002570
Bacillus licheniformis ATCC 14580	NC_006270
Bacillus subtilis subsp. subtilis str. 168	NC_000964
