# Supplementary material for: Premature terminator analysis sheds light on a hidden world of bacterial transcriptional attenuation
Source: Genome Biol. 2010 Sep 29;11(9):R97. doi: 10.1186/gb-2010-11-9-r97 (PMC2965389; doi:10.1186/gb-2010-11-9-r97)
Supplement: Additional file 1 — Supplementary tables and figures. Table S0: gene families showing the highest absolute numbers of attenuator candidates. Table S1: genes most frequently regulated by attenuation in bacteria (normalized by family size). Table S2: list of sequence clusters observed in the 30 gene families most often regulated by attenuation (tabulation-separated). Table S3: sequence clusters obtained among candidates upstream of ABC-transporter genes. Table S4: complete list of clusters obtained by analyzing all candidates from enterobacterial species listed in Table S6. Cluster classes: 'a', clusters including only orthologous genes. 'b', clusters including only non-orthologous genes, sometimes from a single species; 'c', 'super-clusters' containing several sets of orthologous genes. Table S5: complete list of clusters obtained by analyzing all the candidates of Bacillus species listed in Table S6. 'a', clusters including only orthologous genes; 'b', clusters including only non-orthologous genes, sometimes from a single species; 'c', 'super-clusters' containing several sets of orthologous genes. Table S6: list of species analyzed for the identification of attenuators 'regulons'. Table S7: complete list of analyzed species, along with GenBank identifiers of corresponding DNA molecules and clade. Table S8: complete list of attenuators predicted in 5' UTR of genes, using the protocol described in [31] (tab-delimited table). Supplementary data 1: list of rimP-leaders from Gammaproteobacteria; list of rimP-leaders from other species; list of intergenic regions where no terminator could be detected, but showing sequence similarity to putative attenuators. Supplementary data 2: Stockholm alignments of the five ABC-leaders shown in Figure 4. Supplementary data 3: lists and Stockholm alignments of attenuator 'regulons' (candidates present upstream of several non-homologous genes) in Firmicutes. Supplementary data 4: parameters, commands and descriptor files used for terminator prediction. [file gb-2010-11-9-r97-S1.ZIP › Suppl_data/TableS7.pdf]

| Species | Genbank ID | Classification |
|---|---|---|
| Methanothermobacter thermautotrophicus str. Delta H | NC_000916 | Archaea Methanobacteria |
| Rhodopirellula baltica SH 1 | NC_005027 | Planctomycetes |
| Salinibacter ruber DSM 13855 | NC_007677 | Bacteroidetes Sphingobacteria |
| Cytophaga hutchinsonii ATCC 33406 | NC_008255 | Bacteroidetes Sphingobacteria |
| Gramella forsetii KT0803 | NC_008571 | Bacteroidetes Flavobacteria |
| Porphyromonas gingivalis W83 | NC_002950 | Bacteroidetes Flavobacteria |
| Bacteroides thetaiotaomicron VPI-5482 | NC_004663 NC_004703 | Bacteroidetes Bacteroidetes |
| Bacteroides fragilis NCTC 9343 | NC_003228 | Bacteroidetes Bacteroidetes |
| Aquifex aeolicus VF5 | NC_000918 | Aquificae Aquificae |
| Thermotoga maritima MSB8 | NC_000853 | Thermotogae Thermotogae |
| Mesoplasma florum L1 | NC_006055 | Tenericutes Mollicutes |
| Mycoplasma mycoides subsp. mycoides SC str. PG1 | NC_005364 | Tenericutes Mollicutes |
| Mycoplasma capricolum subsp. capricolum ATCC 27343 | NC_007633 | Tenericutes Mollicutes |
| Mycoplasma gallisepticum R | NC_004829 | Tenericutes Mollicutes Mycoplasmatales |
| Mycoplasma genitalium G37 | NC_000908 | Tenericutes Mollicutes Mycoplasmatales |
| Mycoplasma penetrans HF-2 | NC_004432 | Tenericutes Mollicutes Mycoplasmatales |
| Mycoplasma pneumoniae M129 | NC_000912 | Tenericutes Mollicutes Mycoplasmatales |
| Ureaplasma parvum serovar 3 str. ATCC 700970 | NC_002162 | Tenericutes Mollicutes Mycoplasmatales |
| Mycoplasma pulmonis UAB CTIP | NC_002771 | Tenericutes Mollicutes Mycoplasmatales |
| Mycoplasma synoviae 53 | NC_007294 | Tenericutes Mollicutes Mycoplasmatales |
| Mycoplasma mobile 163K | NC_006908 | Tenericutes Mollicutes Mycoplasmatales |
| Mycoplasma hyopneumoniae 232 | NC_006360 | Tenericutes Mollicutes Mycoplasmatales |
| Onion yellows phytoplasma OY-M | NC_005303 | Tenericutes Mollicutes Acholeplasmatales |
| Aster yellows witches'-broom phytoplasma AYWB | NC_007716 | Tenericutes Mollicutes Acholeplasmatales |
| Candidatus Protochlamydia amoebophila UWE25 | NC_005861 | Chlamydiae Chlamydiae |
| Chlamydia muridarum Nigg | NC_002620 | Chlamydiae Chlamydiae |
| Chlamydia trachomatis A/HAR-13 | NC_007429 | Chlamydiae Chlamydiae |
| Chlamydophila pneumoniae AR39 | NC_002179 | Chlamydiae Chlamydiae |
| Chlamydophila felis Fe/C-56 | NC_007899 | Chlamydiae Chlamydiae |
| Chlamydophila abortus S26/3 | NC_004552 | Chlamydiae Chlamydiae |
| Chlamydophila caviae GPIC | NC_003361 | Chlamydiae Chlamydiae |
| Borrelia burgdorferi B31 | NC_001318 NC_001849 NC_001852 NC_001854 | Spirochaetes Spirochaetes |

| Species | Genbank ID | Classification |
|---|---|---|
| | NC_001857 | |
| Borrelia afzelii PKo | NC_008277 NC_008564 NC_008565 NC_008569 | Spirochaetes Spirochaetes |
| Borrelia garinii PBi | NC_006129 NC_006156 | Spirochaetes Spirochaetes |
| Treponema denticola ATCC 35405 | NC_002967 | Spirochaetes Spirochaetes |
| Treponema pallidum subsp. pallidum str. Nichols | NC_000919 | Spirochaetes Spirochaetes |
| Leptospira interrogans serovar Copenhageni str. Fiocruz L1-130 | NC_005823 | Spirochaetes Spirochaetes |
| Leptospira borgpetersenii serovar Hardjo-bovis JB197 | NC_008510 NC_008511 | Spirochaetes Spirochaetes |
| Thermus thermophilus HB27 | NC_005835 NC_005838 | Deinococcus-Thermus Deinococci |
| Deinococcus radiodurans R1 | NC_000958 NC_001263 NC_001264 | Deinococcus-Thermus Deinococci |
| Deinococcus geothermalis DSM 11300 | NC_008010 NC_008025 | Deinococcus-Thermus Deinococci |
| Fusobacterium nucleatum subsp. nucleatum ATCC 25586 | NC_003454 | Fusobacteria Fusobacteria |
| Chlorobium tepidum TLS | NC_002932 | Chlorobi Chlorobia |
| Pelodictyon luteolum DSM 273 | NC_007512 | Chlorobi Chlorobia |
| Chlorobium chlorochromatii CaD3 | NC_007514 | Chlorobi Chlorobia |
| Chlorobium phaeobacteroides DSM 266 | NC_008639 | Chlorobi Chlorobia |
| Campylobacter fetus subsp. fetus 82-40 | NC_008599 | Proteobacteria Epsilonproteobacteria |
| Campylobacter jejuni RM1221 | NC_003912 | Proteobacteria Epsilonproteobacteria |
| Wolinella succinogenes DSM 1740 | NC_005090 | Proteobacteria Epsilonproteobacteria |
| Helicobacter hepaticus ATCC 51449 | NC_004917 | Proteobacteria Epsilonproteobacteria |
| Helicobacter acinonychis str. Sheeba | NC_008229 | Proteobacteria Epsilonproteobacteria |
| Helicobacter pylori 26695 | NC_000915 | Proteobacteria Epsilonproteobacteria |
| Methylibium petroleiphilum PM1 | NC_008825 NC_008826 | Proteobacteria Betaproteobacteria |
| Rhodoferax ferrireducens T118 | NC_007901 NC_007908 | Proteobacteria Betaproteobacteria |
| Polaromonas naphthalenivorans CJ2 | NC_008757 NC_008759 NC_008761 NC_008762 NC_008764 NC_008781 | Proteobacteria Betaproteobacteria |
| Verminephrobacter eiseniae EF01-2 | NC_008771 NC_008786 | Proteobacteria Betaproteobacteria |
| Acidovorax avenae subsp. citrulli AAC00-1 | NC_008752 | Proteobacteria Betaproteobacteria |
| Chromobacterium violaceum ATCC 12472 | NC_005085 | Proteobacteria Betaproteobacteria |
| Neisseria gonorrhoeae FA 1090 | NC_002946 | Proteobacteria Betaproteobacteria |
| Neisseria meningitidis FAM18 | NC_008767 | Proteobacteria Betaproteobacteria |
| Nitrosospira multiformis ATCC 25196 | NC_007614 | Proteobacteria Betaproteobacteria |
| Nitrosomonas europaea ATCC 19718 | NC_004757 | Proteobacteria Betaproteobacteria |

| Species | Genbank ID | Classification |
|---|---|---|
| Nitrosomonas eutropha C91 | NC_008344 | Proteobacteria Betaproteobacteria |
| Bordetella pertussis Tohama I | NC_002929 | Proteobacteria Betaproteobacteria |
| Bordetella parapertussis 12822 | NC_002928 | Proteobacteria Betaproteobacteria |
| Bordetella bronchiseptica RB50 | NC_002927 | Proteobacteria Betaproteobacteria |
| Ralstonia solanacearum GMI1000 | NC_003295 NC_003296 | Proteobacteria Betaproteobacteria |
| Ralstonia metallidurans CH34 | NC_007971 NC_007972 NC_007973 NC_007974 | Proteobacteria Betaproteobacteria |
| Ralstonia eutropha H16 | NC_008313 NC_008314 | Proteobacteria Betaproteobacteria |
| Burkholderia xenovorans LB400 | NC_007951 NC_007952 NC_007953 | Proteobacteria Betaproteobacteria |
| Burkholderia cenocepacia AU 1054 | NC_008060 NC_008061 NC_008062 | Proteobacteria Betaproteobacteria |
| Burkholderia cepacia AMMD | NC_008385 NC_008390 NC_008391 NC_008392 | Proteobacteria Betaproteobacteria |
| Burkholderia thailandensis E264 | NC_007650 NC_007651 | Proteobacteria Betaproteobacteria |
| Burkholderia pseudomallei 1710b | NC_007434 NC_007435 | Proteobacteria Betaproteobacteria |
| Burkholderia mallei ATCC 23344 | NC_006348 NC_006349 | Proteobacteria Betaproteobacteria |
| Methylobacillus flagellatus KT | NC_007947 | Proteobacteria Betaproteobacteria |
| Thiobacillus denitrificans ATCC 25259 | NC_007404 | Proteobacteria Betaproteobacteria |
| Dechloromonas aromatica RCB | NC_007298 | Proteobacteria Betaproteobacteria |
| Azoarcus sp. EbN1 | NC_006513 NC_006823 NC_006824 | Proteobacteria Betaproteobacteria |
| Azoarcus sp. BH72 | NC_008702 | Proteobacteria Betaproteobacteria |
| Candidatus Ruthia magnifica str. Cm (Calyptogena magnifica) | NC_008610 | unclassified_Bacteria |
| Francisella tularensis subsp. holarctica | NC_007880 | Proteobacteria Gammaproteobacteria |
| Thiomicrospira denitrificans ATCC 33889 | NC_007575 | Proteobacteria Gammaproteobacteria |
| Thiomicrospira crunogena XCL-2 | NC_007520 | Proteobacteria Gammaproteobacteria |
| Buchnera aphidicola str. APS (Acyrthosiphon pisum) | NC_002528 | Proteobacteria Gammaproteobacteria |
| Haemophilus somnus 129PT | NC_008309 | Proteobacteria Gammaproteobacteria |
| Mannheimia succiniciproducens MBEL55E | NC_006300 | Proteobacteria Gammaproteobacteria |
| Actinobacillus pleuropneumoniae L20 | NC_009053 | Proteobacteria Gammaproteobacteria |
| Haemophilus ducreyi 35000HP | NC_002940 | Proteobacteria Gammaproteobacteria |
| Pasteurella multocida subsp. multocida str. Pm70 | NC_002663 | Proteobacteria Gammaproteobacteria |
| Haemophilus influenzae 86-028NP | NC_007146 | Proteobacteria Gammaproteobacteria |
| Psychromonas ingrahamii 37 | NC_008709 | Proteobacteria Gammaproteobacteria |
| Colwellia psychrerythraea 34H | NC_003910 | Proteobacteria Gammaproteobacteria |
| Idiomarina loihiensis L2TR | NC_006512 | Proteobacteria Gammaproteobacteria |

| Species | Genbank ID | Classification |
|---|---|---|
| Baumannia cicadellinicola str. Hc (Homalodisca coagulata) | NC_007984 | Proteobacteria Gammaproteobacteria |
| Candidatus Blochmannia floridanus | NC_005061 | Proteobacteria Gammaproteobacteria |
| Candidatus Blochmannia pennsylvanicus str. BPEN | NC_007292 | Proteobacteria Gammaproteobacteria |
| Pseudoalteromonas atlantica T6c | NC_008228 | Proteobacteria Gammaproteobacteria |
| Pseudoalteromonas haloplanktis TAC125 | NC_007481 NC_007482 | Proteobacteria Gammaproteobacteria |
| Photobacterium profundum SS9 | NC_005871 NC_006370 NC_006371 | Proteobacteria Gammaproteobacteria |
| Vibrio cholerae O1 biovar eltor str. N16961 | NC_002505 NC_002506 | Proteobacteria Gammaproteobacteria |
| Vibrio fischeri ES114 | NC_006840 NC_006841 NC_006842 | Proteobacteria Gammaproteobacteria |
| Vibrio parahaemolyticus RIMD 2210633 | NC_004603 NC_004605 | Proteobacteria Gammaproteobacteria |
| Vibrio vulnificus CMCP6 | NC_004459 NC_004460 | Proteobacteria Gammaproteobacteria |
| Photorhabdus luminescens subsp. laumondii TTO1 | NC_005126 | Proteobacteria Gammaproteobacteria |
| Erwinia carotovora subsp. atroseptica SCRI1043 | NC_004547 | Proteobacteria Gammaproteobacteria |
| Salmonella typhimurium LT2 | NC_003197 | Proteobacteria Gammaproteobacteria |
| Salmonella enterica subsp. enterica serovar Choleraesuis str. SC-B67 | NC_006855 NC_006856 NC_006905 | Proteobacteria Gammaproteobacteria |
| Shigella boydii Sb227 | NC_007608 NC_007613 | Proteobacteria Gammaproteobacteria |
| Shigella flexneri 2a str. 2457T | NC_004741 | Proteobacteria Gammaproteobacteria |
| Shigella dysenteriae Sd197 | NC_007606 NC_007607 | Proteobacteria Gammaproteobacteria |
| Escherichia coli K12 | NC_000913 | Proteobacteria Gammaproteobacteria |
| Shigella sonnei Ss046 | NC_007384 NC_007385 | Proteobacteria Gammaproteobacteria |
| Sodalis glossinidius str. 'morsitans' | NC_007712 NC_007713 | Proteobacteria Gammaproteobacteria |
| Yersinia enterocolitica subsp. enterocolitica 8081 | NC_008791 NC_008800 | Proteobacteria Gammaproteobacteria |
| Yersinia pestis CO92 | NC_003134 NC_003143 | Proteobacteria Gammaproteobacteria |
| Yersinia pseudotuberculosis IP 32953 | NC_006153 NC_006154 NC_006155 | Proteobacteria Gammaproteobacteria |
| Aeromonas hydrophila subsp. hydrophila ATCC 7966 | NC_008570 | Proteobacteria Gammaproteobacteria |
| Shewanella amazonensis SB2B | NC_008700 | Proteobacteria Gammaproteobacteria |
| Shewanella denitrificans OS217 | NC_007954 | Proteobacteria Gammaproteobacteria |
| Shewanella frigidimarina NCIMB 400 | NC_008345 | Proteobacteria Gammaproteobacteria |
| Shewanella baltica OS155 | NC_009035 NC_009038 NC_009052 | Proteobacteria Gammaproteobacteria |
| Shewanella oneidensis MR-1 | NC_004347 | Proteobacteria Gammaproteobacteria |
| Acinetobacter sp. ADP1 | NC_005966 | Proteobacteria Gammaproteobacteria |
| Psychrobacter arcticus 273-4 | NC_007204 | Proteobacteria Gammaproteobacteria |
| Psychrobacter cryohalolentis K5 | NC_007968 NC_007969 | Proteobacteria Gammaproteobacteria |

| Species | Genbank ID | Classification |
|---|---|---|
| Xanthomonas campestris pv. vesicatoria str. 85-10 | NC_007508 | Proteobacteria Gammaproteobacteria |
| Xanthomonas axonopodis pv. citri str. 306 | NC_003919 | Proteobacteria Gammaproteobacteria |
| Xanthomonas campestris pv. campestris str. 8004 | NC_007086 | Proteobacteria Gammaproteobacteria |
| Xanthomonas oryzae pv. oryzae KACC10331 | NC_006834 | Proteobacteria Gammaproteobacteria |
| Xanthomonas oryzae pv. oryzae MAFF 311018 | NC_007705 | Proteobacteria Gammaproteobacteria |
| Xylella fastidiosa Temecula1 | NC_004556 | Proteobacteria Gammaproteobacteria |
| Xylella fastidiosa 9a5c | NC_002488 | Proteobacteria Gammaproteobacteria |
| Legionella pneumophila str. Lens | NC_006369 | Proteobacteria Gammaproteobacteria |
| Legionella pneumophila subsp. pneumophila str. Philadelphia 1 | NC_002942 | Proteobacteria Gammaproteobacteria |
| Alcanivorax borkumensis SK2 | NC_008260 | Proteobacteria Gammaproteobacteria |
| Saccharophagus degradans 2-40 | NC_007912 | Proteobacteria Gammaproteobacteria |
| Marinobacter aquaeolei VT8 | NC_008738 NC_008739 NC_008740 | Proteobacteria Gammaproteobacteria |
| Pseudomonas aeruginosa PAO1 | NC_002516 | Proteobacteria Gammaproteobacteria |
| Pseudomonas fluorescens Pf-5 | NC_004129 | Proteobacteria Gammaproteobacteria |
| Pseudomonas syringae pv. phaseolicola 1448A | NC_005773 NC_005774 NC_005775 | Proteobacteria Gammaproteobacteria |
| Pseudomonas entomophila L48 | NC_008027 | Proteobacteria Gammaproteobacteria |
| Pseudomonas putida KT2440 | NC_002947 | Proteobacteria Gammaproteobacteria |
| Hahella chejuensis KCTC 2396 | NC_007645 | Proteobacteria Gammaproteobacteria |
| Chromohalobacter salexigens DSM 3043 | NC_007963 | Proteobacteria Gammaproteobacteria |
| Coxiella burnetii RSA 493 | NC_002971 NC_004704 | Proteobacteria Gammaproteobacteria |
| Halorhodospira halophila SL1 | NC_008789 | Proteobacteria Gammaproteobacteria |
| Alkalilimnicola ehrlichei MLHE-1 | NC_008340 | Proteobacteria Gammaproteobacteria |
| Methylococcus capsulatus str. Bath | NC_002977 | Proteobacteria Gammaproteobacteria |
| Nitrosococcus oceani ATCC 19707 | NC_007483 NC_007484 | Proteobacteria Gammaproteobacteria |
| Candidatus Pelagibacter ubique HTCC1062 | NC_007205 | Proteobacteria Gammaproteobacteria |
| Neorickettsia sennetsu str. Miyayama | NC_007798 | Proteobacteria Alphaproteobacteria |
| Wolbachia endosymbiont of Drosophila melanogaster | NC_002978 | Proteobacteria Alphaproteobacteria |
| Ehrlichia canis str. Jake | NC_007354 | Proteobacteria Alphaproteobacteria |
| Ehrlichia chaffeensis str. Arkansas | NC_007799 | Proteobacteria Alphaproteobacteria |
| Anaplasma marginale str. St. Maries | NC_004842 | Proteobacteria Alphaproteobacteria |
| Anaplasma phagocytophilum HZ | NC_007797 | Proteobacteria Alphaproteobacteria |
| Magnetococcus sp. MC-1 | NC_008576 | Proteobacteria Alphaproteobacteria |

| Species | Genbank ID | Classification |
|---|---|---|
| Rickettsia felis URRWXCal2 | NC_007109 | Proteobacteria Alphaproteobacteria |
| Rickettsia conorii str. Malish 7 | NC_003103 | Proteobacteria Alphaproteobacteria |
| Rickettsia bellii RML369-C | NC_007940 | Proteobacteria Alphaproteobacteria |
| Rickettsia typhi str. Wilmington | NC_006142 | Proteobacteria Alphaproteobacteria |
| Granulibacter bethesdensis CGDNIH1 | NC_008343 | Proteobacteria Alphaproteobacteria |
| Gluconobacter oxydans 621H | NC_006672 NC_006673 NC_006677 | Proteobacteria Alphaproteobacteria |
| Zymomonas mobilis subsp. mobilis ZM4 | NC_006526 | Proteobacteria Alphaproteobacteria |
| Novosphingobium aromaticivorans DSM 12444 | NC_007794 | Proteobacteria Alphaproteobacteria |
| Erythrobacter litoralis HTCC2594 | NC_007722 | Proteobacteria Alphaproteobacteria |
| Sphingopyxis alaskensis RB2256 | NC_008048 | Proteobacteria Alphaproteobacteria |
| Rhodospirillum rubrum ATCC 11170 | NC_007643 | Proteobacteria Alphaproteobacteria |
| Magnetospirillum magneticum AMB-1 | NC_007626 | Proteobacteria Alphaproteobacteria |
| Caulobacter crescentus CB15 | NC_002696 | Proteobacteria Alphaproteobacteria |
| Rhodobacter sphaeroides 2.4.1 | NC_007488 NC_007493 NC_007494 NC_009007 | Proteobacteria Alphaproteobacteria |
| Paracoccus denitrificans PD1222 | NC_008686 NC_008687 NC_008688 | Proteobacteria Alphaproteobacteria |
| Jannaschia sp. CCS1 | NC_007801 NC_007802 | Proteobacteria Alphaproteobacteria |
| Roseobacter denitrificans OCh 114 | NC_008209 | Proteobacteria Alphaproteobacteria |
| Silicibacter pomeroyi DSS-3 | NC_003911 NC_006569 | Proteobacteria Alphaproteobacteria |
| Silicibacter sp. TM1040 | NC_008042 NC_008043 NC_008044 | Proteobacteria Alphaproteobacteria |
| Bradyrhizobium japonicum USDA 110 | NC_004463 | Proteobacteria Alphaproteobacteria |
| Nitrobacter hamburgensis X14 | NC_007959 NC_007960 NC_007964 | Proteobacteria Alphaproteobacteria |
| Nitrobacter winogradskyi Nb-255 | NC_007406 | Proteobacteria Alphaproteobacteria |
| Rhodopseudomonas palustris BisA53 | NC_008435 | Proteobacteria Alphaproteobacteria |
| Bartonella bacilliformis KC583 | NC_008783 | Proteobacteria Alphaproteobacteria |
| Bartonella henselae str. Houston-1 | NC_005956 | Proteobacteria Alphaproteobacteria |
| Bartonella quintana str. Toulouse | NC_005955 | Proteobacteria Alphaproteobacteria |
| Agrobacterium tumefaciens str. C58 | NC_003062 NC_003063 NC_003064 NC_003065 | Proteobacteria Alphaproteobacteria |
| Mesorhizobium loti MAFF303099 | NC_002678 NC_002679 NC_002682 | Proteobacteria Alphaproteobacteria |
| Brucella suis 1330 | NC_004310 NC_004311 | Proteobacteria Alphaproteobacteria |
| Brucella melitensis 16M | NC_003317 NC_003318 | Proteobacteria Alphaproteobacteria |
| Brucella abortus biovar 1 str. 9-941 | NC_006932 NC_006933 | Proteobacteria Alphaproteobacteria |
| Sinorhizobium meliloti 1021 | NC_003037 NC_003047 NC_003078 | Proteobacteria Alphaproteobacteria |

| Species | Genbank ID | Classification |
|---|---|---|
| Rhizobium etli CFN 42 | NC_007761 NC_007763 NC_007765 NC_007766 | Proteobacteria Alphaproteobacteria |
| Rhizobium leguminosarum bv. viciae 3841 | NC_008378 NC_008379 NC_008380 NC_008381 NC_008382 NC_008384 | Proteobacteria Alphaproteobacteria |
| Hyphomonas neptunium ATCC 15444 | NC_008358 | Proteobacteria Alphaproteobacteria |
| Maricaulis maris MCS10 | NC_008347 | Proteobacteria Alphaproteobacteria |
| Bdellovibrio bacteriovorus HD100 | NC_005363 | Proteobacteria Deltaproteobacteria |
| Lawsonia intracellularis PHE/MN1-00 | NC_008011 NC_008013 NC_008014 | Proteobacteria Deltaproteobacteria |
| Desulfovibrio desulfuricans G20 | NC_007519 | Proteobacteria Deltaproteobacteria |
| Desulfovibrio vulgaris subsp. vulgaris DP4 | NC_008751 | Proteobacteria Deltaproteobacteria |
| Anaeromyxobacter dehalogenans 2CP-C | NC_007760 | Proteobacteria Deltaproteobacteria |
| Myxococcus xanthus DK 1622 | NC_008095 | Proteobacteria Deltaproteobacteria |
| Desulfotalea psychrophila LSv54 | NC_006138 | Proteobacteria Deltaproteobacteria |
| Syntrophus aciditrophicus SB | NC_007759 | Proteobacteria Deltaproteobacteria |
| Syntrophobacter fumaroxidans MPOB | NC_008554 | Proteobacteria Deltaproteobacteria |
| Pelobacter carbinolicus DSM 2380 | NC_007498 | Proteobacteria Deltaproteobacteria |
| Geobacter sulfurreducens PCA | NC_002939 | Proteobacteria Deltaproteobacteria |
| Pelobacter propionicus DSM 2379 | NC_008607 NC_008609 | Proteobacteria Deltaproteobacteria |
| Geobacter metallireducens GS-15 | NC_007515 NC_007517 | Proteobacteria Deltaproteobacteria |
| Dehalococcoides ethenogenes 195 | NC_002936 | Dehalococcoides |
| Acidobacteria bacterium Ellin345 | NC_008009 | Acidobacteria Acidobacteria |
| Solibacter usitatus Ellin6076 | NC_008536 | Acidobacteria Acidobacteria |
| Bifidobacterium longum NCC2705 | NC_004307 | Actinobacteria Actinobacteria |
| Bifidobacterium adolescentis ATCC 15703 | NC_008618 | Actinobacteria Actinobacteria |
| Tropheryma whipplei str. Twist | NC_004572 | Actinobacteria Actinobacteria |
| Leifsonia xyli subsp. xyli str. CTCB07 | NC_006087 | Actinobacteria Actinobacteria |
| Arthrobacter aurescens TC1 | NC_008711 NC_008713 | Actinobacteria Actinobacteria |
| Streptomyces coelicolor A3(2) | NC_003888 NC_003903 | Actinobacteria Actinobacteria |
| Streptomyces avermitilis MA-4680 | NC_003155 | Actinobacteria Actinobacteria |
| Thermobifida fusca YX | NC_007333 | Actinobacteria Actinobacteria |
| Acidothermus cellulolyticus 11B | NC_008578 | Actinobacteria Actinobacteria |
| Frankia alni ACN14a | NC_008278 | Actinobacteria Actinobacteria |
| Corynebacterium efficiens YS-314 | NC_004369 | Actinobacteria Actinobacteria |

| Species | Genbank ID | Classification |
|---|---|---|
| Corynebacterium glutamicum ATCC 13032 | NC_003450 NC_006958 | Actinobacteria Actinobacteria |
| Corynebacterium jeikeium K411 | NC_007164 | Actinobacteria Actinobacteria |
| Corynebacterium diphtheriae NCTC 13129 | NC_002935 | Actinobacteria Actinobacteria |
| Mycobacterium avium 104 | NC_008595 | Actinobacteria Actinobacteria |
| Mycobacterium bovis AF2122/97 | NC_002945 | Actinobacteria Actinobacteria |
| Mycobacterium tuberculosis CDC1551 | NC_002755 | Actinobacteria Actinobacteria |
| Mycobacterium leprae TN | NC_002677 | Actinobacteria Actinobacteria |
| Mycobacterium ulcerans Agy99 | NC_008611 | Actinobacteria Actinobacteria |
| Mycobacterium vanbaalenii PYR-1 | NC_008726 | Actinobacteria Actinobacteria |
| Mycobacterium smegmatis str. MC2 155 | NC_008596 | Actinobacteria Actinobacteria |
| Nocardia farcinica IFM 10152 | NC_006361 NC_006363 | Actinobacteria Actinobacteria |
| Rhodococcus sp. RHA1 | NC_008268 NC_008271 | Actinobacteria Actinobacteria |
| Propionibacterium acnes KPA171202 | NC_006085 | Actinobacteria Actinobacteria |
| Nocardioides sp. JS614 | NC_008699 | Actinobacteria Actinobacteria |
| Rubrobacter xylanophilus DSM 9941 | NC_008148 | Actinobacteria Actinobacteria |
| Synechocystis sp. PCC 6803 | NC_000911 NC_005230 NC_005231 NC_005232 | Cyanobacteria Cyanobacteria |
| Trichodesmium erythraeum IMS101 | NC_008312 | Cyanobacteria Cyanobacteria |
| Nostoc sp. PCC 7120 | NC_003267 NC_003272 NC_003273 NC_003276 | Cyanobacteria Cyanobacteria |
| Anabaena variabilis ATCC 29413 | NC_007410 NC_007412 NC_007413 | Cyanobacteria Cyanobacteria |
| Synechococcus elongatus PCC 6301 | NC_006576 | Cyanobacteria Cyanobacteria |
| Prochlorococcus marinus str. AS9601 | NC_008816 | Cyanobacteria Cyanobacteria |
| Thermosynechococcus elongatus BP-1 | NC_004113 | Cyanobacteria Cyanobacteria |
| Gloeobacter violaceus PCC 7421 | NC_005125 | Cyanobacteria Cyanobacteria |
| Symbiobacterium thermophilum IAM 14863 | NC_006177 | Firmicutes Clostridia |
| Carboxydothermus hydrogenoformans Z-2901 | NC_007503 | Firmicutes Clostridia |
| Moorella thermoacetica ATCC 39073 | NC_007644 | Firmicutes Clostridia |
| Thermoanaerobacter tengcongensis MB4 | NC_003869 | Firmicutes Clostridia |
| Syntrophomonas wolfei subsp. wolfei str. Goettingen | NC_008346 | Firmicutes Clostridia |
| Desulfitobacterium hafniense Y51 | NC_007907 | Firmicutes Clostridia |
| Clostridium thermocellum ATCC 27405 | NC_009012 | Firmicutes Clostridia |
| Clostridium perfringens SM101 | NC_008262 NC_008264 | Firmicutes Clostridia |
| Clostridium perfringens ATCC 13124 | NC_008261 | Firmicutes Clostridia |

| Species | Genbank ID | Classification |
|---|---|---|
| Clostridium acetobutylicum ATCC 824 | NC_001988 NC_003030 | Firmicutes Clostridia |
| Clostridium tetani E88 | NC_004557 NC_004565 | Firmicutes Clostridia |
| Clostridium novyi NT | NC_008593 | Firmicutes Clostridia |
| Lactococcus lactis subsp. cremoris MG1363 | NC_009004 | Firmicutes Bacilli |
| Streptococcus mutans UA159 | NC_004350 | Firmicutes Bacilli |
| Streptococcus sanguinis SK36 | NC_009009 | Firmicutes Bacilli |
| Streptococcus pyogenes SSI-1 | NC_004606 | Firmicutes Bacilli |
| Streptococcus pyogenes M1 GAS | NC_002737 | Firmicutes Bacilli |
| Streptococcus agalactiae 2603V/R | NC_004116 | Firmicutes Bacilli |
| Streptococcus thermophilus CNRZ1066 | NC_006449 | Firmicutes Bacilli |
| Streptococcus pneumoniae D39 | NC_008533 | Firmicutes Bacilli |
| Geobacillus kaustophilus HTA426 | NC_006510 | Firmicutes Bacilli |
| Enterococcus faecalis V583 | NC_004668 NC_004669 NC_004670 NC_004671 | Firmicutes Bacilli |
| Listeria welshimeri serovar 6b str. SLCC5334 | NC_008555 | Firmicutes Bacilli |
| Listeria innocua Clip11262 | NC_003212 NC_003383 | Firmicutes Bacilli |
| Listeria monocytogenes EGD-e | NC_003210 | Firmicutes Bacilli |
| Listeria monocytogenes str. 4b F2365 | NC_002973 | Firmicutes Bacilli |
| Staphylococcus haemolyticus JCSC1435 | NC_007168 | Firmicutes Bacilli |
| Staphylococcus saprophyticus subsp. saprophyticus ATCC 15305 | NC_007350 | Firmicutes Bacilli |
| Staphylococcus aureus subsp. aureus N315 | NC_002745 | Firmicutes Bacilli |
| Staphylococcus epidermidis ATCC 12228 | NC_004461 NC_005005 NC_005007 | Firmicutes Bacilli |
| Bacillus clausii KSM-K16 | NC_006582 | Firmicutes Bacilli |
| Oceanobacillus iheyensis HTE831 | NC_004193 | Firmicutes Bacilli |
| Bacillus thuringiensis serovar konkukian str. 97-27 | NC_005957 NC_006578 | Firmicutes Bacilli |
| Bacillus anthracis str. Ames | NC_003997 | Firmicutes Bacilli |
| Bacillus anthracis str. 'Ames Ancestor' | NC_007322 NC_007323 NC_007530 | Firmicutes Bacilli |
| Bacillus cereus ATCC 10987 | NC_003909 NC_005707 | Firmicutes Bacilli |
| Bacillus cereus ATCC 14579 | NC_004722 | Firmicutes Bacilli |
| Bacillus anthracis str. Sterne | NC_005945 | Firmicutes Bacilli |
| Bacillus halodurans C-125 | NC_002570 | Firmicutes Bacilli |
| Bacillus licheniformis ATCC 14580 | NC_006270 NC_006322 | Firmicutes Bacilli |
| Bacillus subtilis subsp. subtilis str. 168 | NC_000964 | Firmicutes Bacilli |

| Species | Genbank ID | Classification |
| --- | --- | --- |
| Lactobacillus delbrueckii subsp. bulgaricus ATCC 11842 | NC_008054 | Firmicutes Bacilli |
| Lactobacillus delbrueckii subsp. bulgaricus ATCC BAA-365 | NC_008529 | Firmicutes Bacilli |
| Lactobacillus acidophilus NCFM | NC_006814 | Firmicutes Bacilli |
| Lactobacillus gasseri ATCC 33323 | NC_008530 | Firmicutes Bacilli |
| Lactobacillus johnsonii NCC 533 | NC_005362 | Firmicutes Bacilli |
| Lactobacillus salivarius subsp. salivarius UCC118 | NC_006529 NC_007930 NC_007929 | Firmicutes Bacilli |
| Lactobacillus casei ATCC 334 | NC_008502 NC_008526 | Firmicutes Bacilli |
| Lactobacillus sakei subsp. sakei 23K | NC_007576 | Firmicutes Bacilli |
| Lactobacillus plantarum WCFS1 | NC_004567 NC_006375 NC_006377 | Firmicutes Bacilli |
| Pediococcus pentosaceus ATCC 25745 | NC_008525 | Firmicutes Bacilli |
| Lactobacillus brevis ATCC 367 | NC_008497 NC_008498 | Firmicutes Bacilli |
| Oenococcus oeni PSU-1 | NC_008528 | Firmicutes Bacilli |
| Leuconostoc mesenteroides subsp. mesenteroides ATCC 8293 | NC_008496 NC_008531 | Firmicutes Bacilli |
